# Supplementary material for: Spatial localisation meets biomolecular networks
Source: Nat Commun. 2021 Sep 9;12:5357. doi: 10.1038/s41467-021-24760-y (PMC8429635; doi:10.1038/s41467-021-24760-y)
Supplement: Supplementary file 1 — Supplementary Information [file 41467_2021_24760_MOESM1_ESM.pdf]

Supplementary Information for:

Spatial localization meets biomolecular networks

Govind Menon[1] and J. Krishnan[1,2]

[1]Department of Chemical Engineering, Centre for Process Systems Engineering, and [2]Institute for Systems and Synthetic Biology, Imperial College London, South Kensington, London SW72AZ

Supplementary Information

In the following sections, we present both analytical arguments based on the mathematical models and additional computational results that support the main results of the study.

Summary of sections

1. **Supplementary Note 1: Basic effects of a localized component:** Here we analyse the basic location dependent and diffusivity dependent effects of localised components. We also present analytical results and additional computational results to support our discussion of new behaviour introduced by localisation in simple feedback motifs.
2. **Supplementary Note 2: Localisation induced instability:** Here we present numerical bifurcation analysis of a compartmental ODE model that complements the PDE model simulations and supports our discussion of the localisation induced instability of a homogenous steady state.
3. **Supplementary Note 3: Localised signal transduction for hybrid pattern forming mechanisms:** Here we present a more detailed discussion of the basic principle involved in hybrid pattern forming mechanisms triggered by gradient signals presented in the text.
4. **Supplementary Note 4: Two-diffusivity node: response to localised input:** Here we present analytical results supporting our discussion of the two-diffusivity node subject to localised regulation, resulting in a spatial "inverse" response. Analysis along the same lines is presented for the case where a negative feedback motif with a localised interaction acquires bistable characteristics. We also present further discussion of its consequences for inferring network interactions from experiments.
5. **Supplementary Note 5: Spatial control structures:** Here we present analysis supporting our discussion of a mechanism for adaptation of a target output at a given location, to changes in spatial separation. We also present a detailed discussion of how this mechanism can be combined with adaptation to an input signal level.
6. **Supplementary Note 6: Modular construction and spatial rewiring of multifunctional motifs:** Here we present additional computational results supporting our discussion of spatial rewiring to achieve modularity in multifunctional motifs with components shared between multiple feedback loops.
7. **Supplementary Note 7: Spatial regulation of Polo Kinase:** Here we present the models we use to examine two intracellular spatially distributed networks involving the spatial regulation of Polo kinase. We also provide further discussion of the results presented in the paper.
8. **Supplementary Note 8: Models and Parameter values:** Here we present the model equations (in Matlab code format) used to generate the figures and the corresponding parameter values used.

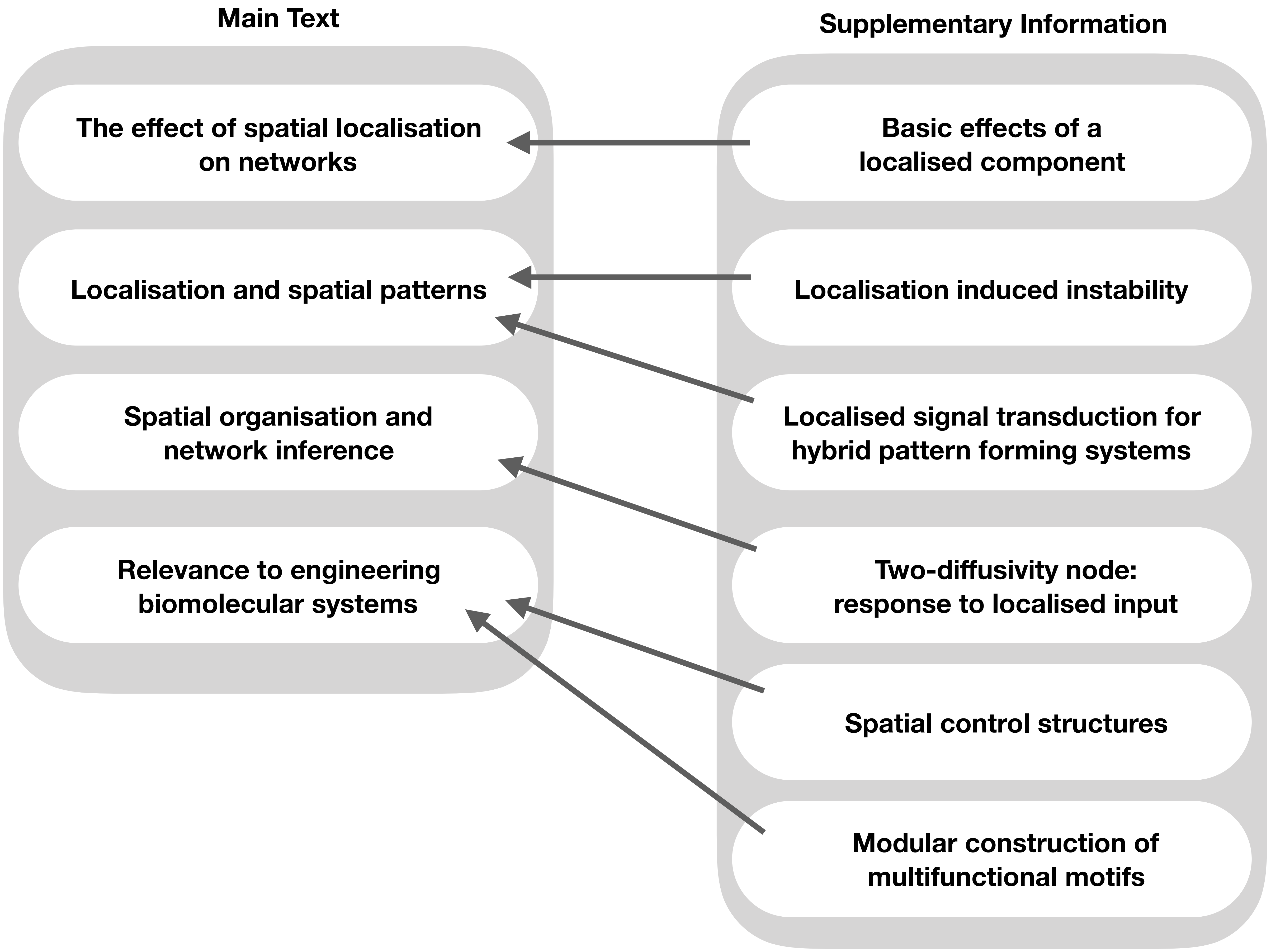

Supplementary Figure 0: Schematic layout of the supplementary information.

Contents

1 Supplementary Note 1: Basic effects of a localized component in a network containing local and global nodes 3
1.1 Single node with localised activation: analytical solution . . . 3
1.2 Location dependence of steady state output at input location . . . 4
1.2.1 Location dependence of bistability when one of the nodes is localised . . . 6
1.2.2 Location dependence of bistability when both nodes are localised . . . 7
1.3 Location dependence of steady state output average across the domain . . . 9
1.4 Effect of localisation on an adaptive IFF motif . . . 10
1.5 Localisation enables oscillatory behaviour in a two-node negative-feedback motif . . . 11
1.6 Effect of localisation on a bistable motif: bistable to tristable transition . . . 11

2 Supplementary Note 2: Localisation induced instability 12
2.1 Bifurcation analysis . . . 12
2.2 Placement adjacent to boundary: Two compartment model . . . 12
2.3 Placement at the centre: Three compartment model . . . 13

3 Supplementary Note 3: Localised information processing for hybrid pattern forming mechanisms 13
3.1 Regulation of pattern formation: homogeneous feedback from localised elements . . . 15

4 Supplementary Note 4: Two-diffusivity node - response to localised input 15
4.1 Two-diffusivity node with localised inhibition: analytical solution . . . 15
4.2 Two-diffusivity node with localised inhibition: basic effect . . . 16
4.3 Consequences for inferring the nature of interactions . . . 18
4.4 Consequences for inferring the nature of a circuit from qualitative dynamical characteristics . 20
4.5 Feedback loops containing two-diffusivity nodes regulated by localised interactions . . . 21
4.6 Deviant feedback when both fast and slow diffusing forms are active . . . 22

5 Supplementary Note 5: Spatial control structures 24
5.1 Open nodes with closed boundaries and uniform degradation . . . 25
5.1.1 Adaptation to spatial separation at a fixed 'target' location . . . 26
5.2 Adaptation to spatial separation: With closed nodes . . . 27
5.3 Combined adaptation to both signal level and spatial separation . . . 27
5.4 Localisation in Incoherent feedforward adaptation . . . 28

6 Supplementary Note 6: Modular construction of multifunctional motifs 28
6.1 Designing two node motifs: Localisation to circumvent kinetic constraints . . . 29

7 Supplementary Note 7: Spatial regulation of Polo Kinase 29
7.1 Transduction of cytoplasmic protein gradients by PLK1 . . . 29
7.1.1 Results . . . 30
7.2 Nuclear localization of Polo controlling mitotic entry . . . 31
7.2.1 Results . . . 34
7.2.2 Possibility of deviant behaviour in the Cdc25 - CycB-Cdk1 feedback loop . . . 34

Introduction

Systems biology presents multiple examples exhibiting spatial organization, realized through combinations of (highly) diffusible entities, weakly diffusible/non-diffusible entities as well as localization. Examples abound in bacteria, fungi and *C. elegans* of all these ingredients: the generation of intracellular gradients and the spatial regulation of cellular processes [25, 16, 34], polarity generation and the interaction with other parts

of signalling networks [11]; in some cases regulation of diffusivity is achieved in cells by chemical modification [33]. Important signalling pathways in eukaryotes, such as MAPK and calcium signalling exhibit multiple combinations of these ingredients as well [2, 4, 22]. The central importance of spatial organization is exemplified by studies on modules in fungi, revealing the presence of "spatial positive feedback" as part of a switch controlling mitosis onset [26], and compartmentalization of a bistable switch enabling intergenerational memory of pheromone exposure [6]. At the population level, similar ingredients are found in morphogenetic systems to achieve specific characteristics, and in multifunctional circuits in developmental pattern formation [12].Other population-level examples include the localization of different metabolic pathways in different cells, for instance Cynanobacterial filaments [20] or different spatial zones, for instance in the tumour microenvironment[5], the role of localization of metabolic pathways in determining tissue level insulin signalling response [9], and the combination of metabolic feedback interactions and quorum sensing in biofilms [21]. Other related studies spanning both cellular and tissue levels focus on the interplay of intracelllar polarization, tissue level patterning and tissue shape [8]

Another theme of considerable recent interest is phase- separated compartments/membrane-less organelles and their dynamic regulation in a variety of cellular contexts including adaptation to stress, transcriptional control, and noise reduction [30]. In many cases, they are thought to facilitate interactions by spatially co-localising components [30]. Their dynamic nature (in some cases regulated by post-translational modifications [24]) gives the possibility of dynamically changing/rewiring interaction networks, a feature that may also be exploited to create controllable synthetic membrane-less compartments [27].

The combination of the spatial ingredients (localized elements as well as diffusible and non/weakly diffusible elements) and networks is a basic recurrent theme in synthetic biology as well. A broad range of recent activity in synthetic biology ranging from the purely chemical to cellular engineering showcases the importance of these ingredients from both the bottom-up constructive, and the engineering perspectives. On one hand spatial engineering of biochemical systems in non-cellular environments has been developed in the context of engineering information processing [28, 29] as well as the creation of new materials [32]. The creation of sophisticated compartmentalized systems (and their communication) as basic ingredients of artificial cells (and in some cases natural cells) is the focus of a broad body of work [7, 13]. Other studies either incorporate synthetic circuits into natural cells, or reconstitute elements of natural circuits in non-cellular environments [3]. At the population level, the engineering of synthetic population mimics, as well as engineering synthetic circuits for programming multicellular structures, morphogenesis and morphological changes all employ these basic ingredients [1, 13]. Other examples, situated at the junction of synthetic and systems biology, include engineering cellular decision making in the context of tissue engineering [18].

1 Supplementary Note 1: Basic effects of a localized component in a network containing local and global nodes

The most basic effects of having a localised node in a network are revealed in the context of a single localised node regulating a downstream (output) node of intermediate diffusivity (the non-diffusing and high diffusing cases can then be examined as limiting cases of this). These effects are of two basic kinds: (1) location dependence - where the steady state concentration of the output node (domain average or concentration at the input location) can depend on the location of the localised node relative to the closed boundaries (2) diffusivity dependence - where the steady state concentration of the output node (domain average or concentration at a particular location) can depend on the diffusivity of the output node. We start by examining location dependence.

1.1 Single node with localised activation: analytical solution

For a single (diffusible) node subject to localised activation (uniform input signal within the localised subdomain $[\theta_1, \theta_2]$), the model equations are as follows: For $\theta \in [0, \theta_1)$,

$$\frac{\partial [X^*]}{\partial t} = -k_2 X^* + D_{X^*} \frac{\partial^2 [X^*]}{\partial \theta^2}$$
$$\frac{\partial [X]}{\partial t} = k_2 X^* + D_X \frac{\partial^2 [X]}{\partial \theta^2}$$

For $\theta \in [\theta_1, \theta_2]$,

$$\frac{\partial [X^*]}{\partial t} = k_1 S X - k_2 X^* + D_{X^*} \frac{\partial^2 [X^*]}{\partial \theta^2}$$
$$\frac{\partial [X]}{\partial t} = -k_1 S X + k_2 X^* + D_X \frac{\partial^2 [X]}{\partial \theta^2}$$

For $\theta \in (\theta_2, 1]$,

$$\frac{\partial [X^*]}{\partial t} = -k_2 X^* + D_{X^*} \frac{\partial^2 [X^*]}{\partial \theta^2}$$
$$\frac{\partial [X]}{\partial t} = k_2 X^* + D_X \frac{\partial^2 [X]}{\partial \theta^2}$$

For a fixed initial total amount of the modified forms in the domain, X_T, and assuming $D_{X^*} = D_X$, and no-flux boundary conditions at boundaries $\theta = 0$ and $\theta = 1$, the steady-state solution is given by:

For $\theta \in [0, \theta_1)$,

$$X^* = c_1 \cosh(\omega_2 \theta)$$

For $\theta \in [\theta_1, \theta_2]$,

$$X^* = \frac{k_1 S X_T}{k_1 S + k_2} + c_2 \cosh(\omega_{12}\theta) + c_3 \sinh(\omega_{12}\theta)$$

For $\theta \in (\theta_2, 1]$,

$$X^* = c_4 \cosh(\omega_2(1 - \theta))$$

where $\omega_{12} = \sqrt{\frac{k_1 S + k_2}{D_{X^*}}}$, and $\omega_2 = \sqrt{\frac{k_2}{D_{X^*}}}$. The constants of integration c_i may be analytically computed by solving the set of four linear equations in c_i obtained by imposing the boundary conditions at θ_1 and θ_2: continuity of the profile and its first derivative, i.e. flux. The c_i were computed using symbolic algebraic tools in Maple. The analytical solution is cross-checked against the numerical solution.

1.2 Location dependence of steady state output at input location

In the text we showed how different measures of the steady state output depend on the location of a localised input. We first focus on a specific measure of the output - the output concentration at the input location. In this case, the location dependence is most pronounced when the steady state level of the output node is far from saturation, i.e. we have a system whose model resembles that of an open system (For the same reason, the location dependence of the domain average is least significant in this regime: it is easy to see that the domain average in the case of an open system is independent of location). Therefore, we will examine the system in this regime, to demonstrate this result analytically. (We note that this can also be done analytically by examining the general steady state solution for the system, without any assumption of limiting regime). For the output node X, in the far from saturation regime, with localised activation over $\theta \in [\theta_1, \theta_2]$ in a 1-D domain of $L = 1$ with uniform phosphatase, the simplified model is as follows:

For $\theta \in [0, \theta_1)$,

$$\frac{\partial [X^*]}{\partial t} = -k_2 X^* + D_{X^*} \frac{\partial^2 [X^*]}{\partial \theta^2}$$

For $\theta \in [\theta_1, \theta_2]$,

$$\frac{\partial [X^*]}{\partial t} = k_1 S X_T - k_2 X^* + D_{X^*} \frac{\partial^2 [X^*]}{\partial \theta^2}$$

For $\theta \in [\theta_2, 1)$,

$$\frac{\partial [X^*]}{\partial t} = -k_2 X^* + D_{X^*} \frac{\partial^2 [X^*]}{\partial \theta^2}$$

where S denotes the concentration of the localised input signal. This was obtained by the approximation $X \approx X_T$, noting that, owing to $D_X = D_{X^*}$, $X + X^* = X_T$ everywhere. The steady-state solution is given by:

For $\theta \in [0, \theta_1)$,

$$X^* = \frac{k_1 S X_T \cosh(\omega\theta)}{k_2 \sinh(\omega)} \left(\sinh(\omega(1-\theta_1)) - \sinh(\omega(1-\theta_2))\right)$$

For $\theta \in [\theta_1, \theta_2]$,

$$X^* = \frac{k_1 S X_T \cosh(\omega\theta)}{k_2 \sinh(\omega)} \left(\sinh(\omega(1-\theta)) - \sinh(\omega(1-\theta_2))\right) + \frac{k_1 S X_T \cosh(\omega(1-\theta))}{k_2 \sinh(\omega)} \left(\sinh(\omega\theta) - \sinh(\omega\theta_1)\right)$$

For $\theta \in [\theta_2, 1)$,

$$X^* = \frac{k_1 S X_T \cosh(\omega(1-\theta))}{k_2 \sinh(\omega)} \left(\sinh(\omega\theta_2) - \sinh(\omega\theta_1)\right)$$

where $\omega = \sqrt{\frac{k_2}{D_{X^*}}}$. This full solution was obtained by solving the relevant ODEs, along with the matching conditions for the concentrations and flux at $\theta = \theta_1$ and $\theta = \theta_2$ as outlined above.

Let the compartment of size l be at location p (midpoint of the compartment; total domain size = 1). At steady state, the concentration profile has a varying gradient outside the compartment, with gradient equal to zero at the two boundaries. For simplicity, we will examine the case where the compartment size is small relative to the size of the domain ($l << 1$). In this case, the steady concentration profile of X^* is relatively flat within the compartment, and can be approximated by its average x_c. Now, the removal of the species from the compartment happens via diffusion out of the compartment through the boundaries. The steady state mass balance for the compartment thus has the form:

$$l k_1 S X_T - l k_2 x_c - \mathrm{R}_{\text{boundaries}} = 0$$

where the first term represents localised conversion of X to X^*, the second term represents conversion of X^* to X within the compartment, and the third term represents the net outflow through the compartment boundaries. From the solution presented above, for $l << 1$, the compartmental average (approximated by the concentration at the midpoint) is given by:

$$x_c \approx \frac{2 k_1 S X_T \sinh(\frac{\omega l}{2}) \cosh(\omega p) \cosh(\omega(1-p))}{k_2 \sinh(\omega)}$$

where $\omega = \sqrt{\frac{k_2}{D_{X^*}}}$. Again, using the analytical solution and neglecting appropriate terms for $l << 1$, the total removal rate of the species through the boundaries is approximately given by:

$$\begin{aligned}\mathrm{R}_{\text{boundaries}} &\approx \frac{k_1 S X_T D_{X^*} \omega \sinh(\omega l)}{k_d \sinh(\omega)} \left((\cosh(\omega(1-p)) \sinh(\omega p) + \sinh(\omega(1-p)) \cosh(\omega p)\right) \\ &= \left[\frac{D_{X^*} \omega \sinh(\omega l)}{2 \sinh(\frac{\omega l}{2})} \left(\frac{\sinh(\omega p)}{\cosh(\omega p)} + \frac{\sinh(\omega(1-p))}{\cosh(\omega(1-p))}\right)\right] x_c\end{aligned}$$

Using this approximation for the effective outflow of the species , the steady state mass balance for the compartment thus has the form:

$$l(k_1 S X_T - k_2^{eff} x_c) = 0$$

where

$$k_2^{eff} = \left[k_2 + \frac{D_{X^*}\omega \sinh(\omega l)}{2l\sinh(\frac{\omega l}{2})}\left(\frac{\sinh(\omega p)}{\cosh(\omega p)} + \frac{\sinh(\omega(1-p))}{\cosh(\omega(1-p))}\right)\right] \quad (1)$$

The compartmental output average at steady state is again given by $x_c = \frac{k_1 S X_T}{k_2^{eff}}$. In this case, we see that k_2^{eff} has a minimum when the input node is placed close to the boundaries, and is maximum when it is placed at the centre. This can be seen both by plotting k_2^{eff} as a function of p, noting that it is symmetric about the midpoint $p = 0.5$, further verified by differentiating with respect to p Correspondingly, the output level in the compartment is highest when the input node is close to the boundaries and lowest when it is at the centre.

In the above analysis, we have essentially taken the PDE model for the single node with localised activation, and used it to obtain a "quasi-ODE" type description by examining the far from saturation limit, combined with the limit of small compartment size relative to the total domain size. This "quasi-ODE" description, which is valid for the localised subdomain, captures the spatial features of the system (i.e. the effect of input location relative to boundaries) through an "effective rate constant" of the reverse reaction that depends on the input location relative to boundaries.

1.2.1 Location dependence of bistability when one of the nodes is localised

In the main text we discuss an example where the the location of a localised component (in this case one of the nodes) relative to boundaries affects the characteristic information processing behaviour of a motif - in this case a two node mutual activation motif exhibiting bistability. While the computational result shown is in the general case, we analyse this behaviour here by examining the far from saturation limit for the diffusible node, combined with the limit of small compartment size relative to the total domain size. As above, this allows us to obtain a "quasi-ODE" description, which is valid for the localised subdomain, captures the location dependent behaviour through an "effective rate constant" of the reverse reaction that depends on the input location relative to boundaries. Since we are examining a bistable system here, we note that using the far from saturation limit assumes that all three coexisting steady states correspond to far from saturation levels of the diffusible node output across the domain.

The two node motif considered here involves mutual activation between a diffusible node X and a localised node Y. This is described by the following system of equations: For $\theta \in [0, \theta_1)$,

$$\frac{\partial[X^*]}{\partial t} = -k_2 X^* + D_{X^*}\frac{\partial^2[X^*]}{\partial\theta^2}$$
$$\frac{\partial[X]}{\partial t} = k_2 X^* + D_X\frac{\partial^2[X]}{\partial\theta^2}$$

For $\theta \in [\theta_1, \theta_2]$,

$$\frac{\partial[X^*]}{\partial t} = k_1 Y^* X - k_2 X^* + D_{X^*}\frac{\partial^2[X^*]}{\partial\theta^2}$$
$$\frac{\partial[X]}{\partial t} = -k_1 Y^* X + k_2 X^* + D_X\frac{\partial^2[X]}{\partial\theta^2}$$
$$\frac{\partial[Y^*]}{\partial t} = k_3\frac{X^{*2}}{1+X^{*2}}Y - k_4 Y^*$$
$$\frac{\partial[Y]}{\partial t} = -k_3\frac{X^{*2}}{1+X^{*2}}Y + k_4 Y^*$$

For $\theta \in (\theta_2, 1]$,

$$\frac{\partial[X^*]}{\partial t} = -k_2 X^* + D_{X^*}\frac{\partial^2[X^*]}{\partial\theta^2}$$
$$\frac{\partial[X]}{\partial t} = k_2 X^* + D_X\frac{\partial^2[X]}{\partial\theta^2}$$

For a fixed (uniform) initial total concentration of the modified forms across the domain, X_T (and fixed Y_T in the localised subdomain), we can eliminate the concentrations of the inactive forms X and Y: For $\theta \in [0, \theta_1)$,

$$\frac{\partial [X^*]}{\partial t} = -k_2 X^* + D_{X^*} \frac{\partial^2 [X^*]}{\partial \theta^2}$$

For $\theta \in [\theta_1, \theta_2]$,

$$\begin{aligned}
\frac{\partial [X^*]}{\partial t} &= k_1 Y^* (X_T - X^*) - k_2 X^* + D_{X^*} \frac{\partial^2 [X^*]}{\partial \theta^2} \\
\frac{\partial [Y^*]}{\partial t} &= k_3 \frac{X^{*2}}{1 + X^{*2}} (Y_T - Y^*) - k_4 Y^*
\end{aligned}$$

For $\theta \in (\theta_2, 1]$,

$$\frac{\partial [X^*]}{\partial t} = -k_2 X^* + D_{X^*} \frac{\partial^2 [X^*]}{\partial \theta^2}$$

At steady state, for X^* far from saturation, we have (in the localised subdomain): For $\theta \in [\theta_1, \theta_2]$,

$$\begin{aligned}
k_1 Y^* X_T - k_2 X^* + D_{X^*} \frac{\partial^2 [X^*]}{\partial \theta^2} &= 0 \\
k_3 \frac{X^{*2}}{1 + X^{*2}} (Y_T - Y^*) - k_4 Y^* &= 0
\end{aligned}$$

In the limit of small compartment size relative to the total domain size, the concentration profiles of both X* and Y* within the subdomain are essentially flat, and can be approximated by their average levels. Let x and y represent these average steady state levels. By combining the far from saturation limit for the X node, with the limit of small compartment size relative to the total domain size, we can also replace the diffusing term in the X^* equation with a first order removal term exactly as before. Thus, we have:

$$k_1 y X_T - k_2^{eff} x = 0 \tag{2}$$

$$k_3 \frac{x^2}{1 + x^2} (Y_T - y) - k_4 y = 0 \tag{3}$$

where k_2^{eff} is given by (1), and depends on the location of the localised Y node relative to the boundaries. By eliminating y, it is easy to see that (3) has three real positive roots only for

$$(k_1 k_3 X_T Y_T)^2 > 4 \left(k_2^{eff}\right)^2 k_4 (k_3 + k_4)$$

As seen from (1), k_2^{eff} is lowest for localisation close to the boundaries, and increases towards the center of the domain. Thus, the inequality above indicates that the system can be pushed out of the bistable regime by having the localised node Y closer to the centre of the domain. This result is computationally demonstrated in the general case (i.e. not small compartment size or X^* far from saturation) in the main text.

1.2.2 Location dependence of bistability when both nodes are localised

We now consider how location dependent behaviour can be examined in the case of the above motif with both nodes (X and Y) localised separately. This of course requires diffusible communicating species. Let a

diffusible node Z mediate the activation of Y by X and second diffusible node W mediate the activation of X by Y (both forms equally diffusible for Z and W). As before, for a fixed (uniform) initial total concentration of the modified forms across the domain: fixed X_T and fixed Y_T in the localised subdomains, fixed Z_T and W_T across the whole domain, we can eliminate the concentrations of the inactive forms, which yields the following system of equations for X localised at $[p_1 - l/2, p_1 + l/2]$ and Y localised at $[p_2 - l/2, p_2 + l/2]$: For $\theta \in [0, p_1 - l/2)$,

$$\begin{aligned}\frac{\partial[Z^*]}{\partial t} &= -k_{dz}Z^* + D_{Z^*}\frac{\partial^2[Z^*]}{\partial\theta^2}\\ \frac{\partial[W^*]}{\partial t} &= -k_{dw}W^* + D_{W^*}\frac{\partial^2[W^*]}{\partial\theta^2}\end{aligned}$$

For $\theta \in [p_1 - l/2, p_1 + l/2]$,

$$\begin{aligned}\frac{\partial[X^*]}{\partial t} &= k_1W^*(X_T - X^*) - k_2X^*\\ \frac{\partial[Z^*]}{\partial t} &= k_zX^*(Z_T - Z^*) - k_{dz}Z^* + D_{Z^*}\frac{\partial^2[Z^*]}{\partial\theta^2}\\ \frac{\partial[W^*]}{\partial t} &= -k_{dw}W^* + D_{W^*}\frac{\partial^2[W^*]}{\partial\theta^2}\end{aligned}$$

For $\theta \in (p_1 + l/2, p_2 - l/2)$,

$$\begin{aligned}\frac{\partial[Z^*]}{\partial t} &= -k_{dz}Z^* + D_{Z^*}\frac{\partial^2[Z^*]}{\partial\theta^2}\\ \frac{\partial[W^*]}{\partial t} &= -k_{dw}W^* + D_{W^*}\frac{\partial^2[W^*]}{\partial\theta^2}\end{aligned}$$

For $\theta \in [p_2 - l/2, p_2 + l/2]$,

$$\begin{aligned}\frac{\partial[Z^*]}{\partial t} &= -k_{dz}Z^* + D_{Z^*}\frac{\partial^2[Z^*]}{\partial\theta^2}\\ \frac{\partial[W^*]}{\partial t} &= k_wY^*(W_T - W^*) - k_{dw}W^* + D_{W^*}\frac{\partial^2[W^*]}{\partial\theta^2}\\ \frac{\partial[Y^*]}{\partial t} &= k_3\frac{Z^{*2}}{1 + Z^{*2}}(Y_T - Y^*) - k_4Y^*\end{aligned}$$

For $\theta \in (p_2 + l/2, 1]$,

$$\begin{aligned}\frac{\partial[Z^*]}{\partial t} &= -k_{dz}Z^* + D_{Z^*}\frac{\partial^2[Z^*]}{\partial\theta^2}\\ \frac{\partial[W^*]}{\partial t} &= -k_{dw}W^* + D_{W^*}\frac{\partial^2[W^*]}{\partial\theta^2}\end{aligned}$$

By considering the limit of small compartment size relative to the subdomain, and the far from saturation limit for the diffusing species, we can obtain a "quasi-ODE" description as before, which at steady state gives us:

$$k_1w(X_T - x) - k_2x = 0$$

$$k_3\left(\frac{z^2}{1 + z^2}\right)(Y_T - y) - k_4y = 0$$

where x and y are the average steady state levels of X^* and Y^* in their respective subdomains/compartments, w is the average steady state level of W^* in the X compartment, and z is the average steady state level of Z^* in the Y compartment. In the limit of small compartment size and far from saturation for nodes Z and W, z and w can be related to x and y as:

$$z = \frac{4\sinh^2(\frac{\omega_z l}{2})}{lk_{dz}\omega_z \sinh(\omega_z)} \cosh(\omega_z p_1)\cosh(\omega_z(1-p_2))k_z x Z_T$$
$$w = \frac{4\sinh^2(\frac{\omega_w l}{2})}{lk_{dw}\omega_w \sinh(\omega_w)} \cosh(\omega_w(1-p_2))\cosh(\omega_w p_1)k_w y W_T$$

where $\omega_z = \sqrt{\frac{k_{dz}}{D_{Z^*}}}$ and $\omega_w = \sqrt{\frac{k_{dw}}{D_{W^*}}}$. Invoking the small compartment limit allows us to capture the production of Z^* and W^* in terms of the compartmental averages x and y, while the far from saturation limit makes it simpler to obtain the expression for the spatial average of Z^* and W^* in the Y and X compartments reapectively. See section 6.1 below for a more detailed step-by-step discussion of these expressions. Thus, we again have a "quasi-ODE" description, where steady state location dependent behaviour is essentially captured by a kinetic type description. This result, combined with the previous ones, illustrates how the analysis of a distributed system of nodes and interactions containing both diffusing, non-diffusible, and localised elements can be systematically reduced to the analysis of an "ODE" type description by using the small compartment limit and the far from saturation limit - the spatial features being captured essentially through an "effective" kinetic parameter. This type of analysis is valid more generally, and can be used to analyse other motifs, for instance the negative feedback motif with both interactions localised, discussed below.

1.3 Location dependence of steady state output average across the domain

The effect on the average steady state output across the domain is most pronounced when the steady state level of the output node is close to saturation at the input location. Therefore, we will examine the system in this regime. (We note that this can also be done by examining the general steady state solution for the system). In this regime, the steady state output level at the input location is essentially independent of location, and has a value close to X_T (the total concentration of X and X*). The location dependence, of the average of X* across the domain, therefore arises essentially due to the change in concentration profile outside the input location. This profile can be calculated (for $\theta \in [0, \theta_1)$ and $\theta \in (\theta_2, 1]$) by fixing the concentration at θ_1 and θ_2 to be X_T.

For $\theta \in [0, \theta_1)$, the profile has the form

$$X^* = \frac{X_T \cosh(\omega\theta)}{\cosh(\omega\theta_1)}$$

and for $\theta \in (\theta_2, 1]$,

$$X^* = \frac{X_T \cosh(\omega(1-\theta))}{\cosh(\omega(1-\theta_2))}$$

Thus, the average outside the compartment can be calculated to be:

$$\langle X^* \rangle = \frac{X_T}{\omega(1-l)} (\tanh(\omega\theta_1) + \tanh(\omega(1-\theta_2)))$$

For a thin localised region, we have $\theta_1 \approx \theta_2 = p$, in which case

$$\langle X^* \rangle = \frac{X_T}{\omega(1-l)} (\tanh(\omega p) + \tanh(\omega(1-p)))$$

It can be seen that the above function of p has a single maximum at $p = 0.5$ (it is symmetric about $p = 0.5$) and is lowest at the boundaries ($p = 0, 1$).

1.4 Effect of localisation on an adaptive IFF motif

The case of an adaptive incoherent feedforward motif with a moderately diffusible output node, and the two arms of the feedforward regulation localised, is discussed in the main text. We now discuss in greater detail, how localisation affects motif behaviour. Consider the purely temporal realisation of the motif described by an ODE model:

$$\frac{dX^*}{dt} = k_1 S(X_T - X^*) - k_2 X^*$$
$$\frac{dY^*}{dt} = k_3 S(Y_T - Y^*) - k_4 Y^*$$
$$\frac{dZ^*}{dt} = k_5 X^*(Z_T - Z^*) - k_6 Y^* Z^*$$

where S is the input signal. For adaptation of steady state Z^* to the signal, the X^* and Y^* at steady state have to be essentially proportional to the signal level, i.e. these nodes must be far from saturation. In this regime, the steady state Z^* is given by:

$$Z^* = \frac{k_5 Z_T(\alpha S)}{k_5(\alpha S) + k_6(\beta S)}$$

where $X^* = \alpha S, Y^* = \beta S$, with α, β easily determined in terms of the kinetic constants above. Both numerator and denominator are proportional to S, and adaptation results from this cancellation.

Consider the spatial realisation of the motif where we have incorporated a moderately diffusible output node (Z) and uniform signal, we see that localisation of nodes X and Y can affect the adaptive capability of the motif, i.e. it exhibits adaptation when they are co-localised, and fails to adapt when they are apart. The former case is essentially analogous to the ODE case - the output node is not involved in any interactions outside the localised subdomain containing nodes X and Y, and the steady state profile of the output node is uniform across the domain. The latter case can be examined using a two compartment compartmental ODE model (which also corresponds to the PDE when the compartments are small [19]), with X and Y confined to different equally sized compartments, but subject to the same level of input signal S:

In compartment 1,

$$\frac{dX^*}{dt} = k_1 S(X_T - X^*) - k_2 X^*$$
$$\frac{dZ_1^*}{dt} = k_5 X^*(Z_T - Z^*) - tr(Z_1^* - Z_2^*)$$

In compartment 2,

$$\frac{dY^*}{dt} = k_3 S(Y_T - Y^*) - k_4 Y^*$$
$$\frac{dZ_2^*}{dt} = -k_6 Y^* Z_2^* - tr(Z_2^* - Z_1^*)$$

where tr is the transport parameter. Again assuming that the X and Y nodes are far from saturation, the steady state output levels are given by:

$$Z_1^* = \frac{k_5 Z_T(\alpha S)}{k_5(\alpha S) + \frac{k_6 tr(\beta S)}{tr + k_6(\beta S)}}$$
$$Z_2^* = \frac{tr}{tr + k_6(\beta S)} \left[\frac{k_5 Z_T(\alpha S)}{k_5(\alpha S) + \frac{k_6 tr(\beta S)}{tr + k_6(\beta S)}} \right]$$

Thus, the cancellation of the signal in the ODE model is not seen for either Z_1^* or Z_2^* individually, except in a regime where the transport parameter tr is very high. The average output level is given by:

$$Z_{avg}^* = \frac{1}{2}\left[\left(1+\frac{tr}{tr+k_6tr(\beta S)}\right)\frac{k_5 Z_T(\alpha S)}{k_5(\alpha S)+\frac{k_6tr(\beta S)}{tr+k_6(\beta S)}}\right]$$

This was obtained by assuming two thin compartments at the two end of the domain Again, we see that there is no cancellation of the input signal level, except at high tr. Thus, when the output node is only moderately diffusible, the adaptive capability is compromised by localising nodes X and Y at different locations.

1.5 Localisation enables oscillatory behaviour in a two-node negative-feedback motif

In the case of a two node negative feedback motif, it can be shown analytically, using Bendixson's negative criterion (Dulac's criterion), that the corresponding ODE system cannot have a limit cycle. The two variable ODE system takes the following form:

$$\frac{dX^*}{dt} = F(X^*, Y^*) = k_1(X_T - X^*) - k_2h_1(Y^*)X^*$$
$$\frac{dY^*}{dt} = G(X^*, Y^*) = k_3h_2(X^*)(Y_T - Y^*) - k_4Y^*$$

where $h_1(Y^*)$ and $h_2(X^*)$ are Hill functions. In a given (closed, simply connected) region of phase space, Bendixson's criterion rules out the existence of a closed orbit for this system if $\frac{\partial F}{\partial X^*} + \frac{\partial G}{\partial Y^*} \neq 0$ at every point in this region (i.e $\frac{\partial F}{\partial X^*} + \frac{\partial G}{\partial Y^*}$ never becomes zero). These derivatives are given by:

$$\frac{\partial F}{\partial X^*} = -k_1 - k_2h_1(Y^*)$$
$$\frac{\partial G}{\partial Y^*} = -k_3h_2(X^*) - k_4$$

As X^* and Y^* are concentrations, and consequently non-negative, these derivatives are guaranteed to be negative. Thus, $\frac{\partial F}{\partial X^*} + \frac{\partial G}{\partial Y^*}$ cannot be zero at any point, and the existence of a limit cycle is ruled out.

However, as shown in Supplementary Figure 5(A), with the two interactions localised at different locations, the same system (with same kinetic parameters) is seen to exhibit limit cycle oscillations for intermediate diffusivities of the two nodes. The emergence of oscillatory behaviour resulting from interactions at different spatial locations coupled by diffusive transport has also been studied in other intracellular contexts [31].

1.6 Effect of localisation on a bistable motif: bistable to tristable transition

Here we comment on the extent to which it is possible to switch from one steady state to another via the external signal. For the purpose of this discussion, we name the three stable steady states of the distributed system as follows: ss1 is the high X^*, low Y^* steady state, ss2 is the "mixed" steady state, and ss3 is the low X^*, high Y^* steady state. If the external signal is varied uniformly across the domain, we find that this might allow for the switching from ss1 to ss2 or ss3 to ss2 but not between ss1 and ss3. This is illustrated by the bifurcation diagram shown in Supplementary Figure 1(C). This is in contrast to the ODE realisation of the system, where the same signal can bring about for switching between the the high X^*, low Y^* steady state and the low X^*, high Y^* steady state. This aspect highlights another contrast between the purely temporal realisation a distributed realisation of the same motif with fixed kinetics. On the other hand, varying the external signal inhomogeneously can facilitate switching between steady states ss1 and ss3.

2 Supplementary Note 2: Localisation induced instability

For any motif with all nodes diffusible, co-localising the two interconversion reactions at any one of the nodes (including reactions that aren't regulated by another node) produces a system that has a homogeneous steady state identical to that of the corresponding ODE model (total amounts of species in the ODE correspond to local total amount of corresponding species in the PDE, the latter being constant in space when the two species in a given node have the same diffusivity). This can be seen by analysing the PDE model as follows:

- Inside the localised subdomain the kinetics are identical to the ODE model, so a uniform state (all species uniform) where the X*, Y*, Z* levels are identical to the ODE steady state would cause the time derivatives of X*, Y*, Z* to be zero (kinetic part and diffusion part are each individually zero).
- Outside the localised subdomain, Z* must be uniform at steady state, as it is not subject to any reactions (only diffusion). The X* and Y* kinetics (including the effect of Z* on Y*) are identical to the ODE model. So a uniform state (all species uniform) where the X*, Y*, Z* levels are identical to the ODE steady state would again cause the time derivatives of X*, Y*, Z* to be zero in this region (kinetic part and diffusion part are each individually zero).

However, as discussed in the text, this essentially 'ODE' steady state may lose stability depending on the size of the localization, even when all the nodes are equally diffusible. In these regimes, the system has stable inhomogeneous stable steady states (Fig.4, main text). Even in parameter regimes where the homogeneous steady state is stable, the system can have coexisting stable inhomogeneous steady states (and even stable limit cycles). Simulations of the PDE model indicate that the number and nature of these steady states can depend on the position of the localisation relative to boundaries. For instance, localisation in the centre of the domain (dividing the domain into identical halves) can give rise to a pair of symmetry broken, inhomogeneous steady states, with output level high in one half and low in the other. In certain regimes these steady states can coexist with other inhomogeneous steady states where the symmetry is not broken. In contrast, when the localisation is adjacent to a boundary, other types of inhomogeneous steady states are obtained.

Note:

- For the motif examined here (see Fig. 4), the positive feedback loop involving X and Y considered in isolation, is monostable for the chosen parameter values.
- The addition of node Z and its associated interactions to this positive feedback loop does not introduce bistability, for the chosen parameter values. This is demonstrated by bifurcation analysis with the total amount at node Z as the bifurcation parameter (Supplementary Figure 3).

2.1 Bifurcation analysis

All of the above results are supported by bifurcation analysis of the corresponding compartmental ODE models, with the transport parameter as the bifurcation parameter (Supplementary Figure 3). (All kinetic parameters take the same values in both the full PDE model and the compartmental ODE models). The case of localisation at the centre of the domain is captured by a three compartment model, with the localised reactions confined to the middle compartment, while localisation adjacent to a boundary is captured by a two compartment model, with localised reactions confined to one of the compartments. In both cases we assume for simplicity that all transport parameters and reaction compartment sizes are equal.

2.2 Placement adjacent to boundary: Two compartment model

The analysis shows that that as the transport parameter is reduced, the homogeneous steady state loses stability through a transcritical bifurcation when localisation is at a boundary (Supplementary Figure 3(A)). One of the steady state branches (corresponding to high output level away from the compartment containing the localised reactions) also undergoes a subcritical Hopf bifurcation. The transcritical bifurcation involves an exchange of stability between a uniform steady state and an inhomogeneous steady state which

corresponds to high output level away from the compartment containing the localised reactions. This inhomogeneous steady state is connected to another inhomogeneous steady state branch corresponding to high output level away from the compartment containing the localised reactions through a saddle node bifurcation.

2.3 Placement at the centre: Three compartment model

Here the analysis shows that that as the transport parameter is reduced, the homogeneous steady state loses stability through a subcritical pitchfork bifurcation (Supplementary Figure 3(B)). The two branches of unstable steady states emerging from the pitchfork bifurcation undergo saddle-node bifurcations, forming two branches corresponding to symmetry broken, stable steady states. We see that these steady states can coexist with the stable homogeneous steady states over a range of transport parameter. As the transport parameter is reduced further, we see that the branch of homogeneous steady state(s) undergoes a further bifurcation: a transcritical bifurcation similar to the previous case. In this instance, the homogeneous steady state remains unstable. The the inhomogeneous steady state branch connecting with this point (also unstable) is characterised by low outside. If we follow this branch in the direction of increasing transport paramenter, this branch as before undergoes a saddle node bifurcation connecting to another inhomogeneous ss branch characterised by,. Following this branch further, we find a subcritical pitchfork bifurcation allowing this inhomogeneous but symmetry preserving steady state to be stable (this being a steady state with high output outside the localisation compartment). In a similar vein, following the inhomogeneous steady state at the transcritical bifurication in the other direction (decreasing transport parameter) gives rise to a subcritical pitchfork bifurcation, thereby conferring stability on this inhomogeneous (but symmetry respecting) steady state characterised by low output outside the localised compartment.

To summarise, the bifurcation structure is such that, for even lower values of transport parameter, the system has four distinct coexisting steady stable states: two of these are symmetry broken inhomogeneous steady states and two are symmetry respecting inhomogeneous steady states. We note that corresponding coexisting steady states, and corresponding parameter ranges for their stability are also observed computationally by varying diffusivity in the full PDE model.

3 Supplementary Note 3: Localised information processing for hybrid pattern forming mechanisms

In the main text we discuss the possibility of constructing a hybrid pattern forming system that combines gradient driven pattern forming with a Turing mechanism. We demonstrate how transduction of a gradient signal by distinct localised nodes at multiple (two) locations, followed by the combined regulation of a common (global) element by these localised nodes can allow for a mechanism that triggers pattern formation in response to gradient inputs, while preventing it for uniform signals. Here we describe this mechanism in detail:

- The main pattern forming component of the system is an activator-inhibitor motif with slow diffusing activator and fast diffusing inhibitor, i.e. with the basic ingredients for a Turing instability.
- The above pattern forming motif is regulated by a global node - so that the output level of this node is essentially a parameter in the Turing pattern forming motif.
- The above global node is subject to combined and antagonistic regulation by two localised nodes, both of which are regulated (activated) by a common input signal. The antagonistic regulation involves one of the localised nodes activating, and the other inhibiting the global node. It is assumed that the diffusivity of the global node is high enough for it to be essentially uniform at steady state, even in the presence of the "source-sink" separation generated by the antagonistic regulation. This feature is key to preventing the pattern from being distorted by a gradient.
- With such antagonistic regulation, it is possible to have generic values for kinetic parameters (even without introducing any additional nonlinearity in the kinetics) that will ensure that a uniform signal cannot cause the steady state level of the global node to cross a certain threshold. For instance, a very

simple way of realising this would be to have (1) the output of both the localised nodes proportional to the signal level, and (2) have basal interconversion rates for the global node, which are uniform across the domain.Implementing such a model indicates that the output node depends monotonically on the signal level when it is uniform. This implies, and further saturates as well. In this case it can be analytically shown that in multiple parameter regimes, a uniform input signal cannot cause the steady state level of the global node to cross a certain threshold (eg: if increasing signal pushes the output further away from the threshold). We briefly demonstrate this for the model of antagonistic regulation used in our example. The global node Z is subject to regulation by two nodes localised apart- activation by X and inhibition by Y. For simplicity, we consider the limit where nodes X and Y are far from saturation, so that their output levels are proportional to the local signal level at their location. Since Z is highly diffusible, and is subject to uniform basal levels of interconversion across the domain, its (essentially homogeneous) steady state output has the form:

$$Z^* = \frac{aX^* + b}{aX^* + cY^* + d} Z_T$$

where X^* and Y^* represent the local (essentially uniform within their subdomain) steady state levels of the output at nodes X and Y. Since these are proportional to the local signal level, we have:

$$Z^* = \frac{\alpha I_X + b}{\alpha I_X + \gamma I_Y + d}$$

where I_X and I_Y are the input signal levels in the X and Y compartments respectively. This means that a spatially non-uniform signal (with $I_X \neq I_Y$) can cause this output level to drop below the basal (zero signal) level given by b/d. However, if we consider a uniform signal, i.e. $I_X = I_Y = I$,

$$Z^* = \frac{\alpha I + b}{\alpha I + \gamma I + d}$$

and we see that the derivative of Z^* with respect to the signal level has the form:

$$\frac{dZ}{dI} = \frac{\alpha d - (\alpha + \gamma)b}{(\alpha I + \gamma I + d)^2}$$

which means that for a suitable range of parameters, this derivative will be positive irrespective of signal level, thus guaranteeing that a uniform signal cannot cause the output level to drop below the basal level.

- If the threshold for triggering pattern formation is below b/d, we see therefore that a uniform signal cannot lead to a crossing of the threshold.
- A spatially graded input signal, can remove the above constraint by favouring one of the two localised nodes, and allow the global node to cross the threshold, potentially moving the downstream pattern forming motif into the parameter regime where the homogeneous steady state is unstable.
- Such antagonistic regulation involving a "push-pull" mechanism is common in multiple biological contexts, and forms a basic ingredient of the above hybrid pattern forming mechanism.
- The above mechanism, where a signal transduced locally at multiple locations is then combined at single node via global elements, that acts as an input to the pattern forming module, represents a specific case/basic example of a more general mechanism - one that enables locally transduced signal levels from multiple locations to feed into a single pattern forming module. Such combined regulation can possibly determine whether a pattern forms or even certain features of the pattern, such as wavelength or amplitude, but crucially, without introducing any spatial variation in these features that reflect either the spatial profile of the signal or the localised elements transducing it.

3.1 Regulation of pattern formation: homogeneous feedback from localised elements

In the main text, we present a prototypical example of a system where added feedback regulation of a pattern forming motif through a combination of localised and global elements biases the phase of the steady state pattern formed. This mechanism, which is another example makes the pattern essentially independent of initial conditions, without introducing any distortions in the pattern itself. With regard to this system we make the following points:

- The pattern forming motif considered in isolation (without the feedback interaction) is in a parameter regime where it has a stable homogeneous steady state.
- Increasing the rate constant of the reverse reaction at the inhibitor node Z (i.e. Z to Z*), while keeping the remaining parameters fixed can push the system into a pattern forming regime (where the homogenous steady state is unstable).
- The feedback regulation of pattern phase presented here directly builds on this feature, as follows.
- First, by making node Y inhibit node Z, the reverse reaction at node Z now depends on the output level at node Y.
- Second, this inhibition is realised through a combination of two other nodes, one localised and the other global node, such that the localised node samples the local level of Y output (i.e. it is activated by Y*), and feeds this information to the global node (i.e. the localised node activates the global node), which then inhibits the Z node uniformly across the domain.
- The above regulatory structure ensures that the uniform inhibition of Z is sufficiently high to allow pattern formation if the local output level of node Y at the location of the localised node is high enough.
- Thus, for the chosen parameter values, a pattern where Y has a trough at this location cannot be a stable steady state for the system - the resulting inhibition of node Z would be insufficient to keep the system in the pattern forming regime.
- Note that, in this regime, the system has a uniform steady state which is locally stable. A transient spatial cue - we consider localised spatial cues here - can trigger pattern formation - i.e. a transition to a spatially inhomogeneous steady state - depending on its amplitude and location (see Fig. 5(g)).

4 Supplementary Note 4: Two-diffusivity node - response to localised input

In the paper, we discuss the response of a single node with low diffusing active form and high diffusing inactive form to a localised input - see Figure 6 (d-g).

4.1 Two-diffusivity node with localised inhibition: analytical solution

For a single two-diffusivity node subject to localised inhibition at the location $[\theta_1, \theta_2]$, along with uniform basal levels of activation and inhibition, the model equations are as follows:

For $\theta \in [0, \theta_1)$,

$$\frac{\partial [X^*]}{\partial t} = k_{01}X - k_{02}X^* + D_{X^*}\frac{\partial^2 [X^*]}{\partial \theta^2}$$
$$\frac{\partial [X]}{\partial t} = -k_{01}X + k_{02}X^* + D_X\frac{\partial^2 [X]}{\partial \theta^2}$$

For $\theta \in [\theta_1, \theta_2]$,

$$\frac{\partial[X^*]}{\partial t} = k_{01}X - k_{02}X^* - k_2X^* + D_{X^*}\frac{\partial^2[X^*]}{\partial\theta^2}$$
$$\frac{\partial[X]}{\partial t} = -k_{01}X + k_{02}X^* + k_2X^* + D_X\frac{\partial^2[X]}{\partial\theta^2}$$

For $\theta \in (\theta_2, 1]$,

$$\frac{\partial[X^*]}{\partial t} = k_{01}X - k_{02}X^* + D_{X^*}\frac{\partial^2[X^*]}{\partial\theta^2}$$
$$\frac{\partial[X]}{\partial t} = -k_{01}X + k_{02}X^* + D_X\frac{\partial^2[X]}{\partial\theta^2}$$

Note that, at steady state, the concentration profiles of X and X^* satisfy the following relationship at all locations in the domain $[0, 1]$:

$$\frac{\partial^2}{\partial\theta^2}\left(D_{X^*}[X^*] + D_X[X]\right) = 0$$

Thus, for the steady state solution, we can introduce new variables $u = D_{X^*}[X^*]$ and $v = D_X[X]$, so that $u + v = \chi$ where χ is uniform across the domain. This directly follows from the above equation, and the no-flux boundary conditions for X and X*. In the unifrom diffusivity case, χ would essentially correspond to the uniform 'total concentration', i.e. $\frac{\textit{total amount}}{\textit{domain size}}$ multiplied by the diffusivity. However, note that χ here depends on the parameters that determine the steady state profiles (including kinetic parameters), and is not completely determined by specifying the total amount and domain size.

The change of variables yields the following system of equations for the profiles of u and v:

For $\theta \in [0, \theta_1)$,

$$k'_{01}v - k'_{02}u + \frac{\partial^2 u}{\partial\theta^2} = 0$$
$$-k'_{01}v + k'_{02}u + \frac{\partial^2 v}{\partial\theta^2}$$

For $\theta \in [\theta_1, \theta_2]$,

$$k'_{01}v - k'_{02}u - k'_2u + \frac{\partial^2 u}{\partial\theta^2} = 0$$
$$-k'_{01}v + k'_{02}u + k'_2u + \frac{\partial^2 v}{\partial\theta^2} = 0$$

For $\theta \in (\theta_2, 1]$,

$$k'_{01}v - k'_{02}u + \frac{\partial^2 u}{\partial\theta^2} = 0$$
$$-k'_{01}v + k'_{02}u + \frac{\partial^2 v}{\partial\theta^2} = 0$$

It is easy to see that this has the same form as the equal diffusivity case. The steady state profiles for the variables u and v can therefore be calculated using the solution to the equal diffusivity case (presented in section 1.1), with $D_{X^*} = D_X = 1$. χ appears as a multiplicative factor in these profiles, and can be related to the fixed total amount by averaging these profiles across the whole domain.

4.2 Two-diffusivity node with localised inhibition: basic effect

In the main text, we see how increasing the concentration of a localised phosphatase (in the presence of fixed levels of global kinase and phosphatase) can cause the steady state level of the active form to locally *increase* at locations outside of the localised domain, while *lowering* the average level of the active form

across the domain. The analytical steady state solution of the PDE model discussed above can be used to analyse this basic effect. For simplicity, we focus on the limiting case, where the low-diffusing active form is non-diffusible, and the high-diffusing inactive form has very high diffusivity (ensuring that its steady state profile is essentially uniform).

In this limit, the steady state concentration profile for the high-diffusing inactive form is essentially uniform across the domain, while the active form has a discontinuous profile, with uniform profiles at different levels, inside and outside the localised subdomain, and a discontinuity at the boundaries of the subdomain. Such a discontinuity would be absent with weak diffusivity of the active form, but the essential insights of that case can be revealed here. At steady state, let x denote the spatially uniform concentration of the inactive form, x^*_{in} and x^*_{out} denote the spatially uniform concentrations of the active form inside and outside the localised subdomain respectively. Let L denote the size of the whole spatial domain and w denote the size of the subdomain. Let the total amount in the pool of modified forms be x_T.

We see that the following relationships are valid at steady state. Within the subdomain:

$$k_{01}x - (k_{02} + k_2)x^*_{in} = 0$$

Outside the compartment:

$$k_{01}x - k_{02}x^*_{out} = 0$$

The fixed total amount imposes the following constraint:

$$Lx + wx^*_{in} + (L - w)x^*_{out} = x_T$$

This allows us to compute x^*_{out}:

$$x^*_{out} = \frac{x_T}{\frac{k_{02}L}{k_{01}} + \frac{k_{02}w}{k_{02}+k_2} + (L - w)}$$

From this expression, it is easy to see that, as the rate constant k_2 (corresponding to the localised inhibition) increases, the denominator decreases, and consequently x^*_{out} increases. Similarly, if we compute the average output across the whole domain:

$$\begin{aligned} x^*_{avg} &= \frac{1}{L}(wx^*_{in} + (L - w)x^*_{out}) \\ &= \frac{1}{L}(x_T - Lx) = \frac{1}{L}(x_T - \frac{k_{02}L}{k_{01}}x^*_{out}) \end{aligned}$$

Since x^*_{out} is the only term here that depends on k_2, and increases with increasing k_2, it is easy to see that the average must decrease.

We also present an analysis of this basic effect using a compartmental ODE model for the system. We consider a two compartment model with equal sized compartments. Such a system corresponds to having the localised inhibition subdomain adjacent to a boundary in the full PDE model. The steady state equations are given by:

$$\begin{aligned} k_1X_1 - k_2X^*_1 - k_pX^*_1 - p_s(X^*_1 - X^*_2) = 0 \\ -k_1X_1 + k_2X^*_1 + k_pX^*_1 - p(X_1 - X_2) = 0 \end{aligned}$$

in compartment 1, which contains the localised phosphatase (corresponding to rate constant k_p), and

$$\begin{aligned} k_1X_2 - k_2X^*_2 - p_s(X^*_2 - X^*_1) = 0 \\ -k_1X_2 + k_2X^*_2 - p(X_2 - X_1) = 0 \end{aligned}$$

where the rate constants k_1 and k_2 correspond to the action of a global kinase and global phosphatase (uniformly distributed). p_s and p represent the transport parameters associated with X^* and X respectively. The total amount $X_T = X + X*$ needs to be fixed to find a particular steady state for the above system. This yields the equation:

$$X_1 + X_1^* + X_2 + X_2^* = X_T$$

For the case of interest, the transport parameter for the active form X^* is low, while the transport parameter for X is high. We analytically solve for the steady state, and examine X_2^*, i.e. the level of active form away from the location of the localised phosphatase.

$$X_2^* = \frac{k_1 X_T \left(pk_2 + pk_p + 2\,pp_s + k_1 p_s\right)}{2pk_1k_2 + pk_1k_p + 4pk_1p_s + 2pk_2{}^2 + 2pk_2k_p + 4pk_2p_s + 2pk_pp_s + 2k_1{}^2p_s + 2k_1k_2p_s + k_1k_pp_s}$$

To examine the effect of increasing the level of localised phosphatase, we compute the derivative with respect to the parameter k_p.

$$\frac{dX_2^*}{dk_p} = \frac{\left(\left(k_1 - 2\,p_s\right)p - k_1p_s\right)k_1X_T\ \left(\left(k_2 + 2\,p_s\right)p + k_1p_s\right)}{4\left(\left(\left(k_2 + 1/2\,k_p + 2\,p_s\right)k_1 + \left(2\,k_2 + k_p\right)p_s + k_2\left(k_2 + k_p\right)\right)p + k_1p_s\left(k_1 + k_2 + 1/2\,k_p\right)\right)^2}$$

The denominator is positive, and the numerator is given by

$$num = X_T\ \left(2\,p + k_1\right)\left(-k_1 - 2\,p\right)k_1p_s{}^2 + \left(X_T\,pk_2\left(-k_1 - 2\,p\right) + X_T\ \left(2\,p + k_1\right)pk_1\right)k_1p_s + X_T\,p^2k_2k_1{}^2$$

The three terms in the numerator are of the same order in p, and arranged in decreasing powers of p_s. Thus, for low p_s and high p, this expression is dominated by the last term, which is positive. Thus, an increase in k_p (corresponding to an increase in localised phosphatase) results in an increase in X_2^*. A similar analysis indicates that an increase in k_p reduces the average level of X^* across the two compartments, irrespective of the values of p_s and p.

4.3 Consequences for inferring the nature of interactions

The above results, i.e., the opposing trends (increase in one and decrease in the other) exhibited by different measures of the output, have the following implications for inferring the nature of interactions between network components using data from local or averaged measurements (for specificity, we assume that, as above, the active form is the slow diffusing form, and the localised input has an inhibitory effect, i.e. brings about the active to inactive transition):

- **Inferring interaction between the localised input and the two-diffusivity node:** The average (over the whole domain) measurements of both the input level and the output active form exhibit trends that are consistent with the actual relationship between input and output,i.e. the average output level decreases in response to increasing average input level. Localised measures of the two at the input location also exhibit consistent trends. However, localised measures of the two at different locations, i.e. the input level measured in the localised subdomain and the output measured outside of this subdomain, can exhibit trends that are inconsistent with the actual input-output relationship. In this case the output measure can increase in response to increasing the level of the localised input, contrary to the inhibitory nature of the input.The last observation is particularly relevant in cases where the output node cannot be measured directly. If the output level is instead being inferred from one of its downstream effects (for instance by measuring the level of a downstream signal whose actual relationship to the output is established), the presence of further spatial organization or even just nonlinear effects like cooperativity in the associated downstream reactions, can cause these downstream signals to accentuate and reflect these local trends in the output of interest, which might not appear to reflect the nature of the interaction.

- **Inferring interaction between the two-diffusivity node and a downstream node:** In this case, even the average (over the whole domain) measurements of the outputs at the two nodes may not reflect their actual relationship (i.e. how the output of the two-diffusivity node affects the output of the downstream node). For instance, consider a case where the actual relationship is activating,

and the interaction involves nonlinear threshold type behaviour. Suppose the output level of the two-diffusivity node is experimentally controlled by increasing the level of the localised inhibitor considered above. While the average measure of this output is guaranteed to decrease in response to this input, it is possible that the average level of the downstream output can, in fact, increase, contrary to the functional relationship between the nodes, i.e. activation. This could very easily result from a combination of the local increase in the output of the two-diffusivity node, and the nonlinear (threshold) effect involved in its activation of the downstream node. This is a simple consequence of the fact that spatial averaging and nonlinearity do not commute.

The two cases above highlight the fact that inferring an activatory or inhibitory relationship purely from the positive or negative correlation between measures of an input and output node can be fundamentally misleading, even in the context of a simple network like a basic signal transduction cascade. Importantly, we see that such pitfalls may be present even when using average measures for all the nodes of interest. Overall, these results indicate that it may be vital to account for spatial organisation/localisation when inferring network interactions from data.

- **Inferring an interaction where none exists**: The main text (Discussion section) presents examples where an interaction may be inferred in a network (by ignoring localization), while none exists. There are multiple examples of this:
  - A simple example is inferring an interaction based on in-vitro data, but the interaction not occurring in vivo, because the two components are in different locations.
  - Similarly an interaction may be relevant in some contexts but not others: for instance two components may interact in a certain phase of the cell cycle (when they are in the same spatial compartment) but not in another phase (when they are in different spatial compartments). Here one type of data may be used to infer something about the system (in a slightly different context) which may be incorrect.
  - Consider two nodes regulated by a common element S. Depending on the data, a direct (or indirect) regulation of A and B by S, may be inferred as an interaction between A and B. If A and B are in different compartments, they may not have any interactions, but if this possibility is ignored, then an incorrect inference may be made. Similar conclusions hold good if the interaction between A and B (in different compartments) is indirect (eg through shared global components).Other indirect effects (eg mechanical effects) could be incorrectly inferred as an interaction between A and B, especially if those effects or factors are outside the postulated framework. Having different compartments allows common components to function in different ways. An example (discussed in [17]) is of a bifunctional enzyme which can act (primarily) as a kinase in one compartment and phosphatase in another compartment at the same time. This dual behaviour is directly enabled by different compartments. If the possibility of different compartments is ignored, then such a possibility may not be accounted for. This then could result in an incorrect inference. For instance suppose the signal activates A in one compartment and inhibits B in another compartment, for instance by being a kinase in the first compartment and a phosphatase in the second compartment. Furthermore suppose experiments (based on measurements in the first compartment) establish the kinase activity. Then the observed steady state regulation could be mis-inferred as the signal activates A and A inhibits B.
  - A similar conclusion could be made at the cell population level where common factors could have different regulatory properties in different cell types (eg. resistant cell vs wild-type cell). The assumption of homogeneity would rule that out, and could result in an incorrect inference.
  - We have already shown the feature of an "inverse response" wherein one node (A) regulating another (two-diffusivity) node (B) can result in B exhibiting an opposite type of response in certain regions/locations (eg A inhibiting B can result in an elevation of B in certain regions). Suppose B regulates another node C present at such a location. Then by ignoring localization, and working in an ODE framework would result in either (a) inferring an opposite type of regulation of C by B or (ii) postulating another regulation of C to explain the data, which is non-existent.

– We have already shown how by ignoring spatial localization (of interactions), a negative feedback circuit could be inferred as a positive feedback circuit. This then means that if the intermediate component in the feedback loop is regulating another network component, that the nature of the regulation would be incorrectly inferred. Therefore to explain the correct observed behaviour of that other component would likely involve another interaction which would be an incorrect inference.
– We have already seen in the paper that for certain types of measurements localization can create a qualitative discrepancy between the actual behaviour and that of the ODE (with the same interactions). Having to account for the data in an ODE framework may then involve the inference of other (non-existing) interactions to make up for this discrepancy.
– We also point out that a component may regulate the localization of another component and this could be incorrectly inferred as a direct interaction. For instance, we have shown how negative feedback circuit (involving a two-diffusivity node) with one interaction localized could be inferred as a positive-feedback circuit. Now suppose an external node X was causing the localization of this interaction. From the data it would appear (for eg by knocking out X) that X was responsible for the bistability, and consequently playing a direct regulatory role.

4.4 Consequences for inferring the nature of a circuit from qualitative dynamical characteristics

Building on the above results, we have constructed an instance of a two-node negative feedback loop, containing one two-diffusivity node (as considered above) and one global node (equal, high diffusivity for both forms). For the purpose of this discussion, we refer to the former as node X and the latter as node Y. The negative feedback loop involves the following interactions: the output of node X activates node Y, and the output of node Y inhibits node X. If no component is localised, the behaviour of the motif is consistent with that of a negative feedback loop - it has a single stable steady state, as shown by bifurcation analysis.

However, keeping all the parameters fixed, if the regulation of node X (i.e. inhibition) by node Y is localised - by confining this reaction to a subdomain - we see that the same system can exhibit bistability. Thus, the negative feedback loop essentially exhibits a characteristic associated with a positive feedback loop. This behaviour essentially results from combining threshold type behaviour with the effect discussed above i.e., localised increase of the output of X caused by a localised inhibition. We analyse this behaviour as above, in the limit where the low-diffusing active form X^* is non-diffusible, and the inactive form X is highly diffusible. Both forms of node Y are also assumed to have high (and equal) diffusivity.

In this limit, X, Y, and Y^* are uniform across the whole domain, while X^* has a discontinuous profile as described previously. As before, at steady state, let x denote the spatially uniform concentration of the inactive form, x^*_{in} and x^*_{out} denote the spatially uniform concentrations of the active form inside and outside the localised subdomain respectively. Let y^* denote the spatially uniform concentration of Y^* across the domain. Let L denote the size of the whole spatial domain and w denote the size of the subdomain. Let the total amount in the pool of modified forms be x_T and y_T.

We have the following relationships at steady state. Within the subdomain:

$$k_{01}x - (k_{02} + k_2 h(y^*))x^*_{in} = 0$$
$$k_y g(x^*_{in})(y_T - y^*) - k_{dy} y^* = 0$$

Outside the compartment:

$$k_{01}x - k_{02}x^*_{out} = 0$$
$$k_y g(x^*_{out})(y_T - y^*) - k_{dy} y^* = 0$$

Here, h and g are Hill functions.

The fixed total amount imposes the following constraint:

$$Lx + wx^*_{in} + (L-w)x^*_{out} = x_T$$
$$\implies x^*_{out} = \frac{x_T}{\frac{k_{02}L}{k_{01}} + \frac{k_{02}w}{k_{02}+k_2 h(y^*)} + (L-w)}$$

Consider the steady state relationships between x^*_{out} and y^*. We have:

$$x^*_{out} = \frac{x_T}{\frac{k_{02}L}{k_{01}} + \frac{k_{02}w}{k_{02}+k_2 h(y^*)} + (L-w)}$$

and

$$y^* = \frac{k_y g(x^*_{out}) y_T}{k_y g(x^*_{out}) + k_{dy}}$$

The above relationships can be compared to the case without localisation, where x^* is uniform, and we have:

$$x^* = \frac{k_{01} x_T}{k_{01} + k_{02} + k_2 h(y^*)}$$
$$y^* = \frac{k_y g(x^*) y_T}{k_y g(x^*) + k_{dy}}$$

The latter cannot produce multiple steady states - the curves defined by these relationships cannot have multiple points of intersection, while in the former case, the corresponding curves can have multiple (three) intersections - see Supplementary Figure 4. These curves can be seen as analogous to the nullclines of a two node feedback system. Supplementary Figure 4(B) depicts the (expected) nullcline structure for a negative feedback motif, while (D) shows a nullcline structure associated with a positive feedback motif.

4.5 Feedback loops containing two-diffusivity nodes regulated by localised interactions

As seen above (and in the paper - see Figure 7) having a two-diffusivity node regulated by a localised interaction within a feedback loop can cause the system to exhibit deviant behaviour even causing a negative feedback loop to exhibit the characteristics of a positive feedback loop and vice versa. However, if other interactions within the feedback loop are also localised, it is possible that the nature of the feedback remains unaffected. This depends on which of the other interactions is localised, and in which location. We elucidate this point here. In doing this we generalize our analysis and aim to establish when and in what way, feedback circuits containing a two-diffusivity node exhibit unexpected behaviour.

We consider two node feedback loops with one of the nodes having fast and slow diffusing forms, and the other node having both forms (equally) fast diffusing, and examine possible behaviour when interactions of the feedback loop are localised in some spatial region, either together or apart. We discuss the possible behaviour below, and present further analysis to support our conclusions, along the lines of the analysis presented above for the deviant behaviour of the negative feedback. We note that the following conclusions apply to both negative and positive feedback. Note that when we consider cases where interactions are localised apart, we assume that they are sufficiently far apart to prevent any "interference" between the two locations and the localisation therein. For the purposes of our discussion, we also assume that the for fast diffusing form, gradients in the steady state profile are negligible.

In addition to the two different ways of localising the two interactions (co-localised or localised apart) such two node feedback loops can also be divided into two classes along another axis - depending on which form (slow diffusing or fast diffusing) of the two-diffusivity node is the major (or possibly only) regulator of the other node (in the negative feedback example considered above, the slow diffusing form regulates the other node, and the fast diffusing form has no activity). Thus overall we have four possible realisations, and we describe their behaviour below (summarised in the table in Fig.7(g)):

- Colocalised interactions with slow diffusing form as major regulator of other node: In this case the anomalous effect of the localised regulation on the activity of the two diffusivity node (localised inhibition causing increase in activity/ localised activation causing reduction in activity), and the regulation by the two-diffusivity node of the other node are essentially separated in space. The anomalous effect on the activity of the two diffusivity node occurs outside the domain of localisation, while the interaction through which the other node is regulated occurs within the domain of localisation. Thus, the nature of the feedback is unaffected, but the feedback loop can have an anomalous effect on the activity

of the two-diffusivity node outside the domain of localisation - for instance the steady state activity outside the domain of localisation is increased by the presence of the negative feedback, relative to the case with no feedback. This corresponds to the upper left quadrant in the table in Fig 7 (g): the nature of feedback is maintained — but as summarized above, anomalous behaviour can be observed in some part of the spatial domain.

- Colocalised interactions with fast diffusing form as major regulator of other node: In this case, if the slow diffusing form has no activity, there is no possibility of an anomalous effect. Such an effect is possible however, if the slow diffusing form also has some activity (i.e. it is also regulating the other node), albeit at a lower level than the fast diffusing form. In this case, the anomalous effect of the localised regulation on the total activity (activity of both forms together) of the two diffusivity node (localised inhibition causing increase in activity/ localised activation causing reduction in activity), and the regulation by the two-diffusivity node of the other node are essentially at the same spatial location. The anomalous effect on the activity of the two diffusivity node occurs within the domain of localisation, while the interaction through which the other node is regulated also occurs within the domain of localisation. Thus, the nature of the feedback can be inverted - for instance a negative feedback loop can exhibit the characteristics of a positive feedback. This result is shown in Fig. 7(i), and supported by the analysis below. This corresponds to the upper right quadrant of the table in Fig. 7(g).
- Interactions localized apart with slow diffusing form as major regulator of the other node: In this case the anomalous effect of the localised regulation on the activity of the two diffusivity node (localised inhibition causing increase in activity/ localised activation causing reduction in activity), and the regulation by the two-diffusivity node of the other node are essentially at the same spatial location. The anomalous effect on the activity of the two-diffusivity node occurs outside the domain where it is regulated by the other node. The regulation of the other node by the two-diffusivity node also occurs in the same location. A consequence of this is that the nature of the feedback can be inverted - the deviant behaviour of a negative feedback demonstrated previously is a variation of this case where, instead of having the two interactions localised apart, only the regulation of the two-diffusivity node is localised. This corresponds to the lower left quadrant in the table in Fig. 7(g).
- Interactions localized apart with fast diffusing form as major regulator of other node: In this case, if the slow diffusing form has no activity, there is no possibility of an anomalous effect. An anomalous effect is possible only if the slow diffusing form also has some activity (i.e. it is also regulating the other node), albeit at a lower level than the fast diffusing form. In this case the anomalous effect of the localised regulation on the activity of the two diffusivity node (localised inhibition causing increase in activity/ localised activation causing reduction in activity), and the regulation by the two-diffusivity node of the other node are essentially separated in space. The anomalous effect on the activity of the two-diffusivity node occurs within the domain where it is regulated by the other node. However, the regulation of the other node by the two-diffusivity node occurs outside this domain. Thus, the nature of the feedback is unaffected, but the feedback loop can have an anomalous effect on the activity of the two-diffusivity node in the domain where this node is regulated by the other node - for instance the steady state activity within in this domain may increased by the presence of the negative feedback, relative to the case with no feedback. This corresponds to the lower right quadrant in the table in Fig. 7(g).

4.6 Deviant feedback when both fast and slow diffusing forms are active

We analyse this behaviour in the same way as for the negative feedback above where only the slow diffusing form at the two-diffusivity node affects the other node. Again we consider a two-node negative feedback loop containing one two-diffusivity node (as considered above) and one global node (equal, high diffusivity for both forms). For the purpose of this discussion, we refer to the former as node X and the latter as node Y. The negative feedback loop involves the following interactions: both the fast diffusing for X and the slow diffusing form X^* activate node Y (convert Y to Y^*), with X having higher activity than X^*; Y^* inhibits the activity of node X by converting X to X^*. If no component is localised, the behaviour of the motif is

consistent with that of a negative feedback loop. However, with both interactions colocalised, the system can exhibit positive feedback characteristics, including bistability. We demonstrate this below (see Fig.7 (i)).

For the analysis, we consider the limit where the low-diffusing less active form X^* is non-diffusible, and the more active form X is highly diffusible. Both forms of node Y are also assumed to have high (and equal) diffusivity.

In this limit, X, Y, and Y^* are uniform across the whole domain, while X^* has a discontinuous profile as described previously. As before, at steady state, let x denote the spatially uniform concentration of the active form, x^*_{in} and x^*_{out} denote the spatially uniform concentrations of the less active form inside and outside the localised subdomain respectively. Let y^* denote the spatially uniform concentration of Y^* across the domain. Let L denote the size of the whole spatial domain and w denote the size of the subdomain. Let the total amount in the pool of modified forms be x_T and y_T.

We have the following relationships at steady state, with the parameter γ capturing the relative activity of the slow diffusing form with respect to the fast diffusing form ($\gamma < 1$). We use the same notations as above for the rate constants of the reaction. Within the subdomain:

$$k_{01}x - k_{02}x^*_{in} + k_2 h(y^*)x = 0$$
$$k_y g(x + \gamma x^*_{in})(y_T - y^*) - k_{dy}y^* = 0$$

Outside the compartment:

$$k_{01}x - k_{02}x^*_{out} = 0$$

Here, h and g are Hill functions.

The fixed total amount imposes the following constraint:

$$Lx + wx^*_{in} + (L - w)x^*_{out} = x_T$$
$$\implies x^*_{in} = \frac{(xT - (L + (L - w)(\frac{k_{01}}{k_{02}}))x)}{w}$$

Consider the steady state relationships between x and y^*. We have:

$$x = \frac{x_T - wx^*_{in}}{L + (L - w)\frac{k_{01}}{k_{02}}}$$

where x^*_{in} can be expressed as a function of y^* alone, and

$$y^* = \frac{k_y g(x + \gamma x^*_{in})y_T}{k_y g(x + \gamma x^*_{in}) + k_{dy}}$$

where x^*_{in} can be expressed as a function of x alone. The above relationships can be compared to the case without localisation, where x^* is uniform, and we have:

$$x^* = x_T - \frac{k_{01} + k_2 h(y^*)}{k_{01} + k_{02} + k_2 h(y^*)}$$
$$y^* = \frac{k_y g(x + \gamma(x_T - x))y_T}{k_y g(x + \gamma(x_T - x)) + k_{dy}}$$

The latter cannot produce multiple steady states - the curves defined by these relationships cannot have multiple points of intersection, while in the former case, depending on the activity ratio γ, the corresponding curves can have multiple (three) intersections - see Fig.7(i). These curves can be seen as analogous to the nullclines of a two node feedback system.

5 Supplementary Note 5: Spatial control structures

For a single (moderately diffusible) node subject to localised activation in the region $[\theta_1, \theta_2]$, the steady state level of its output at a particular location/subdomain depends on the separation between the two locations (region of localised activation and location of interest for the output. In the main text, we present a simple mechanism that allows this steady state output level to adapt to changes in separation between the input location and the target location. This mechanism involves a diffusing inhibitor (same diffusivity and reverse reaction rate constant as the output signal) that is activated at the target location. While the computational results in the main text have been presented for closed nodes, this mechanism in fact relies on all the nodes being far from saturation. In this regime, the system essentially behaves as a system with open nodes, and therefore we first examine the limiting case of open nodes in our analysis below.

For a single closed node subject to localised regulation, the model is as follows:

For $\theta \in [0, \theta_1)$,

$$\frac{\partial [X^*]}{\partial t} = -k_2 X^* + D_{X^*} \frac{\partial^2 [X^*]}{\partial \theta^2}$$
$$\frac{\partial [X]}{\partial t} = k_2 X^* + D_X \frac{\partial^2 [X]}{\partial \theta^2}$$

For $\theta \in [\theta_1, \theta_2]$,

$$\frac{\partial [X^*]}{\partial t} = k_1 S X - k_2 X^* + D_{X^*} \frac{\partial^2 [X^*]}{\partial \theta^2}$$
$$\frac{\partial [X]}{\partial t} = -k_1 S X + k_2 X^* + D_X \frac{\partial^2 [X]}{\partial \theta^2}$$

For $\theta \in (\theta_2, 1]$,

$$\frac{\partial [X^*]}{\partial t} = -k_2 X^* + D_{X^*} \frac{\partial^2 [X^*]}{\partial \theta^2}$$
$$\frac{\partial [X]}{\partial t} = k_2 X^* + D_X \frac{\partial^2 [X]}{\partial \theta^2}$$

Throughout our study, we assume that for nodes where both forms are equally diffusible, the initial conditions are such that the total amount of modified forms is uniform across the whole domain. We make this assumption here. In this case, the model becomes:

$$\frac{\partial [X^*]}{\partial t} = -k_2 X^* + D_{X^*} \frac{\partial^2 [X^*]}{\partial \theta^2}$$

For $\theta \in [\theta_1, \theta_2]$,

$$\frac{\partial [X^*]}{\partial t} = k_1 S (X_T - X^*) - k_2 X^* + D_{X^*} \frac{\partial^2 [X^*]}{\partial \theta^2}$$

For $\theta \in (\theta_2, 1]$,

$$\frac{\partial [X^*]}{\partial t} = -k_2 X^* + D_{X^*} \frac{\partial^2 [X^*]}{\partial \theta^2}$$

When the input level is such that the X node is far from saturation everywhere in the domain (i.e. $X^* << X_T$), the kinetics in the input location $[\theta_1, \theta_2]$ is approximately described by a zeroth order production and a first order removal, i.e. $k_1 S X_T - k_2 X^*$. Thus the system is approximately equivalent to an 'open' node in this regime, and we examine this case below.

5.1 Open nodes with closed boundaries and uniform degradation

For a single 'open' node, with constant production of signal species localised over $\theta \in [\theta_1, \theta_2]$ in a 1-D domain of $L = 1$, with uniform degradation over the whole domain, the model is as follows:

For $\theta \in [0, \theta_1)$,

$$\frac{\partial[X^*]}{\partial t} = -k_2 X + D_{X^*}\frac{\partial^2[X^*]}{\partial\theta^2}$$

For $\theta \in [\theta_1, \theta_2]$,

$$\frac{\partial[X^*]}{\partial t} = k_1 S X_T - k_2 X + D_{X^*}\frac{\partial^2[X^*]}{\partial\theta^2}$$

For $\theta \in (\theta_2, 1]$,

$$\frac{\partial[X^*]}{\partial t} = -k_2 X + D_{X^*}\frac{\partial^2[X^*]}{\partial\theta^2}$$

With homogeneous Neumann (no flux) boundaries, the solution is given by:

For $\theta \in [0, \theta_1)$,

$$X = \frac{k_1 S X_T \cosh(\omega\theta)}{k_2 \sinh(\omega)}\left(\sinh(\omega(1-\theta_1)) - \sinh(\omega(1-\theta_2))\right) \tag{4}$$

For $\theta \in [\theta_1, \theta_2]$,

$$\begin{aligned} X = &\frac{k_1 S X_T \cosh(\omega\theta)}{k_2 \sinh(\omega)}\left(\sinh(\omega(1-\theta)) - \sinh(\omega(1-\theta_2))\right) \\ &+ \frac{k_1 S X_T \cosh(\omega(1-\theta))}{k_2 \sinh(\omega)}\left(\sinh(\omega\theta) - \sinh(\omega\theta_1)\right) \end{aligned} \tag{5}$$

For $\theta \in (\theta_2, 1]$,

$$X = \frac{k_1 S X_T \cosh(\omega(1-\theta))}{k_2 \sinh(\omega)}\left(\sinh(\omega\theta_2) - \sinh(\omega\theta_1)\right) \tag{6}$$

where $\omega^2 = \frac{k_2}{D_{X^*}}$, i.e. the ratio of the reverse reaction rate constant to the diffusivity.

Using the above expression, where $\theta \in [\theta_1, \theta_2]$ equal to $[p_1 - l/2, p_1 + l/2]$ and averaging over the output location $[p_2 - l/2, p_2 + l/2]$. this allows us to estimate the dependence of output at a target location in terms of the input. From this we see that. Now consider the case where we swap the put and target locations.

If we now consider two compartments, with constant production localised in one of them, the above solution can be used to see that the steady state level in compartment 1 (centred at p_1) is related to a production in compartment 2 (centred at p_2 ($p_1 < p_2$))in exactly the same way as the steady state level in compartment 2 is related to a production in compartment 1 (this assumes thin compartments).

Using (6) above, we find:

$$\begin{aligned} \langle X^* \rangle_2 &= \frac{4\sinh^2(\frac{\omega l}{2}) k_1 S X_T}{l k_2 \omega \sinh(\omega)} \cosh(\omega p_1)\cosh(\omega(1-p_2)) \\ &= \frac{4\sinh^2(\frac{\omega l}{2})}{l k_2 \omega \sinh(\omega)} \cosh(\omega p_1)\cosh(\omega(1-p_2))(\textit{Production rate of X in compartment } 1) \end{aligned} \tag{7}$$

Now consider the case where the production is in compartment 2. Here we use (4) above to calculate the average:

$$\begin{aligned} \langle X \rangle_1 &= \frac{4\sinh^2(\frac{\omega l}{2}) k_1 S X_T}{l k_2 \omega \sinh(\omega)} \cosh(\omega(1-p_2))\cosh(\omega p_1) \\ &= \frac{4\sinh^2(\frac{\omega l}{2})}{l k_2 \omega \sinh(\omega)} \cosh(\omega(1-p_2)\cosh(\omega p_1))(\textit{Production rate of X in compartment } 2) \end{aligned} \tag{8}$$

From (7) and (8), it is clear that the constant of proportionality multiplying the production rate is the same in both cases.

5.1.1 Adaptation to spatial separation at a fixed 'target' location

The above analysis reveals that for compartments of the same size, in a domain with closed boundaries and uniform degradation, the steady state level in one compartment is related to a production in a second compartment in exactly the same way as the steady state level in the second compartment is related to a production in the first. The above result can exploited to achieve adaptation to spatial separation in a compartment within a uniform channel. This can be seen as follows:

- Suppose X production is confined to compartment 1, and the goal is to achieve adaptation of average steady state X output in compartment 2 (the 'target' location) to changes in separation between the compartments. From (7), the average of X in compartment 2 is:

$$\langle X\rangle_2 = \frac{4\sinh^2(\frac{\omega l}{2})}{lk_2\omega\sinh(\omega)}\cosh(\omega p_1)\cosh(\omega(1-p_2))(\textit{Production rate of X in compartment } 1) \quad (9)$$

- Consider a second node Y, whose production is confined to compartment 2. If Y has the same ratio of diffusivity to reverse reaction rate constant as X, from (8), The average of Y in compartment 1 is:

$$\langle Y\rangle_1 = \frac{4\sinh^2(\frac{\omega l}{2})}{lk_{2Y}\omega\sinh(\omega)}\cosh(\omega(1-p_2)\cosh(\omega p_1))(\textit{Production rate of Y in compartment } 2) \quad (10)$$

- We see that, if Y regulates node X in such a way that the production rate of X in compartment 1 is inversely proportional to the average of Y in compartment 1, then the location dependent terms in (10) cancel, and as a result, $\langle X\rangle_2$ no longer depends on the location of either compartment in the domain.

- A possible mechanism for the type of regulation of X by Y described above is through a localised node directly regulating the production of X. For instance, suppose the production rate of X is regulated by a localised node Z (the production rate of X is proportional to the concentration of Z). We have

$$(\textit{Production rate of X in compartment } 1) = k_1 Z^* X_T \quad (11)$$

  The inverse proportionality of the production rate of X in compartment 1 to the average of Y in compartment 1 can be approximately realised if Y inhibits node Z: with the degradation rate of Z proportional to the level of Y. At steady state we have:

$$Z^* \approx \frac{k_z Z_T}{k_z + k_{dz}\langle Y\rangle_1} \quad (12)$$

  And for the Z node far from saturation,

$$Z^* \approx \frac{k_z Z_T}{k_{dz}\langle Y\rangle_1} \quad (13)$$

  This relies on relatively thin compartments, where the Y location in compartment is essentially uniform and is captured by its average. Thus, computing the production rate of X from (11), with Z^* from (13), and $\langle Y\rangle_1$ from (10), and inserting the resulting expression into (9), we see that the location dependence term in (9) is cancelled, thus allowing approximate adaptation of $\langle X\rangle_2$ to changes of separation:

$$\langle X\rangle_2 = \frac{4\sinh^2(\frac{\omega l}{2})}{lk_{dx}\omega\sinh(\omega)}\cosh(\omega p_1)\cosh(\omega(1-p_2))(k_1 Z^* X_T) \quad (14)$$

$$\approx \frac{k_1 X_T 4\sinh^2(\frac{\omega l}{2})}{lk_{dx}\omega\sinh(\omega)}\cosh(\omega p_1)\cosh(\omega(1-p_2))\frac{k_z Z_T}{k_{dz}\langle Y\rangle_1} \quad (15)$$

$$= \frac{k_1 X_T k_{dy}}{k_{dx}}\frac{k_z Z_T}{k_{dz}(\textit{Production rate of Y in compartment } 2)} \quad (16)$$

We note that while the above results for the compartmental averages are exact irrespective of the size of the compartments, the compartments are not well-mixed. Since the cancellation effect of inhibition involves a local rather than an average concentration, this can only be attained in the limit of thin compartments relative to domain size (so that there is no significant disparity between the local concentration and the compartmental average).

5.2 Adaptation to spatial separation: With closed nodes

The above mechanism for adaptation to spatial separation has been demonstrated for a system with open nodes. The same mechanism works for the systems with closed nodes (which are the focus of our study) if the kinetic parameters are such that all the nodes are far from saturation. In such cases, as discussed earlier, the behaviour of each node can be approximated by a an open system description, with a zeroth order 'production' reaction (production rate possibly proportional to a signal), and a first order 'degradation' reaction (degradation rate constant possibly proportional to a signal). In this regime the above analysis is completely valid for an analogous system of interactions between closed nodes X, Y and Z, and the steady state level of X output at the (fixed) target location can be seen to adapt to changes in the location of node Z. This is supported by the computational results for the system with closed nodes, presented in the main text.

5.3 Combined adaptation to both signal level and spatial separation

The above mechanism can be incorporated within an incoherent feedforward (IFF) structure to achieve combined adaptation of an output at a target location, to both input signal and separation. Computational results demonstrating such combined adaptation are presented in the main text. In terms of the example discussed above, this involves having the localised Z node and the localised input to Y both regulated by a common (global) input signal - so that we have an IFF structure with respect to the input signal, regulating the localised node Z. This structure allows localised Z output to adapt to changes in the input signal level, and consequently the X output profile (in turn regulated by Z) also adapts to the input signal (across the whole domain). For a fixed level of input signal, the basic mechanism of adaptation to spatial separation explained above (inhibition of Z by Y) allows X output at the target location to also adapt to changes in separation.

In terms of the analysis above, this can be seen as follows: At steady state, we have, for Z far from saturation,

$$Z^* \approx \frac{k_z Z_T S}{k_{dz}\langle Y\rangle_1} \tag{17}$$

where S is the input signal level. Since Y is also subject to localised activation by the same signal, $\langle Y\rangle_1$ is now given by:

$$\begin{aligned}\langle Y\rangle_1 &= \frac{4\sinh^2(\frac{\omega l}{2})}{lk_{dy}\omega\sinh(\omega)}\cosh(\omega(1-p_2)\cosh(\omega p_1))(Production\ rate\ of\ Y\ in\ compartment\ 2)\\ &= \frac{4\sinh^2(\frac{\omega l}{2})}{lk_{dy}\omega\sinh(\omega)}\cosh(\omega(1-p_2)\cosh(\omega p_1))(k_y Y_T S)\end{aligned} \tag{18}$$

Thus, $\langle Y\rangle_1$ is proportional to the signal, and from (17) this implies that Z^* adapts to the signal level. If we look at the expression for $\langle X\rangle_2$, we see that

$$\begin{aligned}\langle X\rangle_2 &= \frac{4\sinh^2(\frac{\omega l}{2})}{lk_{dx}\omega\sinh(\omega)}\cosh(\omega p_1)\cosh(\omega(1-p_2))(Production\ rate\ of\ X\ in\ compartment\ 1)\\ &= \frac{4\sinh^2(\frac{\omega l}{2})}{lk_{d}\omega\sinh(\omega)}\cosh(\omega p_1)\cosh(\omega(1-p_2))k_1 Z^* X_T\\ &\approx \frac{4\sinh^2(\frac{\omega l}{2})}{lk_{dx}\omega\sinh(\omega)}\cosh(\omega p_1)\cosh(\omega(1-p_2))k_1 X_T\frac{k_z Z_T S}{k_{dz}\langle Y\rangle_1}\end{aligned} \tag{19}$$

From (18), we see that both the input signal dependence and the spatial dependence is cancelled out in (19), so that $\langle X\rangle_2$ now adapts to both the input signal level and the spatial separation between the target location and the localised activation of X:

$$\langle X\rangle_2 \approx \frac{k_{dy}}{k_{dx}} k_1 X_T \frac{k_z Z_T}{k_{dz} k_y Y_T}$$

5.4 Localisation in Incoherent feedforward adaptation

Adaptation in an incoherent feedforward motif relies on two independent pathways transducing the same signal level and acting antagonistically on a single output node. In the main text we examine the effect of localising one of the feedforward pathways, and focus on the case of a global (highly diffusible) output node (Fig. 8(b)). While this system exhibits adaptation to a spatially uniform signal, we show that it does not adapt to a graded signal. If the output node is only weakly diffusible, then the output - even the spatial average of the output - fails to adapt even to a spatially uniform signal (see Supplementary Figure 8). In relation to this point - where the output node is only weakly diffusible - we make the following observation: if the output node is 'open' with a production and removal of the output species (instead of a conserved pool of modified forms), with the two feedfroward pathways regulating the production and removal respectively, the spatial average of the output can still adapt to a spatially uniform signal. This is because the spatial average is determined by the ratio of production to removal, so that the input dependence of production and removal can cancel each other out. However, adaptation is only exhibited by the average output and not by the output concentration at any particular location.

As discussed in section 3 above, in the spatially distributed context, localising the two antagonistic pathways at different locations, while combining localised and global elements in each of these pathways, yields a mechanism where a signal transduced locally at multiple locations is then combined at single node without introducing gradients in the output.

- An IFF motif realised in this way would be capable of adaptation to changes in level of a uniform signal - as both the antagonistic pathways are exposed to the same level of signal.
- In general, the motif would no longer adapt to changes in a spatially graded signal - each of the antagonistic pathways is exposed to a different level of signal.
- However, the graded nature of the input signal would not introduce gradients in the output - this is prevented by the global elements in the antagonistic pathways.

6 Supplementary Note 6: Modular construction of multifunctional motifs

In the main text we discuss how multifunctional circuits combining multiple feedback loops sharing common nodes can be combined in a modular way by exploiting localisation of certain nodes/interactions. In our example, we have a three node motif, with two of the nodes shared between a positive feedback loop and a negative feedback loop. In the text we present computational results demonstrating how essentially localising the positive feedback (by localising the interaction specific to this feedback loop) can allow the motif to exhibit both behaviours - both bistable and oscillatory regimes accessible by varying a single input signal/parameter.

In Supplementary Figure 6(A), we examine the interplay between the size of this localisation, and the diffusivity of the common nodes. For higher diffusivity of the common nodes, we require a smaller size of localisation for the positive feedback to allow the motif to exhibit both characteristics. While the diffusivity of the common nodes is a parameter that can take the system between a bistable only regime and a combined regime (oscillations and bistability), the size of the localised subdomain functions essentially as a tuning parameter that can take the system between a bistable only regime and an oscillator only regime. This demonstrates the added value of manipulating localisation realise particular outcomes. In Supplementary Figure 6(B), we examine an alternative spatial design for the motif, that can allow modular

behaviour - localising the positive feedback interaction, and the negative feedback node Z at two different locations: having the common nodes (X and Y) local/weakly diffusible, the two feedback loops are essentially separated. Each one exhibits its characteristic behaviour, and this can be combined downstream by a node that is regulated by X or Y.

6.1 Designing two node motifs: Localisation to circumvent kinetic constraints

- We consider a two node positive feedback (mutual activation) motif with a threshold in one of the interactions. For basal parameters the system exhibits no bistability.
- Exploiting localisation: we examine the effect of one node being localised while the other is global, as a way of realising bistability throughout the domain (hence the other node is global rather than local).
- We consider two alternate spatial designs, where one of the nodes is localised and the other global (Supplementary Figure 7).
- Design 1: node preceding threshold interaction is global. In this case, localisation of the other node does not have any advantageous effect in enabling bistability.
- Design 2: Localisation of the node preceding threshold interaction. In this case, through an appropriate size of localisation, localisation creates an elevation in concentration, enabling a threshold to be crossed, and facilitating bistability creation through the positive feedback circuit.
- Note that the total amount of species in the domain is maintained this process.
- This shows the impact of localisation in enabling bistability and this is discussed in more complex three node motifs in the main text.
- In general, we note that imposing localisation on a local node only helps if the resulting locally elevated total amount at this node is part of the threshold interaction. If this is not the case, and it is a downstream local node that participates in the threshold interaction, the local elevation at the upstream local node cannot enable a crossing of the threshold downstream. This is because the local total amount of the downstream node is the basic limiting factor in this case.

7 Supplementary Note 7: Spatial regulation of Polo Kinase

We examine two distinct intracellular contexts where experimental evidence shows that the localization of a protein, Polo kinase/Polo like kinase plays a central role. The first is in the transduction of cytoplasmic protein gradients that regulates polarization in the *C.elegans* zygote [10]. The second involves nuclear localization of Polo kinase triggering a mitotic switch, through a mechanism involving multiple possible feedback interactions [23]. Here we focus on a model suggested by experiments in *Drosophila* [15] - however, we note that nuclear localization of Polo and its role in the mitotic switch is observed across different organisms [23]. Both these examples highlight multiple themes encountered in the paper: the effect of localization, the presence of two diffusivity nodes, and consequences for inference as well as engineering design principles.

7.1 Transduction of cytoplasmic protein gradients by PLK1

A cascade of intracellular protein gradients is established during asymmetric cell division in the *C. elegans* zygote [10]. The RNA binding protein Mex-5 forms a gradient, with higher levels in the anterior cytoplasm. This upstream gradient drives gradient formation in the Polo like kinase PLK-1. The PLK-1 gradient in turn drives gradient formation in the RNA binding protein Pos-1. Experimental evidence and modelling suggests that these gradients are formed by spatially graded interconversion between slow and fast diffusing forms of these proteins in each case [33].

We build a model of this cascade using nodes having interconverting fast and slow diffusing forms in a 1-D spatial domain with no flux boundary conditions. This type of model has been used to describe an

individual step of this cascade and fit to experimental data [33]. We parametrise our model to capture the qualitative features of these gradients shown experimentally (see [10]). The structure of the model is as follows:

- Each step of the cascade - Mex5, PLK1, Pos1 is represented by a node with interconverting fast and slow diffusing forms.
- At each node, one of the interconversion reactions (fast to slow diffusing OR slow to fast diffusing) is mediated by an upstream node (posterior localised PAR1 in the case of Mex5), while the reverse reaction is assumed to have a fixed rate constant that is uniform across the domain.
- Posterior localised PAR1 mediates Mex5 conversion from slow to fast diffusing form
- The slow diffusing form of Mex5 mediates PLK1 conversion from fast to slow diffusing form
- The slow diffusing form of PLK1 mediates conversion of Pos1 from slow to fast diffusing form

The model equations are as follows, with * denoting the slow diffusing form:

$$
\begin{aligned}
\frac{\partial[Mex5^*]}{\partial t} &= k_{01}[Mex5] - k_{02}[Mex5^*] - k_{PAR1}[Mex5^*] + D_{Mex5^*}\frac{\partial^2[Mex5^*]}{\partial\theta^2} \\
\frac{\partial[Mex5]}{\partial t} &= -k_{01}[Mex5] + k_{02}[Mex5^*] + k_{PAR1}[Mex5^*] + D_{Mex5}\frac{\partial^2[Mex5]}{\partial\theta^2} \\
\frac{\partial[PLK1^*]}{\partial t} &= k_{03}[PLK1] - k_{04}[PLK1^*] + k_3[Mex5^*][PLK1] + D_{PLK1^*}\frac{\partial^2[PLK1^*]}{\partial\theta^2} \\
\frac{\partial[PLK1]}{\partial t} &= -k_{03}[PLK1] + k_{04}[PLK1^*] - k_3[Mex5^*][PLK1] + D_{PLK1}\frac{\partial^2[PLK1]}{\partial\theta^2} \\
\frac{\partial[Pos1^*]}{\partial t} &= k_{05}[Pos1] - k_{06}[Pos1^*] - k_6[PLK1^*][Pos1^*] + D_{Pos1^*}\frac{\partial^2[Pos1^*]}{\partial\theta^2} \\
\frac{\partial[Pos1]}{\partial t} &= -k_{05}[Pos1] + k_{06}[Pos1^*] + k_6[PLK1^*][Pos1^*] + D_{Pos1}\frac{\partial^2[Pos1]}{\partial\theta^2}
\end{aligned}
$$

The spatial domain is divided into two subdomains, representing the anterior and posterior cytoplasm. The rate constant k_{PAR1} is zero outside of the posterior compartment.

Note that in the above model the slow diffusing forms of these proteins are the active forms with respect to downstream regulation.

We also examine an alternative realization of the cascade, which reproduces the experimentally observed opposing relationship between the Mex5 and Pos1 gradients, while reversing the PLK1 gradient. In this case:

- The slow form of Mex5 mediates PLK1 conversion from slow to fast diffusing form
- The slow form of PLK1 mediates conversion of Pos1 from fast to slow diffusing form

Examining both realisations with a consistent relation between the input and output nodes is done for multiple reasons: (1) it allows us to elucidate basic design principles associated with gradient transduction across multistep cascades; (2) since Polo kinase is a focal point, it also examines different ways in which Polo kinase may be regulated and how it affects the overall outcome.

7.1.1 Results

We first examine the building block of the cascade - a single node with interconverting fast and slow diffusing forms. We consider the regime where the diffusivity of the fast diffusing form is high (relative to the kinetic rate constants), so that its profile is essentially uniform at steady state. There are two ways of localising an interconversion reaction to generate a gradient in the slow diffusing form and hence in the total amount - the first is by localising the conversion from fast to slow diffusing form, in which case the slow diffusing form accumulates in the subdomain where it is produced, forming a steady state profile where it is high in

this subdomain and low outside; the second is by localising the conversion from slow to fast diffusing form, in which case the slow diffusing form accumulates outside the subdomain where it is converted to the fast diffusing form, forming a steady state profile where it is low in this subdomain and high outside. In order to understand any differences in the effect of the localised interconversion in each case, we compare the two, starting from a system with spatially uniform basal interconversion rates, and increasing one of the localised interconversion rates starting from zero. We keep the sizes of the subdomains fixed. We find that as the rate of the localised interconversion increases:

- In each case, initially the local levels of both slow diffusing form and the total amount increase. With localised conversion of fast to slow diffusing forms in a particular subdomain, the levels increase in the localised subdomain, whereas in the other case they increase outside this subdomain.
- Further increase in the localised interconversion rate reveals a difference between the two gradient generating mechanisms - with localised conversion of fast to slow diffusing forms, the local levels of both slow diffusing form and the total amount exhibit a monotonic increase, whereas, with localised conversion of slow to fast diffusing forms, the local level of the slow diffusing form can eventually start decreasing, while the total amount exhibits a monotonic local increase.
- Thus, localised conversion from slow to fast diffusing form can result in a non-monotonic input response in the steady state concentration profile for the slow form while this does not happen with localised conversion from fast to slow diffusing form.

Next we use our understanding of a single stage to examine the gradient transduction cascade described above. As our focus in this section is on the role of Polo kinase localization, we examine two alternate realisations of the cascade that both give the same qualitative relationship between the Mex5 and Pos1 gradients - Mex5 is higher in the posterior cytoplasm and Pos1 is higher in the anterior cytoplasm. The gradient of PLK1 however is opposite in the two realisations. We can compare the two realisations in the context of the non-monotonic behaviour described above.

- In the experimentally observed scenario where Mex5 converts PLK1 from fast to slow diffusing form, having threshold behaviour in Pos1 regulation by PLK1 would not affect gradient formation, as the upstream signal strength is increased.
- In the alternative realisation, where Mex5 converts PLK1 from slow to fast form, having a threshold behaviour in Pos1 regulation by PLK1 can bring the above non-monotonic response into play and possibly abolish the Pos1 gradient as the upstream signal strength is increased. This because the local PLK1 level would eventually start to decrease as the upstream signal level increases.

7.2 Nuclear localization of Polo controlling mitotic entry

We now turn to a mathematical investigation of a proposed network governing mitotic entry in *Drosophila* with a focus on the role of Polo therein. The mathematical model is based a number of experiments which have been consolidated into a proposed network discussed in [15]. The goal of this work is to analyse and evaluate the proposed network model and reveal the underlying design principles. The model involves inactive Polo initially localised in the cytoplasm (sequestered by microtubule bound Map205), and being transferred to the nucleus upon activation. In the nucleus, Polo activates Cdc25 (initially localised in the nucleus). Cdc25 activation triggers its transport to the cytoplasm, where it activates the CycB-Cdk1 complex. This triggers localization of CycB-Cdk1 in the nucleus, where it can further enhance activation of Cdc25 by Polo. Thus, Cdc25 and CycB-Cdk1 form a spatially distributed positive feedback loop, which potentially allows a sharp, irreversible mitotic transition.

Interconversion between forms with different mobility has been used to study nuclear localization of CycB-Cdk1 in the context of a cell cycle switch [26]. We adopt a similar approach here, using nodes with interconverting fast and slow diffusing forms to represent the different spatially regulated components of this network, in a 1-D spatial domain with no-flux boundary conditions. Note that we do not explicitly describe the boundary of the domain as essentially the same results ensue.

The structure of the model is as follows:

- The 1-D spatial domain is divided into two subdomains, representing the cytoplasm and the nucleus
- Polo is converted from fast (active) to slow diffusing (inactive) form in the cytoplasm
- The slow diffusing (inactive) form of Polo can be sequestered by binding to Map205, which is localised in the cytoplasm
- The slow diffusing (inactive) form of Polo can be converted to the fast diffusing (active) form by an upstream signal, representing Aurora B
- Cdc25 is converted from fast (active) to slow (inactive) form in the nucleus
- Active Polo in the nucleus converts Cdc25 from slow to fast form
- CycB-Cdk1 can exist in three forms, a fast diffusing inactive form, a fast diffusing active form, and a slow diffusing active form
- Active, fast diffusing CycB-Cdk1 can be converted to active slow diffusing form in the nucleus. This conversion involves a positive feedback, as suggested by Santos et al.
- Active Cdc25 in the cytoplasm can convert inactive CycB-Cdk1 to the fast diffusing active form
- Active CycB-Cdk1 in the nucleus promotes Cdc25 activation by Polo

We use the following notation in the model equations:

- $Polo^*$ denotes the slow diffusing, inactive form of Polo
- $Polo^{bound}$ denotes the inactive form of Polo bound to Map205 (non-diffusible)
- $Polo$ denotes the fast diffusing active form of Polo
- $Cdc25^*$ denotes the slow diffusing inactive form of Cdc25
- $Cdc25$ denotes the fast diffusing active form of Cdc25
- $CycB-Cdk1^{**}$ denotes the slow diffusing active form of CycB-Cdk1
- $CycB-Cdk1^*$ denotes the fast diffusing active form of CycB-Cdk1
- $CycB-Cdk1$ denotes the fast diffusing inactive form of CycB-Cdk1

The model equations are as follows:

$$
\begin{aligned}
\frac{\partial[Polo^{bound}]}{\partial t} &= -k_{unbind}[Polo^{bound}] + k_{bind}[Polo^*]\left([Map205_{Total}] - [Polo^{bound}]\right) \\
&+ D_{Polo^{bound}}\frac{\partial^2[Polo^{bound}]}{\partial\theta^2} \\
\frac{\partial[Polo^*]}{\partial t} &= k_{unbind}[Polo^{bound}] - k_{bind}[Polo^*]\left([Map205_{Total}] - [Polo^{bound}]\right) \\
&+ k_{-Polo}[Polo] - k_{02}[Polo^*] - k_{AuroraB}[Polo^*] + D_{Polo^*}\frac{\partial^2[Polo^*]}{\partial\theta^2} \\
\frac{\partial[Polo]}{\partial t} &= -k_{-Polo}[Polo] + k_{02}[Polo^*] + k_{AuroraB}[Polo^*] + D_{Polo}\frac{\partial^2[Polo]}{\partial\theta^2} \\
\frac{\partial[Cdc25^*]}{\partial t} &= k_{03}[Cdc25] - k_{04}[Cdc25^*] - k_3[Polo][Cdc25^*] + D_{Cdc25^*}\frac{\partial^2[Cdc25^*]}{\partial\theta^2} \\
\frac{\partial[Cdc25]}{\partial t} &= -k_{03}[Cdc25] + k_{04}[Cdc25^*] + k_3[Polo][Cdc25^*] + D_{Cdc25}\frac{\partial^2[Cdc25]}{\partial\theta^2} \\
\frac{\partial[CycB-Cdk1^*]}{\partial t} &= k_{05}[CycB-Cdk1] - k_{06}[CycB-Cdk1^*] + k_5[Cdc25][CycB-Cdk1] \\
&+ k_{07}[CycB-Cdk1^{**}] - k_8[CycB-Cdk1^*] + D_{CycB-Cdk1^*}\frac{\partial^2[CycB-Cdk1^*]}{\partial\theta^2} \\
\frac{\partial[CycB-Cdk1^{**}]}{\partial t} &= -k_{07}[CycB-Cdk1^{**}] + k_8[CycB-Cdk1^*] + D_{CycB-Cdk1^{**}}\frac{\partial^2[CycB-Cdk1^{**}]}{\partial\theta^2} \\
\frac{\partial[CycB-Cdk1]}{\partial t} &= -k_{05}[CycB-Cdk1] + k_{06}[CycB-Cdk1^*] - k_5[Cdc25][CycB-Cdk1] \\
&+ D_{CycB-Cdk1}\frac{\partial^2[CycB-Cdk1]}{\partial\theta^2}
\end{aligned}
$$

The spatial domain is divided into two subdomains, representing the nucleus and cytoplasm. $D_{Polo^{bound}}$ is set to zero. Spatial constraints are imposed following kinetic rate constants:

- k_{bind} is zero in the nucleus
- $[Map205_{Total}]$ is zero in the nucleus
- k_{-Polo} is zero in the nucleus
- k_{03} is zero in the cytoplasm
- k_3 is a function of the total active CycB-Cdk1 in the nucleus: $k_3 = k_a + k_b([CycB-Cdk1^*] + [CycB-Cdk1^{**}])$. k_3 is zero in the cytoplasm.
- k_5 is zero in the nucleus
- k_8 is a function of the total active CycB-Cdk1 in the nucleus: $k_8 = \frac{k_p([CycB-Cdk1^*]+[CycB-Cdk1^{**}])^4}{K_p+([CycB-Cdk1^*]+[CycB-Cdk1^{**}])^4}$. This functional form, representing a positive feedback driving nuclear localisation of active CycB-Cdk1 has been used in [26]. k_8 is zero in the cytoplasm.

We also examine a possible negative feedback regulation of Polo by Optn proposed by [14] as follows:

- Optn is converted from fast (active) to slow (inactive) form in the cytoplasm
- Active Polo in the cytoplasm converts Optn slow (inactive) to fast (active) form in the cytoplasm
- Active Optn can convert fast (active) Polo to slow (inactive) form in the nucleus

When addressing deviant, step back to the abstract, make broader point, and then come back to right. Local change in nature of effect on Polo activity in the nucleus.

7.2.1 Results

We first demonstrate how our model can generate a Polo kinase driven switch-like spatial redistribution of Cdc25 and CycB-Cdk1 that qualitatively matches experimentally observed behaviour. We consider the activation of Polo by an upstream input - representing Aurora B - and the resulting activation by Polo of the positive feedback loop formed by Cdc25 and CycB-Cdk1. This positive feedback is capable of generating multiple spatially inhomogeneous steady states ("spatial bistability") (Fig.9(h)), and we examine the system in a parameter regime where such behaviour is possible. The two steady states correspond to: (1) high (inactive) Cdc25 in the nucleus (low outside), and high inactive CycB-Cdk1 across both nucleus and cytoplasm, and (2) high Cdc25 across both nucleus and cytoplasm, and high active CycB-Cdk1 in the nucleus. Increasing the upstream (Aurora B) signal level can increase the level of active Polo in the nucleus and consequently trigger a switch from steady state (1) to steady state (2).

We have shown how a feedback loop can exhibit deviant behaviour depending on the localisation/spatial organisation of the interactions between nodes (see discussion above). This includes the possibility of a negative feedback loop exhibiting characteristics associated with a positive feedback loop or vice versa. We now examine how/whether such deviant behaviour is possible in the Cdc25/CycB-Cdk1 loop considered here. The spatial organisation of the interactions in the proposed realisation (informed by experiments [15]) ensures that it behaves as a bona fide positive feedback loop. However, we note that the nature of the feedback may be reversed if active CycB-Cdk1 accumulated in the cytoplasm instead of the nucleus (In the model this amounts to having the conversion of CycB-Cdk1 to the slow diffusing active form happening in the cytoplasm instead of the nucleus). This could happen for instance if the relevant enzyme was active in the cytoplasm instead of the nucleus. This would mean that any increase in active Cdc25 levels promotes accumulation of active CycB-Cdk1 in the cytoplasm (where it has no effect on Cdc25 activation) and a consequent drop in active CycB-Cdk1 in the nucleus (where it promotes activation of Cdc25). We analyse this possibility below, using the approach demonstrated in section 4 above.

[14] suggest that Polo activity in the nucleus may also be subject to spatially distributed negative feedback regulation involving the protein Optn. We incorporate this feedback as an augmentation to the above model the model and examine how this regulation can affect the Polo driven switch-like redistribution generated by the positive feedback loop. The proposed mechanism involves Optn activation by Polo in the cytoplasm, with Polo inactivation by Optn in the nucleus. Once more we note that, while it is possible for a negative feedback loop with localised interactions to exhibit deviant behaviour (thereby exhibiting the characteristics of a positive feedback loop), the spatial organisation here ensures that this does not happen. Consistent with the negative character of the feedback, it has been proposed that it functions to antagonize Polo activity in the nucleus. However, we find that while the nature of the feedback is not affected by the localisation of interactions, the local effect of the feedback at a particular location in the domain can exhibit anomalous behaviour. In the case of the negative feedback loop involving Polo and Optn, this can happen if instead of having an inactive (slow diffusing) and active (fast diffusing) form of Polo, we had instead a less active (slow diffusing) and more active (fast diffusing) form, with Optn converting the more active to the less active form. In this scenario, the feedback still behaves as a negative feedback (as can be demonstrated by the type of analysis shown above), but the feedback can increase the total level of Polo activity in the nucleus (relative to the case with no feedback) - see Fig. 9(k) in the main text and the discussion in supplementary section 4.5. Therefore, while the character of the feedback is negative, depending on parameters its function may in fact be to promote Polo activity in the nucleus rather than to antagonize it. Since Polo activity in the nucleus is of importance to multiple process, the possibility of location dependent anomalous effects could be particularly significant.

7.2.2 Possibility of deviant behaviour in the Cdc25 - CycB-Cdk1 feedback loop

For the purpose of this analysis, we assume equal diffusivity (high) of both forms of Cdc25 - this does not affect the conclusions, as it is the fast diffusing form of Cdc25 that activates CycB-Cdk1 in our model. We also ignore the fast diffusing active form of CycB-Cdk1 - this again is consistent with the slow diffusing active form (experimentally observed to be localised to the nucleus) being mainly responsible for enabling Polo activation of Cdc25. Thus we have an active and inactive form (equally, highly diffusible) for Cdc25 and a slow diffusing active form and fast diffusing inactive form for CycB-Cdk1. For simplicity of notation

in the analysis below, we use Y and Y^* to represent the inactive and active forms of Cdc25 respectively, and X and X^* to represent the inactive and active forms of CycB-Cdk1 respectively.

We consider the limit where the slow-diffusing active form of CyB-Cdk1, X^* is non-diffusible, and the inactive form X is highly diffusible.

In this limit, at steady state, X, Y, and Y^* are uniform across the whole domain, while X^* has a discontinuous profile as described previously. As before, at steady state, let x denote the spatially uniform concentration of the inactive form, x^*_{in} and x^*_{out} denote the spatially uniform concentrations of the active form inside and outside the nucleus (i.e. in the cytoplasm) respectively. Let y^* denote the spatially uniform concentration of Y^* across the domain. Let L denote the size of the whole spatial domain and w denote the size of the subdomain representing the nucleus. Let the total amount in the pool of modified forms for species X and Y be x_T and y_T.

For the scenario where CycB-Cdk1 is converted to the slow diffusing form (driven by active Cdc25) in the nucleus, i.e. the experimentally observed scenario, we have the following relationships at steady state. We use the same notations as before for the rate constants of the reaction. Within the nucleus:

$$k_{01}x - k_{02}x^*_{in} + k_2 h(y^*)x = 0$$
$$k_y g(x^*_{in})(y_T - y^*) - k_{dy}y^* = 0$$

In the cytoplasm:

$$k_{01}x - k_{02}x^*_{out} = 0$$

Here, h and g are Hill functions.

The fixed total amount imposes the following constraint:

$$Lx + wx^*_{in} + (L-w)x^*_{out} = x_T$$

Using these we can derive steady state relationships between x^*_{in} and y^* as before:

$$y^* = \frac{k_y g(x^*_{in}) y_T}{k_y g(x^*_{in}) + k_{dy}}$$

and

$$x^*_{in} = \frac{x_T}{\left(\frac{Lk_{02}}{k_{01}+k_2 h(y^*)} + w + \frac{(L-w)k_{01}}{k_{01}+k_2 h(y^*)}\right)}$$

These curves can be seen as analogous to the nullclines of a two node feedback system, and in this case, they are capable of exhibiting multiple points of intersection corresponding to multiple steady states - Fig. 9(l). This is consistent with the bistable behaviour of this feedback loop demonstrated for the full system (Fig. 9(h)).

If we examine the case where CycB-Cdk1 is converted to the slow diffusing form (driven by active Cdc25) in the cytoplasm, potentially through mislocalization of an enzyme, we have the following relationships at steady state. We use the same notations as before for the rate constants of the reaction. Within the nucleus:

$$k_{01}x - k_{02}x^*_{in} = 0$$
$$k_y g(x^*_{in})(y_T - y^*) - k_{dy}y^* = 0$$

In the cytoplasm:

$$k_{01}x - k_{02}x^*_{out} + k_2 h(y^*)x = 0$$

Here, h and g are Hill functions.

The fixed total amount imposes the following constraint:

$$Lx + wx^*_{in} + (L - w)x^*_{out} = x_T$$

Using these we can derive steady state relationships between x^*_{out} and y^* as before:

$$y^* = \frac{k_y g(x^*_{in}) y_T}{k_y g(x^*_{in}) + k_{dy}}$$

where x^*_{in} can be expressed in terms of x^*_{out} as

$$x^*_{in} = \frac{x_T - (L - w)x^*_{out}}{L\frac{k_{02}}{k_{01}} + w}$$

and

$$x^*_{out} = \frac{x_T}{\left(\frac{Lk_{02}}{k_{01}+k_2h(y^*)} + (L - w) + \frac{wk_{01}}{k_{01}+k_2h(y^*)}\right)}$$

We see that the curves defined by these relationships behave like the nullclines of a two node negative feedback loop, and are not capable of producing multiple intersections - Fig. 9(l).

References

[1] Adamala, K. P., Martin-Alarcon, D. A., Guthrie-Honea, K. R. and Boyden, E. S. [2017], 'Engineering genetic circuit interactions within and between synthetic minimal cells', *Nature Chemistry* **9**(5), 431–439.
URL: *https://doi.org/10.1038/nchem.2644*

[2] Atay, O. and Skotheim, J. M. [2017], 'Spatial and temporal signal processing and decision making by mapk pathways', *The Journal of Cell Biology* **216**(2), 317–330.
URL: *https://doi.org/10.1083/jcb.201609124*

[3] Booth, R., Qiao, Y., Li, M. and Mann, S. [2019], 'Spatial positioning and chemical coupling in coacervate-in-proteinosome protocells', *Angewandte Chemie International Edition* **58**(27), 9120–9124.
URL: *https://doi.org/10.1002/anie.201903756*

[4] Bugrim, A. E. [1999], 'Regulation of ca2+release by camp-dependent protein kinase a mechanism for agonist-specific calcium signaling?', *Cell Calcium* **25**(3), 219–226.
URL: *http://www.sciencedirect.com/science/article/pii/S0143416099900271*

[5] Carmona-Fontaine, C., Deforet, M., Akkari, L., Thompson, C. B., Joyce, J. A. and Xavier, J. B. [2017], 'Metabolic origins of spatial organization in the tumor microenvironment', *Proceedings of the National Academy of Sciences* **114**(11), 2934.
URL: *http://www.pnas.org/content/114/11/2934.abstract*

[6] Doncic, A., Atay, O., Valk, E., Grande, A., Bush, A., Vasen, G., Colman-Lerner, A., Loog, M. and Skotheim, J. M. [2015], 'Compartmentalization of a bistable switch enables memory to cross a feedback-driven transition', *Cell* **160**(6), 1182–1195.
URL: *http://www.sciencedirect.com/science/article/pii/S0092867415001981*

[7] Elani, Y., Trantidou, T., Wylie, D., Dekker, L., Polizzi, K., Law, R. V. and Ces, O. [2018], 'Constructing vesicle-based artificial cells with embedded living cells as organelle-like modules', *Scientific Reports* **8**(1), 4564.
URL: *https://doi.org/10.1038/s41598-018-22263-3*

[8] Gilmour, D., Rembold, M. and Leptin, M. [2017], 'From morphogen to morphogenesis and back', *Nature* **541**(7637), 311–320.
URL: *https://doi.org/10.1038/nature21348*

[9] Haeusler, R. A., McGraw, T. E. and Accili, D. [2018], 'Biochemical and cellular properties of insulin receptor signalling', *Nature Reviews Molecular Cell Biology* **19**(1), 31–44.
URL: *https://doi.org/10.1038/nrm.2017.89*

[10] Han, B., Antkowiak, K. R., Fan, X., Rutigliano, M., Ryder, S. P. and Griffin, E. E. [2018], 'Polo-like kinase couples cytoplasmic protein gradients in the *C. elegans* zygote', *Current Biology* **28**(1), 60–69.e8.
URL: *http://www.sciencedirect.com/science/article/pii/S0960982217315300*

[11] Hegemann, B., Unger, M., Lee, S. S., Stoffel-Studer, I., van den Heuvel, J., Pelet, S., Koeppl, H. and Peter, M. [2015], 'A cellular system for spatial signal decoding in chemical gradients', *Developmental Cell* **35**(4), 458–470.
URL: *http://www.sciencedirect.com/science/article/pii/S1534580715006607*

[12] Jimenez, A., Cotterell, J., Munteanu, A. and Sharpe, J. [2017], 'A spectrum of modularity in multi-functional gene circuits', *Molecular Systems Biology* **13**(4), 925.
URL: *https://www.embopress.org/doi/abs/10.15252/msb.20167347*

[13] Joesaar, A., Yang, S., Bögels, B., van der Linden, A., Pieters, P., Kumar, B. V. V. S. P., Dalchau, N., Phillips, A., Mann, S. and de Greef, T. F. A. [2019], 'DNA-based communication in populations of synthetic protocells', *Nature Nanotechnology* **14**(4), 369–378.
URL: *https://doi.org/10.1038/s41565-019-0399-9*

[14] Kachaner, D., Filipe, J., Laplantine, E., Bauch, A., Bennett, K. L., Superti-Furga, G., Israël, A. and Weil, R. [2012], 'Plk1-dependent phosphorylation of optineurin provides a negative feedback mechanism for mitotic progression', *Molecular Cell* **45**(4), 553–566.
URL: *https://doi.org/10.1016/j.molcel.2011.12.030*

[15] Kachaner, D., Garrido, D., Mehsen, H., Normandin, K., Lavoie, H. and Archambault, V. [2017], 'Coupling of Polo kinase activation to nuclear localization by a bifunctional NLS is required during mitotic entry', *Nature Communications* **8**(1), 1701.
URL: *https://doi.org/10.1038/s41467-017-01876-8*

[16] Kiekebusch, D. and Thanbichler, M. [2014], 'Spatiotemporal organization of microbial cells by protein concentration gradients', *Trends in Microbiology* **22**(2), 65–73.
URL: *http://www.sciencedirect.com/science/article/pii/S0966842X1300228X*

[17] Krishnan, J., Lu, L. and Alam Nazki, A. [2020], 'The interplay of spatial organization and biochemistry in building blocks of cellular signalling pathways', *Journal of The Royal Society Interface* **17**(166), 20200251.
URL: *https://royalsocietypublishing.org/doi/abs/10.1098/rsif.2020.0251*

[18] Li, L., Klim, J. R., Derda, R., Courtney, A. H. and Kiessling, L. L. [2011], 'Spatial control of cell fate using synthetic surfaces to potentiate TGF-beta signaling', *Proceedings of the National Academy of Sciences* **108**(29), 11745.
URL: *http://www.pnas.org/content/108/29/11745.abstract*

[19] Menon, G., Okeke, C. and Krishnan, J. [2017], 'Modelling compartmentalization towards elucidation and engineering of spatial organization in biochemical pathways', *Scientific Reports* **7**(1), 12057.
URL: *https://doi.org/10.1038/s41598-017-11081-8*

[20] Munoz-Garcia, J. and Ares, S. [2016], 'Formation and maintenance of nitrogen-fixing cell patterns in filamentous cyanobacteria', *Proceedings of the National Academy of Sciences* **113**(22), 6218.
URL: *http://www.pnas.org/content/113/22/6218.abstract*

[21] Nadell, C. D., Drescher, K. and Foster, K. R. [2016], 'Spatial structure, cooperation and competition in biofilms', *Nature Reviews Microbiology* **14**(9), 589–600.
URL: *https://doi.org/10.1038/nrmicro.2016.84*

[22] Olson, S. D., Fauci, L. J. and Suarez, S. S. [2011], 'Mathematical modeling of calcium signaling during sperm hyperactivation', *Molecular Human Reproduction* **17**(8), 500–510.
URL: *https://doi.org/10.1093/molehr/gar040*

[23] Pintard, L. and Archambault, V. [2018], 'A unified view of spatio-temporal control of mitotic entry: Polo kinase as the key', *Open Biology* **8**(8), 180114.
URL: *https://royalsocietypublishing.org/doi/abs/10.1098/rsob.180114*

[24] Rai, A. K., Chen, J.-X., Selbach, M. and Pelkmans, L. [2018], 'Kinase-controlled phase transition of membraneless organelles in mitosis', *Nature* **559**(7713), 211–216.
URL: *https://doi.org/10.1038/s41586-018-0279-8*

[25] Robbins, J. R., Monack, D., McCallum, S. J., Vegas, A., Pham, E., Goldberg, M. B. and Theriot, J. A. [2001], 'The making of a gradient: Icsa (virg) polarity in shigella flexneri', *Molecular Microbiology* **41**(4), 861–872.
URL: *https://doi.org/10.1046/j.1365-2958.2001.02552.x*

[26] Santos, S. D. M., Wollman, R., Meyer, T. and Ferrell, J. E. [2012], 'Spatial positive feedback at the onset of mitosis', *Cell* **149**(7), 1500–1513.
URL: *http://www.sciencedirect.com/science/article/pii/S0092867412006496*

[27] Schuster, B. S., Reed, E. H., Parthasarathy, R., Jahnke, C. N., Caldwell, R. M., Bermudez, J. G., Ramage, H., Good, M. C. and Hammer, D. A. [2018], 'Controllable protein phase separation and modular recruitment to form responsive membraneless organelles', *Nature Communications* **9**(1), 2985.
URL: *https://doi.org/10.1038/s41467-018-05403-1*

[28] Semenov, S. N., Markvoort, A. J., de Greef, T. F. A. and Huck, W. T. S. [2014], 'Threshold sensing through a synthetic enzymatic reaction–diffusion network', *Angewandte Chemie International Edition* **53**(31), 8066–8069.
URL: *https://doi.org/10.1002/anie.201402327*

[29] Semenov, S. N., Markvoort, A. J., Gevers, W. B. L., Piruska, A., de Greef, T. F. A. and Huck, W. T. S. [2013], 'Ultrasensitivity by molecular titration in spatially propagating enzymatic reactions', *Biophysical Journal* **105**(4), 1057–1066.
URL: *http://www.sciencedirect.com/science/article/pii/S0006349513007819*

[30] Shin, Y. and Brangwynne, C. P. [2017], 'Liquid phase condensation in cell physiology and disease', *Science* **357**(6357), eaaf4382.
URL: *http://science.sciencemag.org/content/357/6357/eaaf4382.abstract*

[31] Sturrock, M., Terry, A. J., Xirodimas, D. P., Thompson, A. M. and Chaplain, M. A. [2011], 'Spatio-temporal modelling of the Hes1 and p53-Mdm2 intracellular signalling pathways', *Journal of Theoretical Biology* **273**(1), 15 – 31.
URL: *http://www.sciencedirect.com/science/article/pii/S0022519310006715*

[32] Wagner, H. J., Engesser, R., Ermes, K., Geraths, C., Timmer, J. and Weber, W. [2019], 'Synthetic biology-inspired design of signal-amplifying materials systems', *Materials Today* **22**, 25–34.
URL: *http://www.sciencedirect.com/science/article/pii/S1369702118300622*

[33] Wu, Y., Han, B., Li, Y., Munro, E., Odde, D. J. and Griffin, E. E. [2018], 'Rapid diffusion-state switching underlies stable cytoplasmic gradients in the *Caenorhabditis elegans* zygote', *Proceedings of the National Academy of Sciences* **115**(36), E8440.
URL: *http://www.pnas.org/content/115/36/E8440.abstract*

[34] Wu, Y., Zhang, H. and Griffin, E. E. [2015], 'Coupling between cytoplasmic concentration gradients through local control of protein mobility in the *Caenorhabditis elegans* zygote', *Molecular Biology of the Cell* **26**(17), 2963–2970.
URL: *https://doi.org/10.1091/mbc.E15-05-0302*

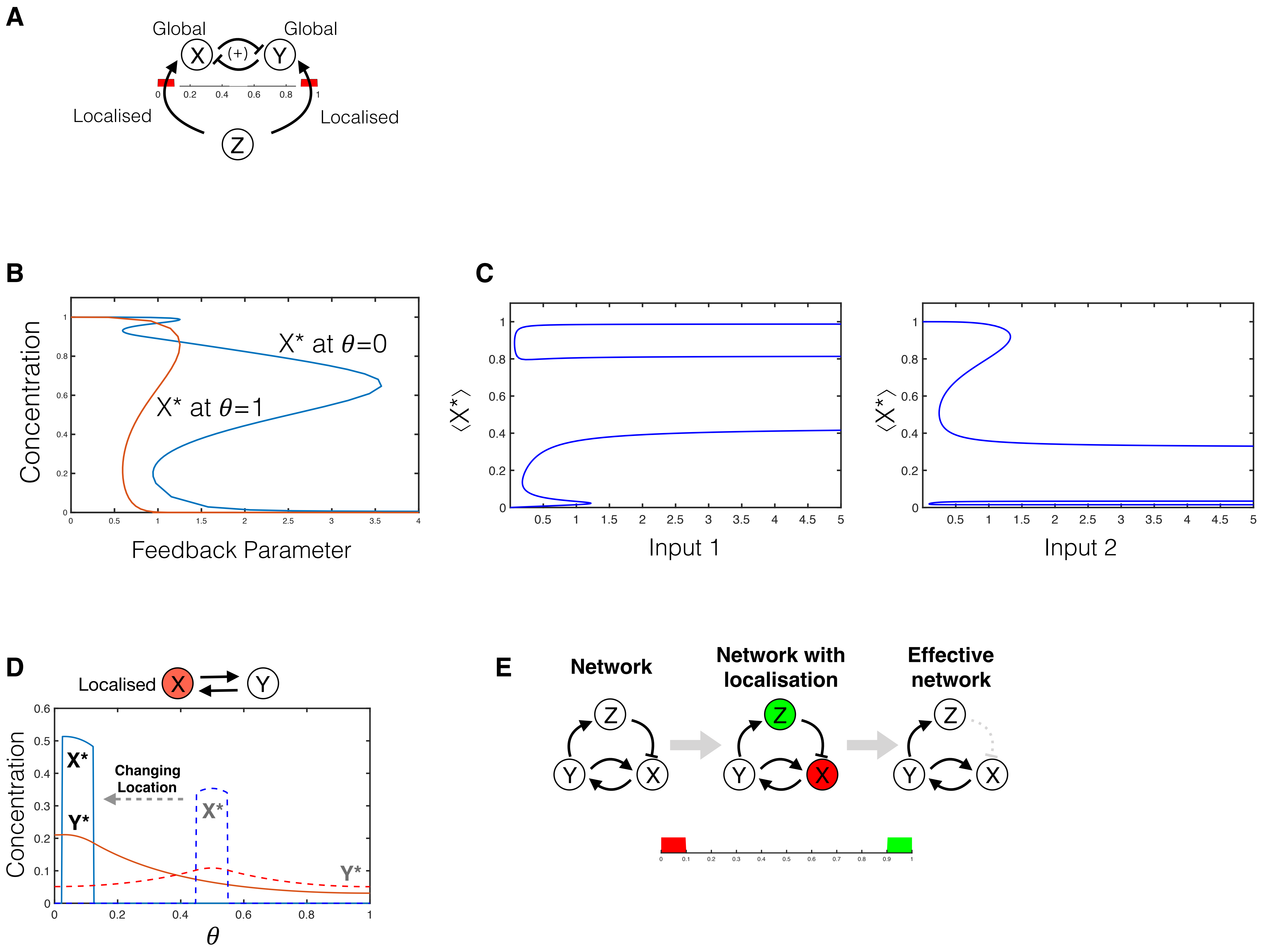

Supplementary Figure 1: Basic effects of localisation.
(A) Schematic of the motif indicating localised interactions. (B) Comparison of the bifurcation characteristics in terms of steady state X* concentration at the centre of the domain, and at the boundary. X* levels at the boundary do not reflect the tri-stable behaviour of the system, and in fact resembles the behaviour of the well-mixed (ODE) realisation of the motif. (C) Bifurcation analysis indicates that, a uniform input signal cannot switch the system between the X* low and X* high steady states, rather it can only switch between either the low and 'mixed' steady state, or between the high and 'mixed' steady state. (D) With a localised node in a feedback loop containing a moderately diffusible node, the basic location dependent behaviour seen in Fig.2 (C) and Fig.2 (D) is also reflected in the output level of the localised node X (contrast with Fig.2 (C)). (E) Example of a network where localisation of two nodes at different locations alters the network structure by preventing an interaction.

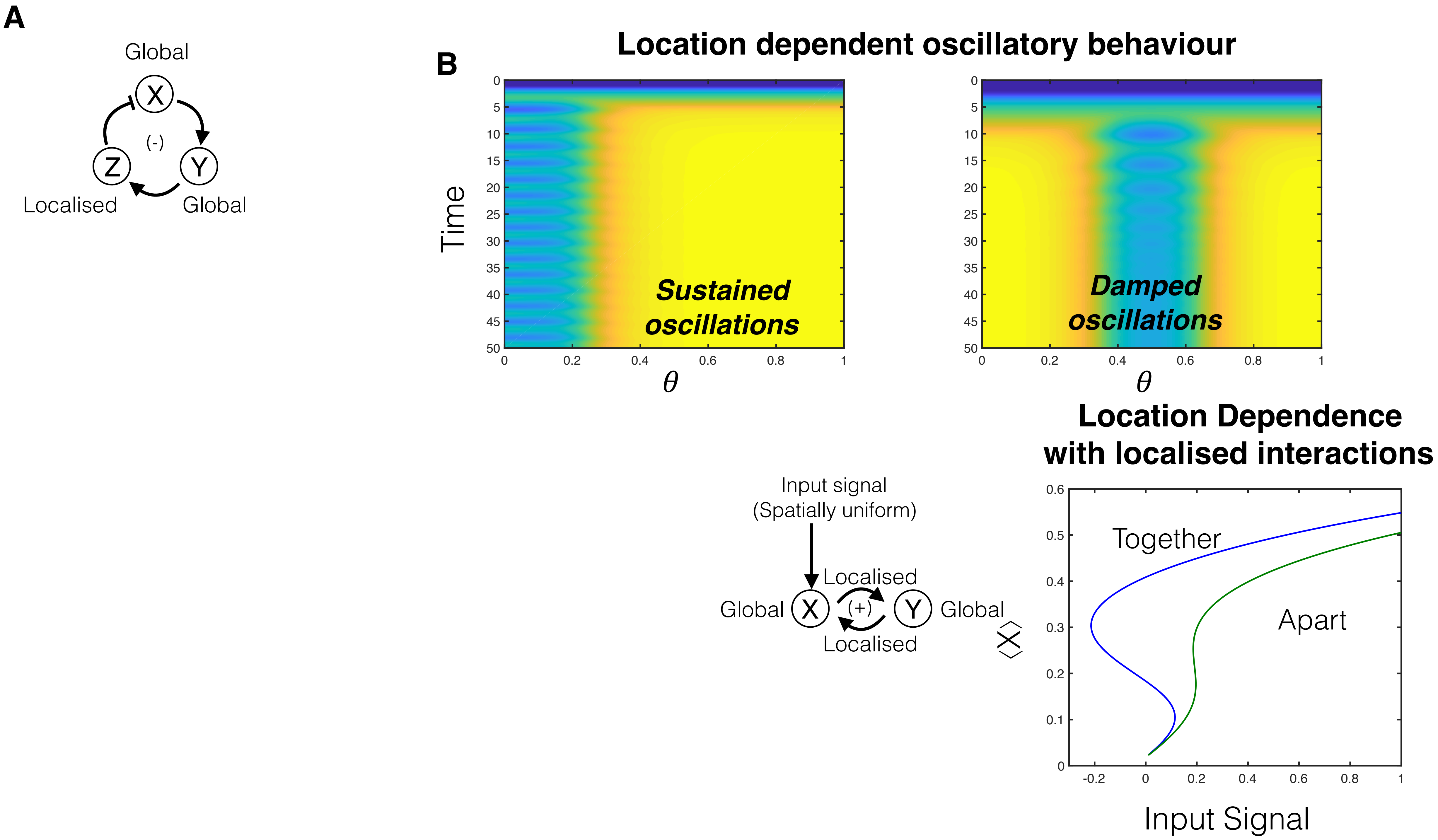

Supplementary Figure 2: Localisation introduces location dependence into dynamical characteristics of a motif.
(A) Schematic of a three node negative feedback motif capable of oscillatory behaviour, with one node localised and others diffusible. (B) Location dependent oscillations. Sustained oscillations possible with node Z localised at boundary, damped oscillations when Z localised at centre of domain. (C) Two node positive feedback motif with interactions localised, and (moderately) diffusible nodes. Co-localisation of interactions favours bistability, localising apart can attenuate bistable characteristics.

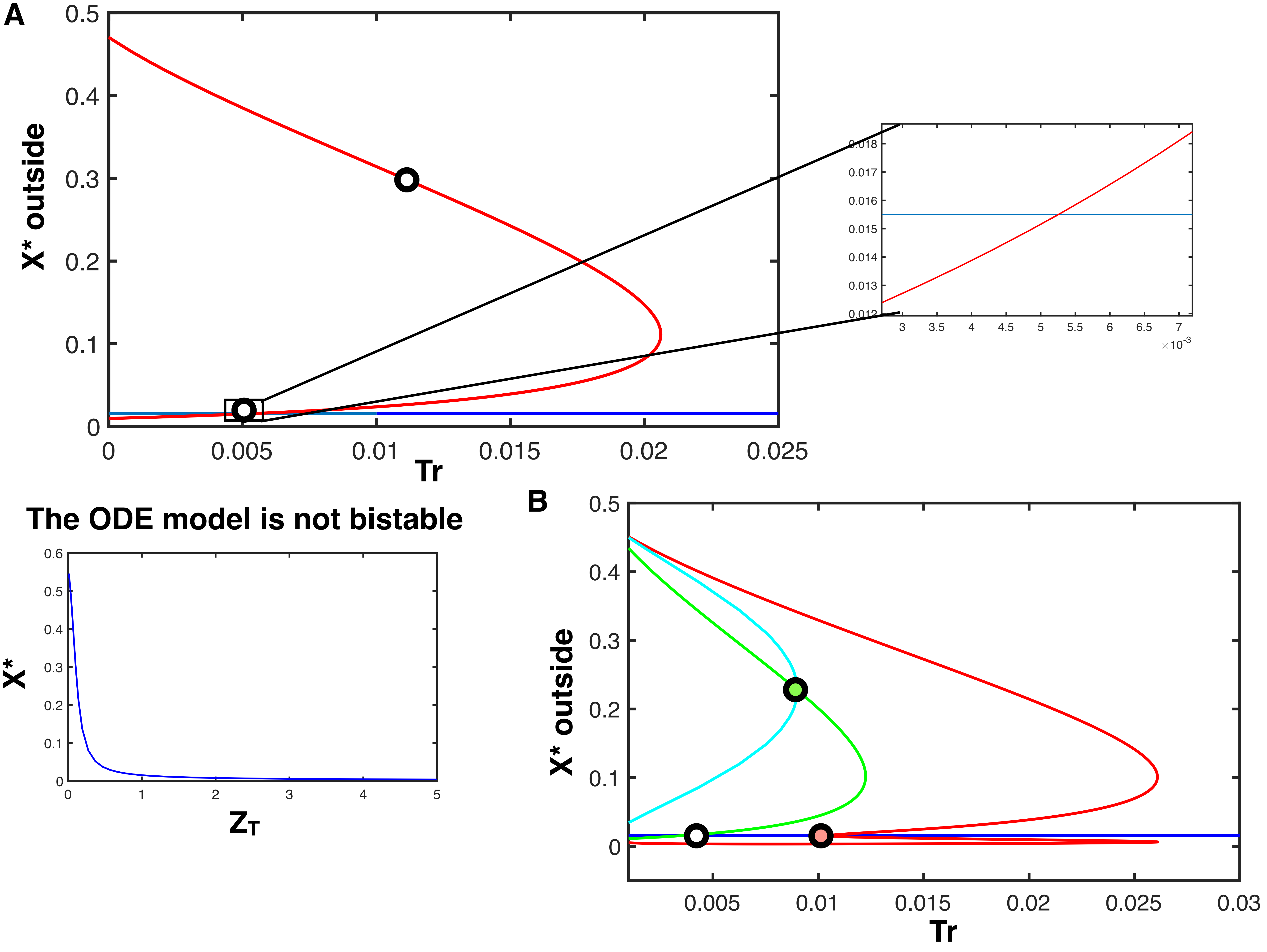

Supplementary Figure 3: Bifurcation analysis of localisation induced instability using compartmental ODE models.
See discussion in Supplementary Note 2 for details.

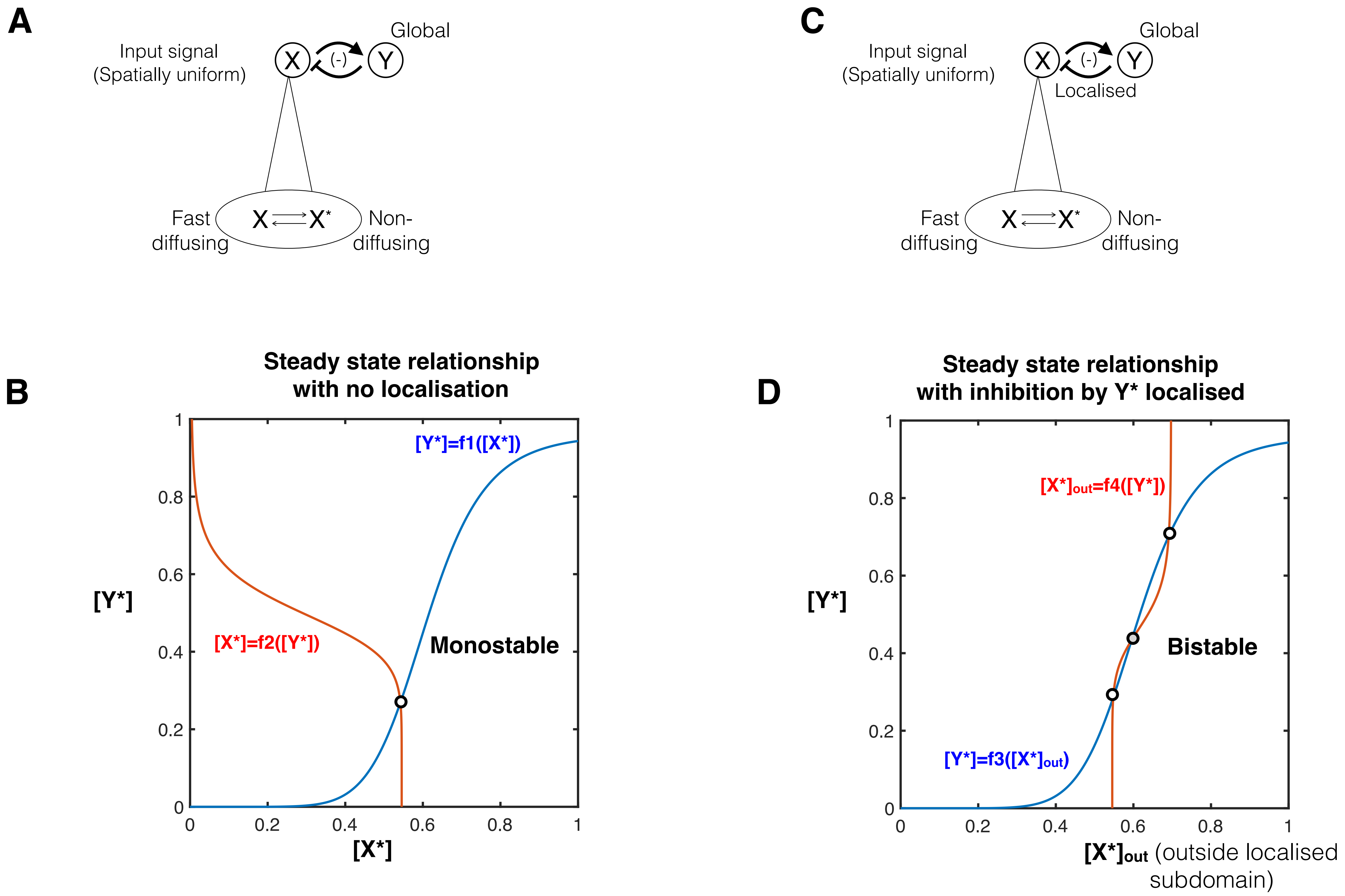

Supplementary Figure 4: Demonstration of how a two node negative feedback circuit with a two-diffusivity node can acquire positive feedback (bistable) characteristics when one of the interactions is localised.
See discussion in Supplementary Note 4.5 for details.

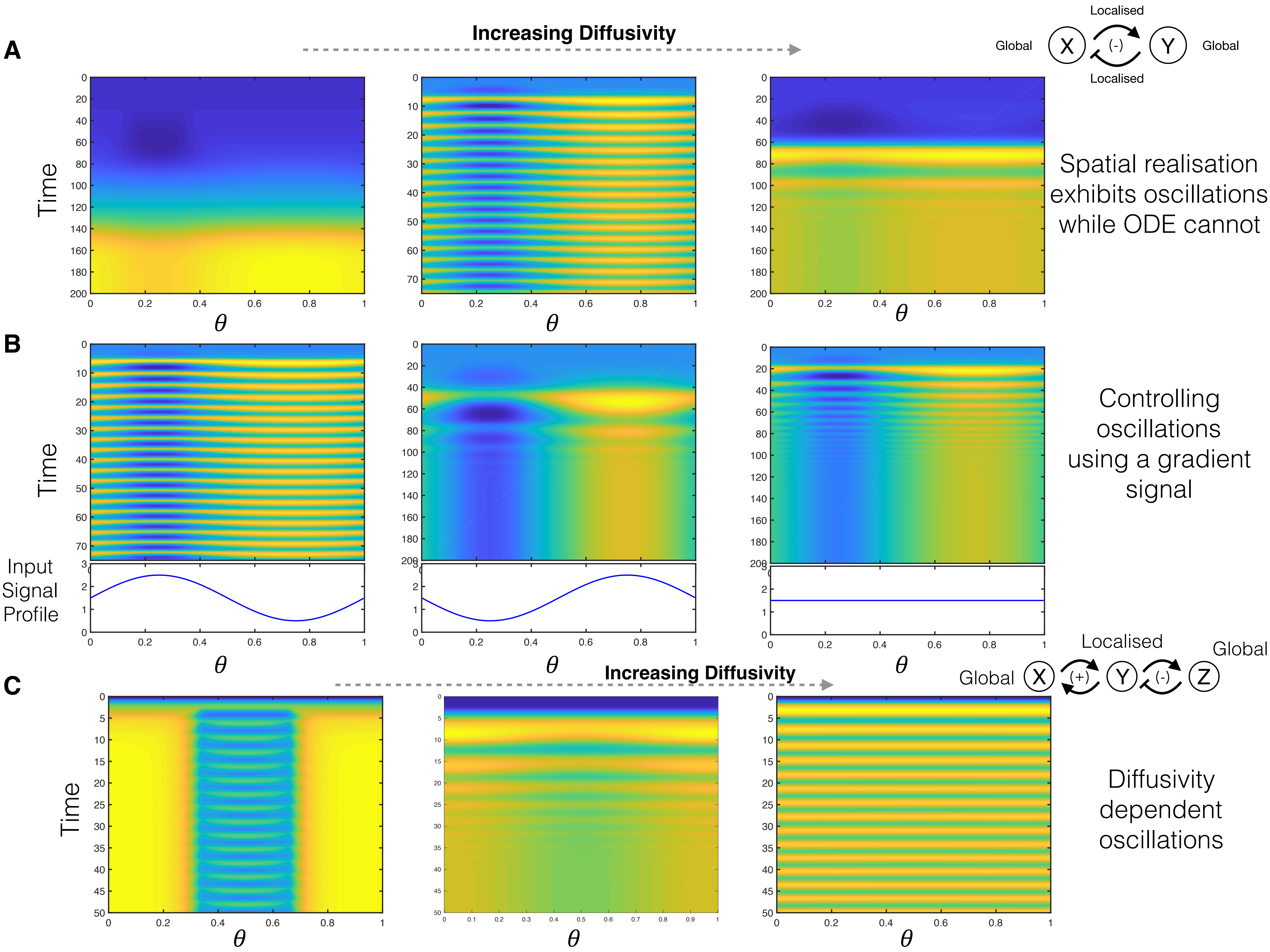

Supplementary Figure 5: Basic effects of localisation on motifs with oscillatory characteristics.

(A) A two-node negative feedback motif with diffusible nodes and interactions localised apart is capable of sustained spatio-temporal oscillations, while the well-mixed (ODE) realisation cannot exhibit limit cycle oscillations. (B) The parameters of this system can be tuned such that sustained oscillations can only be triggered by a graded signal with a certain phase and minimum amplitude. (C) Diffusivity dependent oscillatory behaviour in an activator inhibitor type oscillator motif with a localised node. Sustained oscillations at both very low and very high diffusivities, while this behaviour is lost at intermediate diffusivities.

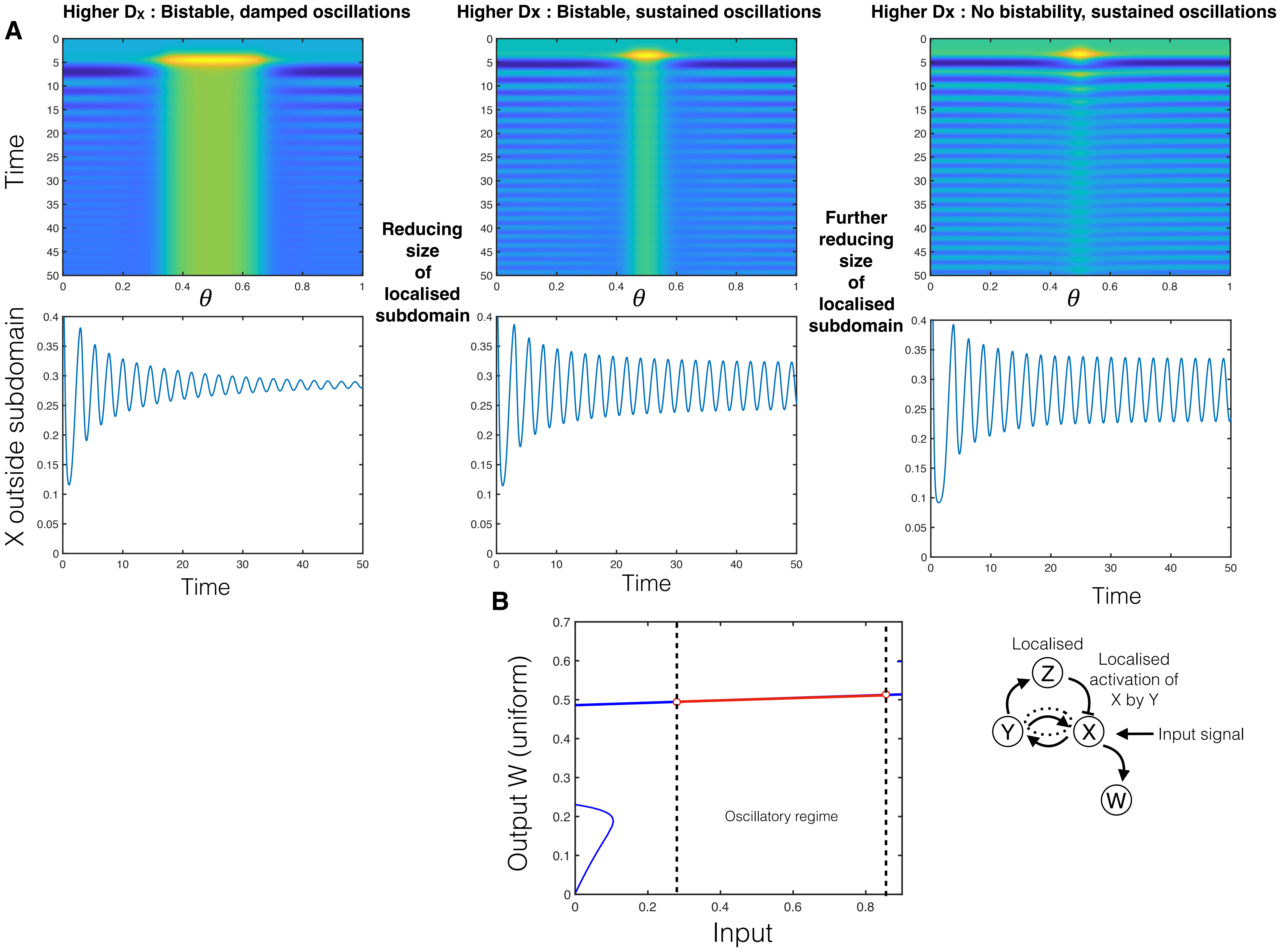

Supplementary Figure 6: Modular construction of multifunctional motifs.
(A) Interplay between diffusivity of a common node and size of localised subdomain. With higher diffusivity of node X (higher that for the case presented in the main text), the positive feedback loop dominates, and sustained oscillations are prevented. Oscillatory behaviour is recovered by reducing the size of the localised subdomain (where the positive feedback interaction is localised. However, even further reduction in size causes the negative feedback behaviour to dominate, and the system ceases to exhibit bistability, while retaining oscillatory behaviour. (B) With the two feedback loops localised at different locations and their outputs combined by a global downstream elements, the input thresholds for each type of behaviour is exactly the same as for the two individual feedbacks in isolation.

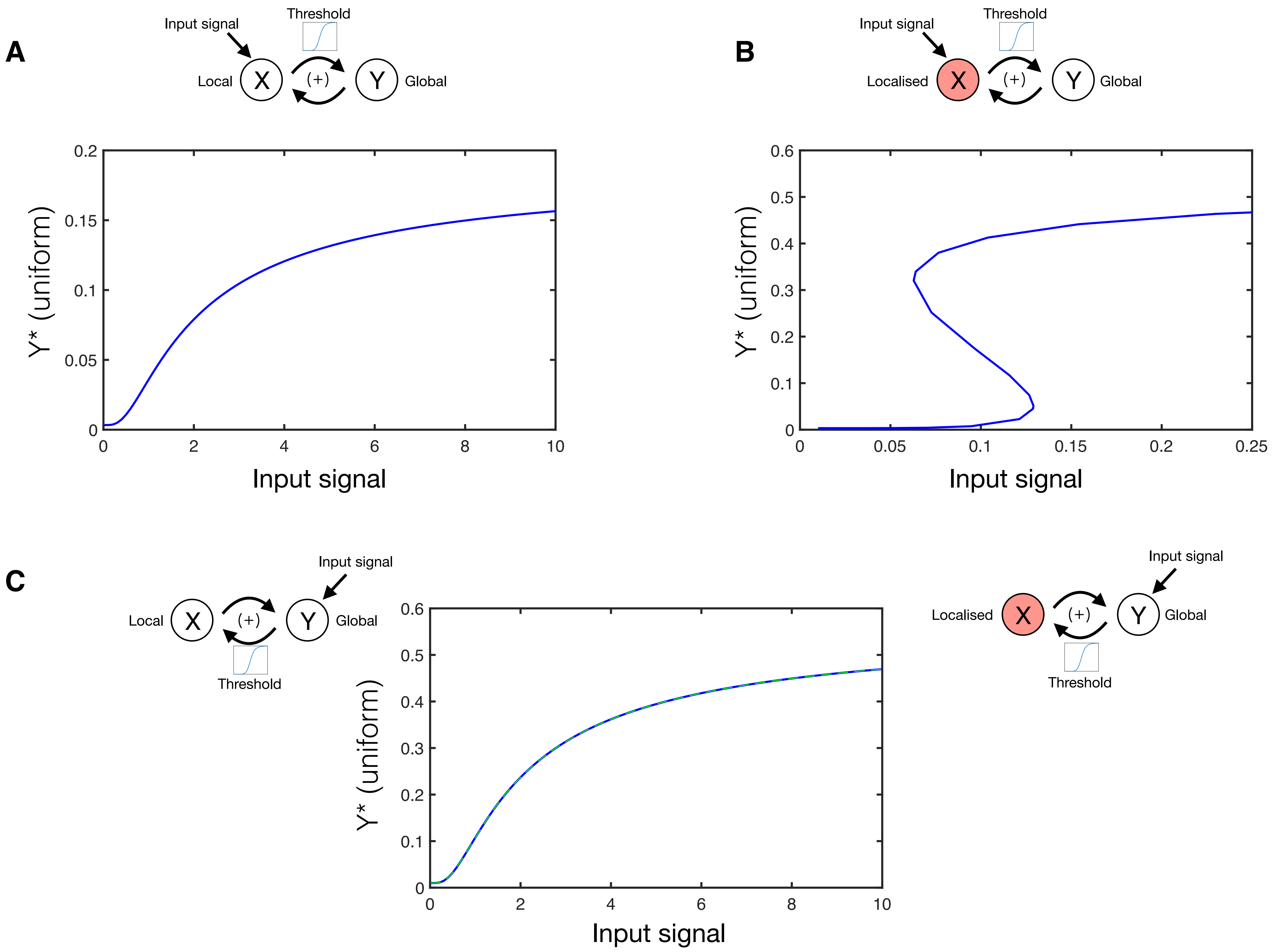

Supplementary Figure 7: Rewiring to circumvent kinetic constraints - two node positive feedback motif with threshold behaviour in the activation of global node Y by local node X.
(A) Without localization, the system is monostable (homogeneous steady state). (B) Localization of the local node X (keeping the total amount the same) allows the motif to exhibit bistable behaviour. (C) If the threshold behaviour occurs in the opposite direction, i.e. in the activation of local node X by a global node Y, localizing the X node has a purely neutral effect, and does not enable bistability.

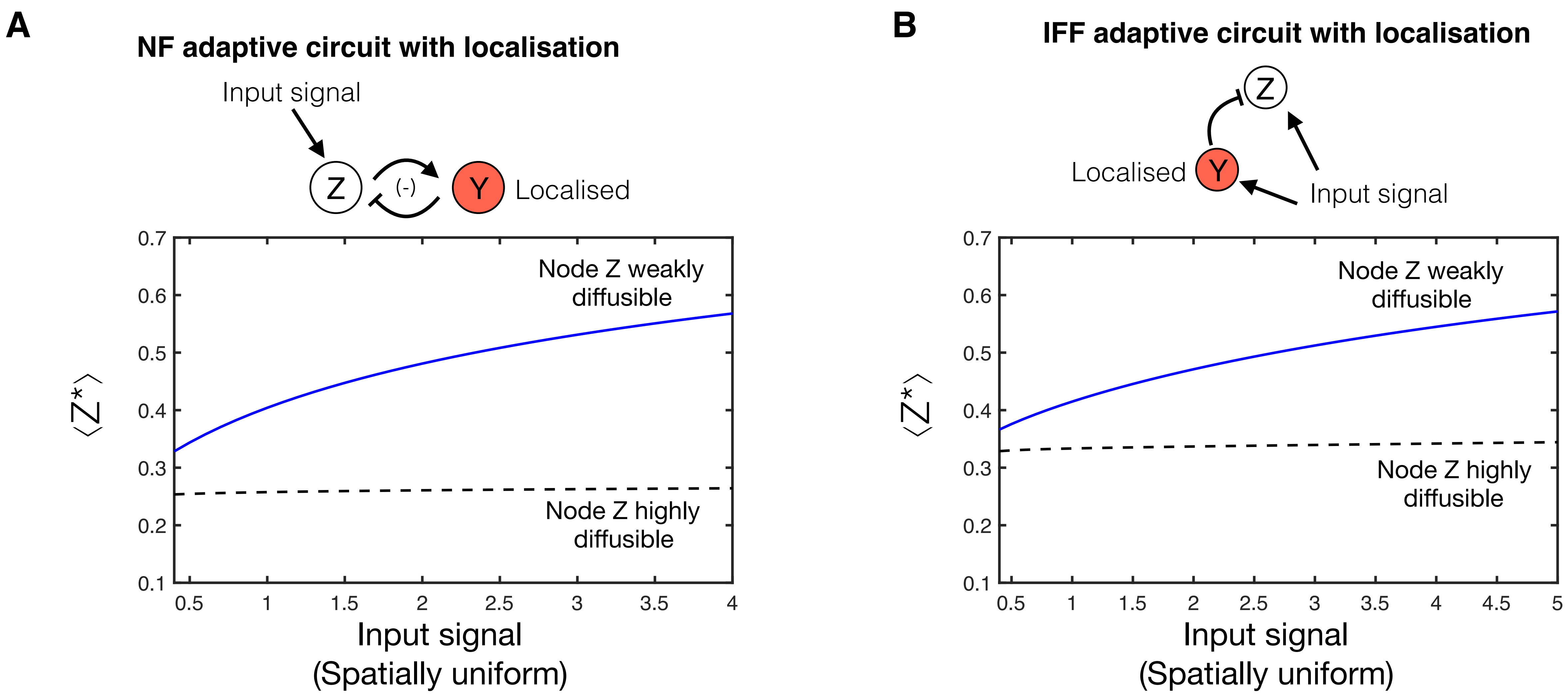

Supplementary Figure 8: Effect of localization on adaptive/homeostatic motifs. For both the negative feedback motif with the feedback node localized (A), and the incoherent feedforward motif with one of the feedforward interactions localized (B), a weakly diffusible output node can prevent adaptive behaviour, even in response to a spatially uniform input signal.

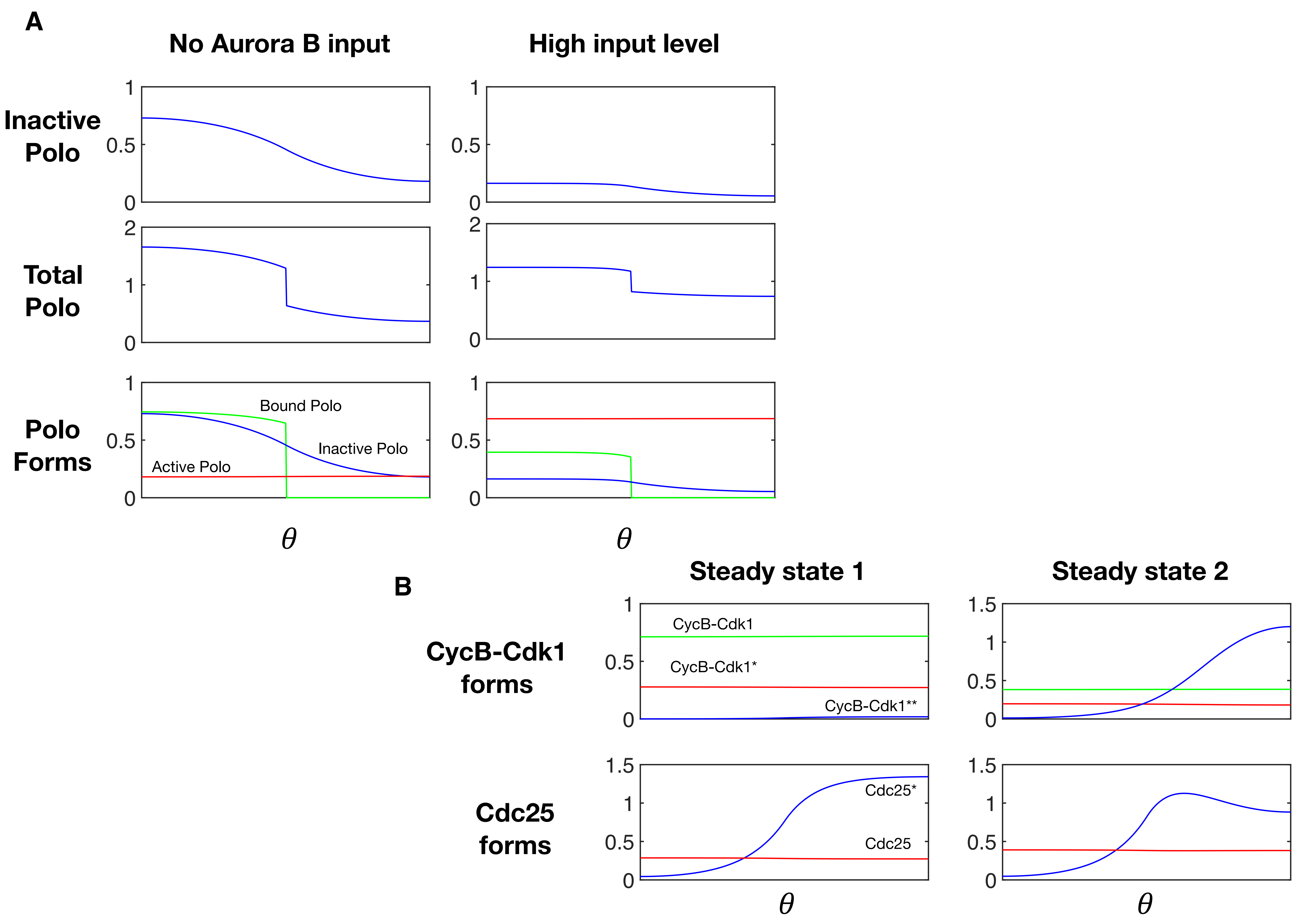

Supplementary Figure 9: Steady state dimensionless concentration profiles of Polo kinase in the model for the Drosophila mitotic switch, and spatial bistability in the Cdc25 - CycB-Cdk1 feedback system.
(A) Steady state profiles - of Polo kinase with and without activation by Aurora B in the cytoplasm - activation brings about a redistribution of Polo which is reflected in the total Polo concentration profile. Note that the discontinuity in the total Polo profile is a result of inactive Polo reversibly binding to Map205, which is assumed to occur only in the cytoplasm. (B) Steady state profiles of the different forms of CycB-Cdk1 and Cdc25 corresponding to two co-existing stable steady states. Switching between states brings about a spatial redistribution of the different forms.

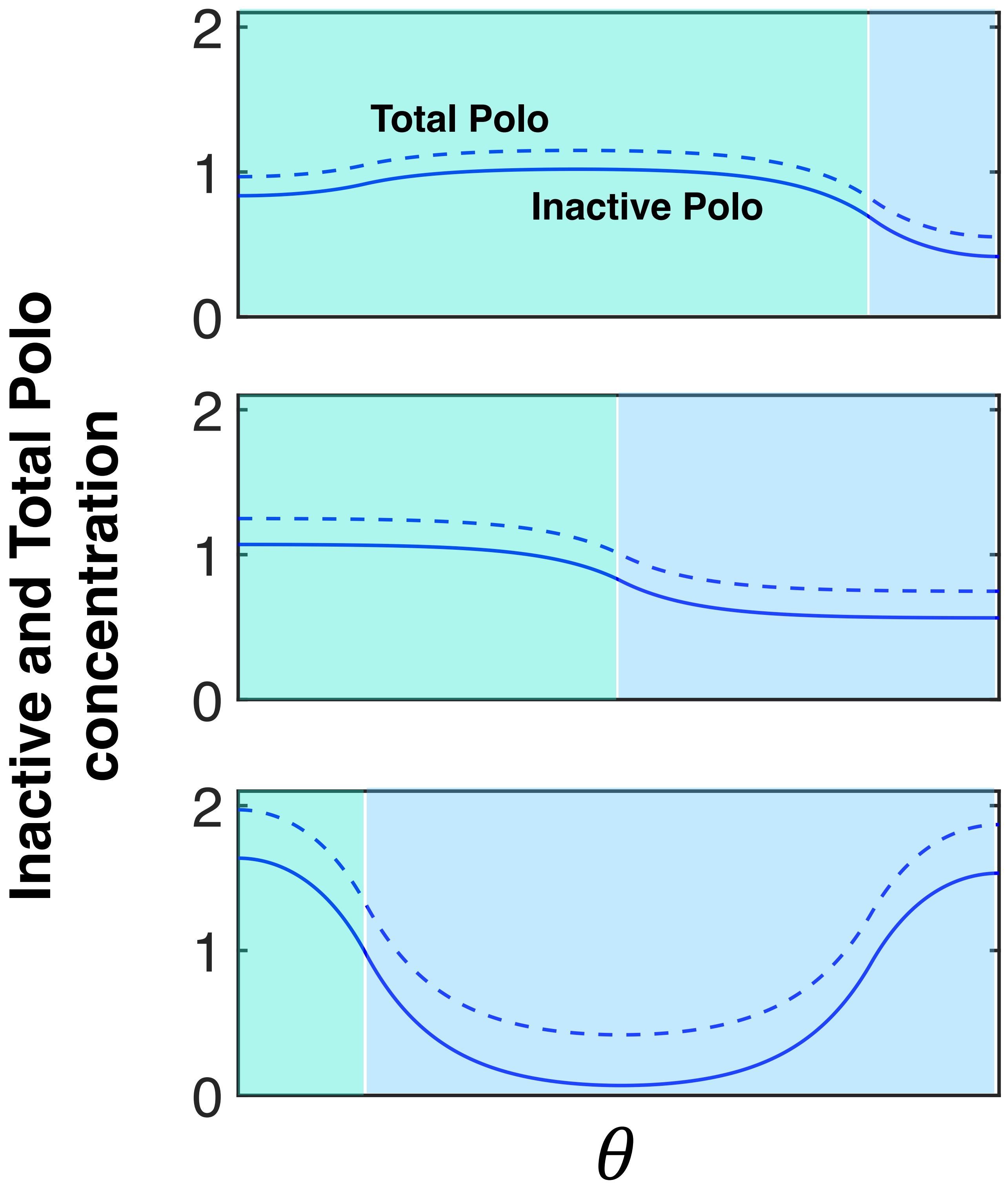

Supplementary Figure 10: Steady state dimensionless concentration profiles for the Polo kinase - Optn spatial feedback loop considered in isolation.
Depending on the relative sizes of the domains (cytoplasm shaded in green, nucleus shaded in blue) this negative feedback can generate non-monotonic spatial profiles of total Polo.

Supplementary Table 1

Section 2.1	Basic effects of localization	Analysis based on simple cause and effect and basic computation to reveal basic conclusions
	Localization and the role of diffusivity for bistable and oscillatory circuits, as well as multiple region localization for incoherent feedforward circuits	Analysis: Basic computation supported by analytical work in multiple instances, explicitly revealing the factors at play
	Localization enables new behaviour	Analysis: While the new behaviour is shown computationally (and can be seen in different parameter sets), the main point is that is something you do not see in the lumped system (ODE)—and this is established analytically
	Localization allows for translating network behaviour into spatial outcomes	Analysis: This is based on computational analysis of a circuit constructed and relies on the trapping of fronts, a phenomenon which is established. The novelty here is the information processing angle and what localization can do to enable this behaviour
Section 2.2	Localization and generation of patterns	Analysis: Computational results show that pattern formation can be readily obtained. Supporting analytical work through compartmental models reveals the underlying factors at work, explicitly
	Position of localization and symmetry breaking	Analysis: Computational work supported by basic analytical work to reveal the underlying functioning and reasons for behaviour
	Control and Selection of Patterns	Analysis: The analysis is computational, but the circuit is constructed based on a basic intuition regarding enabling or preventing certain patterns for a very basic reason which is discussed in Supplementary Material
Section 2.3	Basic effects	Analysis: Computations on basic models, complemented by analytical work
	Incorrect inference of feedback circuits	Analysis: Computation and analytical work showing that with localization the behaviour of the system is opposite to that of the ODE model (describing the nominal feedback structure). This has been further generalized with additional computation and analytical work
Section 2.4	Impact of localization on homeostatic circuits	Analysis: Transparent revealing of cause and effect via computations and supporting analytical work
	Buffering against distance	Analysis: A circuit constructed based on analytical work, and confirmed computationally
	Rewiring enabling modular combination	Analysis: A basic set of circuits was chosen and computation shows that modular combination can be achieved. This is ultimately based on the idea that different circuits can be combined "downstream", bypassing the kinetic constraints which prevent both behaviour being seen (Constructive approach)
	Alleviation of kinetic constraints by local elevation	Analysis: Computation and analytical work on basic prototype circuits: ultimately this relies on a basic feature of elevation of concentration—when that can and cannot help alleviate the kinetic constraint. The elevation of concentration of specific species (nodes) enables thresholds to be crossed

Supplementary Note 8: Models and Parameter Values

NOTE 1: The way the elementary interactions are modelled is presented in the Methods. Here we present the model equations and parameter values corresponding to each figure. In each case the network model is built up by combining the elementary interactions as discussed in the Methods section. For the network models used in each figure, we present the lines Matlab code used to describe the partial time derivatives of each species. The notation used here is as follows: for example, `fX` represents the (partial) time derivative of X*. The discretized Laplacian in the diffusion term is represented by ‘`nfxlap`’ (for no-flux boundary conditions) or ‘`perlap`’ (periodic boundary conditions). These are calls to the Matlab functions shown below, which returns a vector containing the values of the discretized second spatial derivative at the grid points.

NOTE 2: For each of the four sections in the paper, we provide one example below of the full Matlab function code containing the discretized time derivatives for a spatially distributed network. Matlab files containing these example functions, as well as the functions for the discretized Laplacian, are available for download at: https://github.com/gm1613/LocalizationMeetsNetworks.git

These examples provide a template for Matlab functions: the lines of Matlab code provided below (corresponding to each figure) can be incorporated into these templates to create Matlab functions specific to each figure. For all cases presented in the paper, the discretized time derivative equations were solved using the Matlab ODE solver `ode15s`.

NOTE 3: The bifurcation analysis for ODE models - was performed using Matcont - a Matlab package for numerical bifurcation analysis. Numerical equilibrium continuation for discretized PDE models - used for the analysis in Fig. 3(C) and Fig. 7(F) for instance - was performed using custom written code in Matlab, using a pseudo-arclength continuation algorithm.

NOTE 4: In the code, unless otherwise specified, we use the variable names X, Y, Z etc. or x,y,z etc. to represent the concentrations of the active forms in the model, i.e. X*, Y*, Z*. For each figure, the parameter values used - for the spatial domain size, localized subdomain sizes, diffusivities, and kinetic rate constants, are also presented.

Matlab function to compute 1-D Laplacian with no-flux BCs: nfxlap.m

```
%% Second spatial derivative for no-flux BCs
%%%%%%%%%%%%%%%%%%%%%%%%%%%%%%%%%%%%%%%%%%%%%%%%%%%%%%%%%%%
% ** Using 3 point central difference approximation for
%    second spatial derivative.
% ** No-flux boundary conditions imposed using a 2 point
%    central difference approximation and a fictitious node
%    at each boundary.
% ** x is a vector of concentrations at the grid points
%    across the domain
```

```
% ** delx is the interval size for the discretization
%%%%%%%%%%%%%%%%%%%%%%%%%%%%%%%%%%%%%%%%%%%%%%%%%%%%%%%%%%%

function f = nfxlap(x,delx)
f = ([x(2:end);x(end-1)] - 2*x + [x(2);x(1:end-1)])/delx^2;
end
```

Matlab function to compute 1-D Laplacian with periodic BCs: perlap.m

```
%% Second spatial derivative for periodic BCs
%%%%%%%%%%%%%%%%%%%%%%%%%%%%%%%%%%%%%%%%%%%%%%%%%%%%%%%%%%%
% ** Using 3 point central difference approximation for
%    second spatial derivative.
% ** x is a vector of concentrations at the grid points
%    across the domain
% ** delx is the interval size for the discretization
%%%%%%%%%%%%%%%%%%%%%%%%%%%%%%%%%%%%%%%%%%%%%%%%%%%%%%%%%%%

function f = perlap(x,delx)
f = (circshift(x,1) - 2*x + circshift(x,-1))/delx^2;
end
```

Models and Parameter values

FIGURE 2(C,D):

```
fX = (k3*(Xt-X) - k4*X).*Lc1 + Dx*nfxlap(X,delx);
fY = (k1*X.*(Yt-Y)).*Lc1 - k2*Y + Dy*nfxlap(Y,delx);

PARAMETER VALUES:
k1 = 5;
k2 = 0.4;
k3 = 1;
k4 = 0.5;
Yt = 1;
Xt = 1;

Dx = 0;
Dy = 0.01; %Diffusivity
```

Complete Matlab function for FIGURE 2 (C,D):

```
function f = single_node_localised_activation(t, v)
% v is a vector containing concentration profiles
% for all the species in the model.
%% Spatial domain:
%Domain Size
L = 1;
% Define subdomain of localisation
Lc1 = [zeros(163,1);ones(36,1);zeros(162,1)];
%Lc1 = [zeros(18,1);ones(36,1);zeros(307,1)];
%% PARAMETER VALUES:
k1 = 5;
k2 = 0.4;
k3 = 1;
k4 = 0.5;
Yt = 1;
Xt = 1;

Dx = 0;
Dy = 0.01; %Diffusivity

% Finite difference parameters
Numgrds = 361; %For no-flux BCs

delx = L/(Numgrds-1);
```

```
%% Extracting concentration profiles of individual species from vector v
XM = reshape(v,[],2);
X = XM(:,1);
Y = XM(:,2);
%% Finite differenced equations
fX = (k3*(Xt-X) - k4*X).*Lc1 + Dx*nfxlap(X,delx);
fY = (k1*X.*(Yt-Y)).*Lc1 - k2*Y + Dy*nfxlap(Y,delx);

f = [fX;fY];
end
```

FIGURE 2(B):

ONE INTERACTION LOCALISED: MUTUAL ACTIVATION

```
fX = k1x*S*(Xt-X).*Y - k2x*X -kdx*X + Dx*nfxlap(X,delx);
fY = k0y*(Yt-Y) + (k1y*(Yt-Y)).*X.*Lc1 - k2y*Y + Dy*nfxlap(Y,delx);
```

ONE INTERACTION LOCALISED: MUTUAL INHIBITION

```
fX = k1x*S*(Xt-X) - k2x*X.*Y - kdx*X + Dx*nfxlap(X,delx);
fY =k0y*(Yt-Y) + (k1y*(Yt-Y)).*1 - k2y*Y.*X.*Lc1 + Dy*nfxlap(Y,delx);
```

ONE INTERACTION LOCALISED: NEGATIVE FEEDBACK

```
fX = k1x*S*(Xt-X) - k2x*X.*Y -kdx*X + Dx*nfxlap(X,delx);
fY = k0y*(Yt-Y) + (k1y*(Yt-Y)).*X.*Lc1 - k2y*Y + Dy*nfxlap(Y,delx);
```

ONE (OTHER) INTERACTION LOCALISED: NEGATIVE FEEDBACK

```
fX = k1x*S*(Xt-X) - k2x*X.*Y.*Lc1 -kdx*X + Dx*nfxlap(X,delx);
fY = k0y*(Yt-Y) + (k1y*(Yt-Y)).*X - k2y*Y + Dy*nfxlap(Y,delx);
```

ONE NODE LOCALISED: MUTUAL INHIBITION

```
fX = k1x*S*(Xt-X) - k2x*X.*Y -kdx*X + Dx*nfxlap(X,delx);
fY =(k0y*(Yt-Y) + (k1y*(Yt-Y)).*1 - k2y*Y.*X).*Lc1 + Dy*nfxlap(Y,delx);
```

PARAMETER VALUES:

```
L = 1; %Domain Size
k1x = 1;
k2x = 1;
k1y = 1;%1
```

```
k2y = 1;
k0y = 1*1e-3;
kdy = 1*1e-3;
kdx = 1*1e-3;

Xt = 1;
Yt = 1;

Dx = 1e-2;
Dy = 1e-2;
```

FIGURE 2(E):

```
fY = k1y*(Yt-Y).*X - k2y*Y + Dy*nfxlap(Y,delx);
fX = Lc1.*(k1x*Y.*(Xt-X) - k2x*X);
PARAMETER VALUES:
k1x = 1;
k2x = 0.5;
k1y = 1;
k2y = 0.5;

Xt = 1;
Yt = 1;

Dy = 0.01;
```

FIGURE 2(F): (Diffusivity dependent bistability)

```
fx = k0x*S.*(Xt-X) + k1*(Y.^2).*(Xt-X)./(K1+Y.^2) - k2*X + Dx*nfxlap(X,delx);
fy = k0y*(Yt-Y) + k3*(X.^2).*(Yt-Y)./(K3+X.^2) - k5*Y + Dy*nfxlap(Y,delx);

INPUT SIGNAL 'S' IS LOCALISED: S = signal_level*Lc1, where
Lc1 = [zeros(126,1);ones(109,1);zeros(126,1)];

PARAMETER VALUES:
L = 1; %Domain Size
k0x = 0.04;
k0y = 1e-2;
k1 = 0.8;
k2 = 0.5;
K1 = 0.2;
k3 = 1.8;
k5 = 1;
K3 = 0.4;
```

```
Xt = 1;
Yt = 1;

CASE 1: Dx = 1e-3; Dy = 1e-3;
CASE 2: Dx = 5e-3; Dy = 5e-3;
CASE 3: Dx = 1e-2; Dy = 1-2;
```

FIGURE S2(C): (interactions co-localised) and (interactions apart)

Co-localised

```
fx = k0x*S.*(Xt-X) + k1*(Y.^2).*(Xt-X)./(K1+Y.^2).*Lc1 - k2*X + Dx*nfxlap(X,delx);
fy = k0y*(Yt-Y) + k3*(X.^2).*(Yt-Y)./(K3+X.^2).*Lc1 - k5*Y + Dy*nfxlap(Y,delx);
Lc1=[zeros(144,1);ones(72,1);zeros(144,1)];
```

Apart

```
fx = k0x*S.*(Xt-X) + (k1*(Y.^2).*(Xt-X)./(K1+Y.^2)).*Lc1 - k2*X + Dx*nfxlap(X,delx);
fy = k0y*(Yt-Y) + (k3*(X.^2).*(Yt-Y)./(K3+X.^2)).*Lc2 - k5*Y + Dy*nfxlap(Y,delx);

Lc1=[zeros(36,1);ones(72,1);zeros(144+109,1)];
Lc2 = [zeros(144+109,1);ones(72,1);zeros(36,1)];
PARAMETER VALUES:
L = 1; %Domain Size
k0x = 0.04;
k0y = 1e-2;
k1 = 0.8;
k2 = 0.5;
K1 = 0.2;
k3 = 1.8;
k5 = 1;
K3 = 0.4;
Xt = 1;
Yt = 1;
Dx = 1e-3;
Dy = 1e-3;
```

FIGURE 2(G): (Location dependent bistability)

```
fx = k0x*(Xt-X) + k1*(Y.^2).*(Xt-X)./(K1+Y.^2) - k2*X + Dx*nfxlap(X,delx);
fy = (k0y*S*(Yt-Y) + k3*(X.^2).*(Yt-Y)./(K3+X.^2) - k5*Y).*Lc1;
```

The location of the localised node Y is varied by changing the array Lc1.Size of localised subdomain is fixed at 1/5 of total domain size.

```
PARAMETER VALUES:
L = 1; %Domain Size
k0x = 0.01;%0.04
```

```
k0y = 4e-2;%1e-2
k1 = 0.8;
k2 = 0.25; %0.5
K1 = 0.2;
k3 = 1.8;
k5 = 1;
K3 = 0.4;
Xt = 1;
Yt = 1;
Dx = 1e-2;
```

FIGURE S2(B): motif_24.m (Location dependent oscillations)

```
fx = k0*(1-2*X) + k1*(1-X) - k2*X.*hf(Z,K2) + Dx*nfxlap(X,delx);
fy = k0*(1-2*Y) + k3*(1-Y).*hf(X,K3) - k4*Y + Dy*nfxlap(Y,delx);
fz = (k0*(1-2*Z) + k5*(1-Z).*hf(Y,K5) - k6*Z).*Lc1 + Dz*nfxlap(Z,delx);
```

Node Z is localised at different locations defined by the arrays Lc1.
For location close to boundary, Lc1 = [zeros(2,1);ones(72,1);zeros(145+142,1)];
For location at centre, Lc1 = [zeros(144,1);ones(72,1);zeros(145,1)];

PARAMETER VALUES:

```
k0 = 1e-3;
k1 = 0.4;
k2 = 6;
K2 = 0.01;
k3 = 1;
K3 = 0.2;
k4 = 1;%1
k5 = 2;
K5 = 0.01;
k6 = 1;
hf = @(x,K) (x.^4)./(K+x.^4);
Dx = 0.005;
Dy = 0.005;
Dz = 0;
```

FIGURE S5(A-B): motif03_modified_spatial.m (Limit cycle Oscillations in a two node motif)

```
fX = (k0 + k1*S).*(1-X) - k3*Lc1.*X.*hf1(Y) + Dx*perlap(X,delx);
```

```
fY = k2*hf2(X).*Lc2.*(1-Y) - k4*Y + Dy*perlap(Y,delx);
```

The two interactions are localised at different locations defined by the arrays Lc1 and Lc2.

```
Lc1 = [zeros(53,1);ones(73,1);zeros(234,1)];
Lc2 = [zeros(233,1);ones(73,1);zeros(54,1)];

PARAMETER VALUES:
L = 1; %Domain Size
k0 = 0.1;
k1 = 0.1;
k2 = 10;
k3 = 100;
k4 = 1;
v = 4;%4
u = 8;%6
K1 = 5/u^4;
K2 = 5/v^4;
hf1 = @(X) ((X).^4)./(K1 + (X).^4);
hf2 = @(X) ((X).^4)./(K2 + (X).^4);
Dx = 1e-1;
Dy = 1e-1;
```

'S' is the input signal. For Fig. S5(A), S=0; For Fig. S5(B), S=1.5±1*sin(2*pi*pos).

FIGURE S5(C): motif_29_loc2 (Diffusivity dependent oscillations; Node Y localised at centre)

```
fx = ((k4*(y.^2).*(1-x)./(K4+y.^2))- k5*x +k0*(1-2*x)) + Dx*nfxlap(x,delx);
fy = (k1*x.*(1-y) - k2*z.*y  + k0*(1-2*y)).*Lc1 + Dy*nfxlap(y,delx);
fz = ((k7*(y.^2).*(1-z)./(K7+y.^2)) - k8*z + 0*k0*(1-2*z)) + Dz*nfxlap(z,delx);

PARAMETER VALUES:
L = 8; %Domain Size
k0 = 1e-3;
k1 = 0.5;
k2 = 3;
k4 = 2;
k5 = 1.5;
k7 = 0.5;
k8 = 0.06;
K4 = 0.05;
```

```
K7 = 0.1;
Lc1 = [zeros(144,1);ones(72,1);zeros(145,1);] %Localisation Size=⅕; Location=centre

CASE 1: HIGH DIFFUSIVITY
Dx = 5;
Dy = 0;
Dz = 5;
CASE 2: INTERMEDIATE DIFFUSIVITY
Dx = 1e-1;
Dy = 0;
Dz = 1e-1;
CASE 3: LOW DIFFUSIVITY
Dx = 1e-3;
Dy = 0;
Dz = 1e-3;
```

FIGURE 3(A-D): bisMIcdc_1dspatial.m

```
fx = a1*Z*(Xt-X).*Lc1 - (b1*X.*(Y).^4)./(K1+(Y).^4) + Dx*nfxlap(X,delx);
fy = a2*Z*(Yt-Y).*Lc2 - (b2*Y.*X.^4)./(K2+X.^4) + Dy*nfxlap(Y,delx);
fz = kz*I*(Zt-Z) - kdz*z + Dz*nfxlap(z,delx);
```

Yt is the bifurcation parameter.

```
PARAMETER VALUES:
L = 1; %DOMAIN SIZE
a1 = 2;
a2 = 2;
b1 = 200;
b2 = 10;
kz = 0.1;
Kdz = 0.1;
K1 = 30;
K2 = 1;
Xt = 1;
Zt = 1;
I = 1;
Dx = 1e-2;
Dy = 1e-2;
Dz = 1;

Lc1 = [ones(37,1);zeros(162,1);zeros(162,1)];%Localisation Size=1/10;
Location=adjacent to boundary (no-flux boundary)
Lc2 = [zeros(162,1);zeros(162,1);ones(37,1)]; %Localisation
Size=1/10;Location=adjacent to other boundary (no-flux boundary)
```

FIGURE 3(E-F): motif08_spatial.m (Trapped fronts - localisation enables spatial switching)

```
fX = (k0 + k1.*I.*hf1(Z)).*(1-X) - k4.*X - k6*X.*Y + Dx*perlap(X,delx);
fY = Lc1.*((k0 + k3).*(1-Y) - k7*kdy*Y) + Dy*perlap(Y,delx);
fZ = (k0 + k2.*hf1(X)).*(1-Z) - k5.*Z - kzy*Z.*Y + Dz*perlap(Z,delx);
```

PARAMETER VALUES:

```
L = 1; %DOMAIN SIZE
k0 = 0.5;
k1 = 10;
k2 = 100;
k3 = 1;
k4 = 5;
k5 = 5;
k6 = 600;
k7 = 1;
K1 = 0.5;
kzy = 0.01;
kdy = 2;
hf1 = @(X) (X.^4)./(K1 + X.^4);
Dx = 1e-1;
Dy = 0;
Dz = 1e-1;
```

INPUT SIGNAL: I = (5*(ones(Numgrds,1)) + Amp*cos(2*pi*xvec + phi)); where the amplitude is 0 for the uniform input signal and 3 for the grade input signal.

FIGURE 4: motif_29a.m (Localisation induced instability of homogeneous ss)

EQUATIONS:

```
fx = (k4*(y.^2).*(1-x)./(K4+y.^2))-k5*x +k0*(1-2*x) + Dx*nfxlap(x,delx);
fy = k1.*x.*(1-y) - k2*z.*y  + k0*(1-2*y) + Dy*nfxlap(y,delx);
fz = Lc1.*((k7*(y.^2).*(1-z)./(K7+y.^2)) - k8*z) + Dz*nfxlap(z,delx);
```

PARAMETER VALUES:

```
L = 5; %Domain Size
k0 = 1e-3;
k1 = 0.1;
k2 = 3;
k4 = 2;
k5 = 1.5;
k7 = 0.5;
```

```
k8 = 0.08;
K4 = 0.05;
K7 = 0.1;
```

Complete Matlab function for the motif shown in Figure 4:

```
function f = motif_29a(t, v)
%% Domain parameters
% Channel Length
L = 5;

Lc1 = [zeros(289,1);ones(144,1);zeros(288,1)];%% Lc2 =
Lc1;%circshift(Lc1,-288);% circshift(Lc1,216);
%% Parameters
k0 = 1e-3;
k1 = 0.1;%0.7
k2 = 3;
k4 = 2;
k5 = 1.5;
k7 = 0.5;
k8 = 0.08;
K4 = 0.05;
K7 = 0.1;

Dx = 0.01;%0.01,0.001
Dy = 0.01;
Dz = 0.01;


%% Extracting variables

Numgrds = 721; %For no-flux BCs

delx = L/(Numgrds-1);

XM = reshape(v,[],3);
x = XM(:,1);
y = XM(:,2);
z = XM(:,3);

%% Finite differenced equations
```

```
fx = (k4*(y.^2).*(1-x)./(K4+y.^2))-k5*x +k0*(1-2*x) + Dx*nfxlap(x,delx);
fy = k1.*x.*(1-y) - k2*z.*y  + k0*(1-2*y) + Dy*nfxlap(y,delx);
fz = Lc1.*((k7*(y.^2).*(1-z)./(K7+y.^2)) - k8*z) + Dz*nfxlap(z,delx);

% Remember to check order of multiplication of signal vectors or
% localisation vectors with the variables


f=[fx;fy;fz];
end
```

CASE 1 (Fig. 4E)

```
Lc1 = [zeros(289,1);ones(144,1);zeros(288,1)]; %Localisation Size=⅕; Location=centre
Dx = 0.01;
Dy = 0.01;
Dz = 0.01;
```

PART 1 INITIAL CONDITIONS:

```
X0 = 1*ones(Numgrds,1);
Y0 = 1*ones(Numgrds,1);
Z0 = 0*ones(Numgrds,1);
```

PART 2 INITIAL CONDITIONS:

```
X0 = 0.5*tanh(10*(2*pos-5))+0.5;
Y0 = 0.5*tanh(10*(2*pos-5))+0.5;
Z0 = 0.5*tanh(10*(2*(5-pos)-5))+0.5;
```

PART 3 INITIAL CONDITIONS:

```
X0 = 0.5*tanh(10*(2*(5-pos)-5))+0.5;
Y0 = 0.5*tanh(10*(2*(5-pos)-5))+0.5;
Z0 = 0.5*tanh(10*(2*(pos)-5))+0.5;
```

OBSERVATIONS: Homog. SS is Unstable (From simulation and computing eigenvalues of Jacobian); Two symmetry broken stable ss; Stable “high-high” ss;

CASE 2

```
Lc1 = [zeros(289,1);ones(144,1);zeros(288,1)]; %Localisation Size=⅕; Location=centre
Dx = 0.013;
Dy = 0.013;
```

```
Dz = 0.013;
```

OBSERVATIONS: Homog. SS is Stable (From simulation and computing eigenvalues of Jacobian); Two symmetry broken stable ss; Stable “high-high” ss;

CASE 3 (Fig. S4)

```
Lc1 = [zeros(289,1);ones(144,1);zeros(288,1)]; %Localisation Size=⅕; Location=centre
Dx = 0.016;
Dy = 0.016;
Dz = 0.016;
```

OBSERVATIONS: Homog. SS is Stable (From simulation and computing eigenvalues of Jacobian); Two symmetry broken stable ss; Stable “high-high” limit cycle oscillations;

CASE 4

```
Lc1 = [zeros(289,1);ones(144,1);zeros(288,1)]; %Localisation Size=⅕; Location=centre
Dx = 0.02;
Dy = 0.02;
Dz = 0.02;
```

OBSERVATIONS: Homog. SS is Stable (From simulation and computing eigenvalues of Jacobian); Two symmetry broken stable ss; Apparently no “high-high” ss or oscillations;

Bifurcation analysis of compartmental ODE models (FIG. S4):

FIG. S3 (B) THREE COMPARTMENT MODEL (compartment order 2-1-3; equal sized compartments):
Node Z interconversion localised to compartment 1; p is the transport parameter; p is used as the bifurcation parameter

```
x1' = (k4*(y1^2)*(1-x1)/(K4+y1^2))-k5*x1+k0*(1-2*x1)-p*(x1-x2)-p*(x1-x3)
y1' = k1*x1*(1-y1)-k2*z1*y1+k0*(1-2*y1)-p*(y1-y2)-p*(y1-y3)
z1' = (k7*(y1^2)*(1-z1)/(K7+y1^2))-k8*z1-p*(z1-z2)-p*(z1-z3)
x2' = (k4*(y2^2)*(1-x2)/(K4+y2^2))-k5*x2+k0*(1-2*x2)+p*(x1-x2)
y2' = k1*x2*(1-y2)-k2*z2*y2+k0*(1-2*y2)+p*(y1-y2)
z2' = p*(z1-z2)
x3' = (k4*(y3^2)*(1-x3)/(K4+y3^2))-k5*x3+k0*(1-2*x3)+p*(x1-x3)
```

```
y3' = k1*x3*(1-y3)-k2*z3*y3+k0*(1-2*y3)+p*(y1-y3)
z3' = p*(z1-z3)

PARAMETER VALUES:
k0 = 1e-3;
k1 = 0.1;
k2 = 3;
k4 = 2;
k5 = 1.5;
k7 = 0.5;
k8 = 0.08;
K4 = 0.05;
K7 = 0.1;
```

TWO COMPARTMENT MODEL (equal sized compartments):
Node Z interconversion localised to compartment 1; p is the transport parameter;
p is used as the bifurcation parameter

```
x1' = ((k4*(y1^2)*(1-x1)/(K4+y1^2))-k5*x1 +k0*(1-2*x1) - p*(x1-x2))
y1' = (k1*x1*(1-y1) - k2*z1*y1 + k0*(1-2*y1) - p*(y1-y2))
z1' = (((k7*(y1^2)*(1-z1)/(K7+y1^2)) - k8*z1 + 0*k0*(1-2*z1)) - p*(z1-z2))
x2' = ((k4*(y2^2)*(1-x2)/(K4+y2^2))-k5*x2 +k0*(1-2*x2) + p*(x1-x2))
y2' = (k1*x2*(1-y2) - k2*z2*y2 + k0*(1-2*y2) + p*(y1-y2))
z2' =  (p*(z1-z2))

PARAMETER VALUES:
k0 = 1e-3;
k1 = 0.1;
k2 = 3;
k4 = 2;
k5 = 1.5;
k7 = 0.5;
k8 = 1.76;%0.08
K4 = 0.05;
K7 = 0.1;
```

FIGURE 5(B-D): (Hybrid pattern formation)

```
fx = ((k4*(y.^2).*(1-x)./(K4+y.^2))-k5*x +k0*(1-2*x))+ Dx*perlap(x,delx);
fy = (k1*x.*(1-y) - k2*z.*y.*1  + k0*(1-2*y)) + Dy*perlap(y,delx);
fz = ((k7*(y.^2).*(1-z)./(K7+y.^2)) - k8*z + k0*(1-2*z)) + Dz*perlap(z,delx);
```

```
fa = (k1a*S.*(at-a)-k2a*a).*Lc1+ Da*perlap(a,delx);
fb = (k1b*S.*(bt-a)-k2b*b).*Lc2+ Db*perlap(b,delx);
fc = k0c*(ct-c)+kac*a.*(ct-c)-kbc*b.*c-kdc*c+ Dc*perlap(c,delx);

PARAMETER VALUES:
k0 = 1e-3;
k1 = 0.7;%0.7,0.5
k2 = 3;
k4 = 0.5;
k5 = 1.5;%1.5
k7 = 0.5;
k8 = 0.45;%0.08%0.4today
K4 = 0.05;
K7 = 0.1;

k1a = 0.1;
k2a = 1;
at = 10;
k1b = 0.1;
k2b = 1;
bt = 10;
k0c = 0.1;
kac = 1;
kbc = 1;
kdc = 0.2;
ct = 1;


Dx = 1e-3;%1e-2 for both X and Y
Dy = 1e-3;%(1e-1)
Dz = 1;%0.1
Da = 0;
Db = 0;
Dc = 10;

Lc1 = [zeros(72,1);ones(36,1);zeros(253,1)];%Localisation Size=1/10;
Lc2 = [zeros(253,1);ones(36,1);zeros(72,1)];%Localisation Size=1/10;
```

FIGURE 5(E-F): (Feedback control of pattern formation)

```
fx = ((k4*(y.^2).*(1-x)./(K4+y.^2))-k5*x +k0*(1-2*x)) + Dx*perlap(x,delx);
fy = (k1*x.*(1-y) - k2*z.*y.*1 + 0*(t<5000)*(1-y).*Lc2  + k0*(1-2*y)) +
Dy*perlap(y,delx);
```

```
fz = ((k7*(y.^2).*(1-z)./(K7+y.^2)) - k8*z - S*kg*h.*z + k0*(1-2*z)) +
Dz*perlap(z,delx);
fg = ((kh*(y.^4).*(1-g)./(Kh+y.^4))-k5*g).*Lc1;
fh = 5*g.*(1-h) - 0.06*36*h.*Lc1+ Dh*perlap(h,delx);

PARAMETER VALUES:
L = 24;
k0 = 1e-3;
k1 = 0.7;%0.7,0.5
k2 = 3;
k4 = 0.5;
k5 = 1.5;%1.5
k7 = 0.5;
k8 = 0.03;%0.08, 0.03, 0.06
K4 = 0.05;
K7 = 0.1;
kg = 0.2;
kh = 1;
Kh = 0.001/(3^4);


Dx = 1e-2;
Dy = 1e-2;%(1e-1)
Dz = 1;%0.1
Dh = 10; %10
```

FIGURE 6 (E,F,G):
Slow diffusing active form (X*) represented by ‘x’ and fast diffusing inactive form (X) represented by y.
Case 1: Localised phosphatase (represented by rate constant k1)

```
fx = -k0*x - k1*x.*L1 + k2*y.*1 + Dx*nfxlap(x,delx);
fy = -k2*y.*1 + k1*x.*L1 + k0*x + Dy*nfxlap(y,delx);

PARAMETER VALUES:
L = 1; %DOMAIN SIZE
k0 = 0.01; % Background phosphatase
k2 = 1;%Background kinase
Dx = 1e-3; %X* active form
Dy = 10;   %X inactive form
Lc1 = [zeros(162,1);ones(36,1);zeros(163,1)];%Localisation Size=1/10; Location=centre

FOR THE DIFFERENT PROFILES: k1=0.01,0.05,0.1,0.5,1
```

Case 2: Localised kinase (represented by rate constant k1)

```
fx = -k0*x + k1*y.*L1 + k2*y.*1 + Dx*nfxlap(x,delx);
fy = -k2*y.*1 - k1*y.*L1 + k0*x + Dy*nfxlap(y,delx);

PARAMETER VALUES:
L = 1; %DOMAIN SIZE
k0 = 0.1; % Background phosphatase
k2 = 0.1;%Background kinase
Dx = 1e-3; %X* active form
Dy = 10;   %X inactive form
Lc1 = [zeros(162,1);ones(36,1);zeros(163,1)];%Localisation Size=1/10; Location=centre

FOR THE DIFFERENT PROFILES: k1=0.01,0.5,1,2
```

FIGURE 7: (Two-diffusivity node; negative to positive feedback)

```
fx = -h2(z).*x.*L1 - k0*x + k2*y.*1 + Dx*nfxlap(x,delx);
fy = -k2*y.*1 + h2(z).*x.*L1 + k0*x + Dy*nfxlap(y,delx);
fz =  h1(x).*(1-z) - kd*z  + Dz*nfxlap(z,delx);
```

x represents the slow diffusing active form; y represents the fast diffusing inactive form; z represents the active form at the feedback node (both forms equally diffusible at this node.

```
PARAMETER VALUES:
L = 1; %DOMAIN SIZE
k0 = 0.01;
kdz = 2;
kdx = 0.5;
Kz = 15;
Kx = 0.2;
k2 = 0.6;
kd = 0.3;
kz1 = 1;
Dx = 0.001;
Dy = 10;
Dz = 1;
h1 = @(x) (kdz.*x.^8)./(Kz+x.^8);%HILL FUNCTION WITH SHARP THRESHOLD
h2 = @(z) (kdx.*z.^4)./(Kx+z.^4);%HILL FUNCTION
```

INITIAL CONDITIONS FOR STEADY STATE DEPICTED IN FIG.7 (E):

PART 1: x0 = [0*(1+0.01*cos(2*pi*pos'));1*ones(Numgrds,1);1*ones(Numgrds,1)];

PART 2: x0 = [1*(1+0.01*cos(2*pi*pos'));0*ones(Numgrds,1);1*ones(Numgrds,1)];

FIG.7 (F): The parameter 'k2' above is used as the bifurcation parameter. 'k2' is the first order rate constant representing conversion to active form by a spatially uniform kinase.

Complete Matlab function for the spatially distributed motif with localised interaction shown in Figure 7(D):

```
function f = single_mod_two_diff3(t, v)
%% Domain parameters
% Channel Length
L = 1;
% Patch location and size
L1 = [zeros(135,1);ones(90,1);zeros(136,1)];
L2 = flipud(L1);
% Lc2 = circshift(Lc1,216);
%% Parameters
k0 = 0.01;
kdz = 2;%2;
kdx = 0.5;
Kz = 15;
Kx = 0.2;
k2 = 0.6;%0.5,0.6
kd = 0.3;
kz1 = 1;

Dx = 0.001;
Dy = 10;
Dz = 1;

h1 = @(x) (kdz.*x.^8)./(Kz+x.^8);
h2 = @(z) (kdx.*z.^4)./(Kx+z.^4);

%% Extracting variables

Numgrds = 361; %For no-flux BCs

delx = L/(Numgrds-1);
```

```
XM = reshape(v,[],3);
x = XM(:,1);%X*:slow diffusing
y = XM(:,2);%X: fast diffusing
z = XM(:,3);


%% Finite differenced equations


fx = -h2(z).*x.*L1 - k0*x + k2*y.*1 + Dx*nfxlap(x,delx);
fy = -k2*y.*1 + h2(z).*x.*L1 + k0*x + Dy*nfxlap(y,delx);
fz =  h1(x).*(1-z) - kd*z  + Dz*nfxlap(z,delx);

f=[fx; fy; fz];
end
```

FIG.7 (I): See supplementary material section 4.6 for the detailed analysis. The parameter values and Hill functions used are:

```
xT = 1;
yT = 1;
k01 = 0.012;
k02 = 0.06;
k2 = 2;
ky = 0.5;
kdy = 2.2;
gamma = 0.8;%0.2,0.8

L=1;
w=0.4;

g = @(x) 5*(x.^8)./(1+x.^8);
h = @(y) 10*(y.^8)./(1+y.^8);
```

FIG.S4 (B,D): See supplementary material section 4.4 for the detailed analysis. The parameter values and Hill functions used are:

```
xT = 1;
yT = 1;
k01 = 0.012;
k02 = 0.01;
k2 = 1;
ky = 1;
```

```
        kdy = 0.2;

        L=1;
        w=0.4;

        g = @(x) 5*(x.^8)./(0.5+x.^8);
        h = @(y) 5*(y.^8)./(1+y.^8);
```

FIGURE 8(A):

```
fz = k1*S'.*(zt-z) - k2*z.*f(y) -kd*z + Dz*nfxlap(z,delx);
fy = Lc1.*(k3*f(z).*(yt-y) - k4*y) + Dy*nfxlap(y,delx);

PARAMETER VALUES:
L = 1; %DOMAIN SIZE
k1 = 0.1;
k2 = 150;
k3 = 5;
k4 = 0.01;
kd = 1e-3;
f = @(x) (x.^8)./(0.2+x.^8); %HILL FUNCTION WITH SHARP THRESHOLD
zt = 1;
yt = 10; %1 when uniform; 10 when localised
Dy = 0;
Dz = 10;

Lc1 = [zeros(162,1);ones(36,1);zeros(163,1)];%Localisation Size=1/10; Location=centre
```

CASE 1: UNIFORM INPUT SIGNAL (S above denotes the input signal)
CASE 2: FIXED SPATIAL AVERAGE OF INPUT SIGNAL (<S>=0.5): S = 0.5*(1+amp*cos(2*pi*pos))
CASE 3: FIXED AMPLITUDE OF INPUT SIGNAL (amp=0.4*<S>): S = avg*(1+0.4*cos(2*pi*pos))

FIGURE 8(B): iff_with_inh_loc.m

```
fz = kz*S'.*(zt-z) - kdz*z.*y -kd*z + Dz*nfxlap(z,delx);
```

```
fy = Lc1.*(ky*S'.*(yt-y) - kdy*y) + Dy*nfxlap(y,delx);
```

```
PARAMETER VALUES:
L = 1; %DOMAIN SIZE
kz = 0.1;
kdz = 10;
ky = 0.1;
kdy = 10;
kd = 1e-3;
zt = 1;
yt = 10; %1 when uniform; 10 when localised
Dy = 0;
Dz = 10;

Lc1 = [zeros(162,1);ones(36,1);zeros(163,1)];%Localisation Size=1/10; Location=centre
```

CASE 1: UNIFORM INPUT SIGNAL (S above denotes the input signal)
CASE 2: FIXED SPATIAL AVERAGE OF INPUT SIGNAL (<S>=0.5): S = 0.5*(1+amp*cos(2*pi*pos))
CASE 3: FIXED AMPLITUDE OF INPUT SIGNAL (amp=0.4*<S>): S = avg*(1+0.4*cos(2*pi*pos))

Complete Matlab function for the Incoherent feedforward motif with localisation in shown in Figure 8(B):

```
function f = iff_with_inh_loc(t, v,avg,amp)
%% Domain parameters
% Channel Length
L = 1;
% Patch location and size

Lc1 = [zeros(162,1);ones(36,1);zeros(163,1)];%


%% Parameters
kz = 0.1;
kdz = 10;
ky = 0.1;
kdy = 10;
kd = 1e-3;

zt = 1;
yt = 10; %1 when uniform; 10 when localised
```

```
Dy = 0;
Dz = 10;



%% Extracting variables

Numgrds = 361; %For no-flux BCs

delx = L/(Numgrds-1);
pos = 0:delx:L;


XM = reshape(v,[],2);
z = XM(:,1);
y = XM(:,2);



%Input signal
S = avg*(1+amp*cos(2*pi*pos));

%% Finite differenced equations

fz = kz*S'.*(zt-z) - kdz*z.*y -kd*z + Dz*nfxlap(z,delx);
fy = Lc1.*(ky*S'.*(yt-y) - kdy*y) + Dy*nfxlap(y,delx);


f=[fz;fy];
end
```

FIGURE 8(D):

```
fz = (kz*S.*(zt-z) - kdz*z.*y).*Lc1 + Dz*nfxlap(z,delx);
fw = (kw*S.*(wt-w) - kdw*w).*Lc2 + Dw*nfxlap(w,delx);
fy = (ky*w.*(yt-y) - kdy*y) + Dy*nfxlap(y,delx);
fx = (kx*z.*(zt-z) - kdx*x) + Dx*nfxlap(x,delx);

kz = 0.1;
kdz = 100;
ky = 0.1;
kdy = 10;
kw = 0.1;
kdw = 10;
kx = 1;
kdx =0.1;
zt = 20;
yt = 100;
wt = 40;
xt = 1;
Dy = 0.01;
Dx = 0.01;
Dz = 0;
Dw = 0;
```

FIGURE 8(F-G): Rewiring

```
fz = (k0+kz*y).*(zt-z)-kdz*z;
fx = (kx0 + k1*z)*(xt-x) -  k2*x;
fy = (k0 + k3*h(x,6)).*(yt-y) - k4*y;
```

```
PARAMETER VALUES:
k0 = 0.001;
kx0 = 0.05;
k1 = 1;
k2 = 0.4;
```

```
k3 = 1;
k4 = 0.3;
kz = 1;
kdz = 1;
h=@(x,n) (x.^n)./(0.01+x.^n);

xt = 0.9;
yt = 1;
zt = 0.9;
```

LOCALISATION IMPOSED BY VECTOR MULTIPLICATION WITH THE ARRAY:

```
Lc1 = [zeros(162,1);ones(36,1);zeros(163,1)];%Localisation Size=1/10; Location=centre
```

NOTE THAT THE TOTAL AMOUNT OF MODIFIED FORMS FOR EACH NODE IS PRESERVED WHEN IMPOSING LOCALISATION.

FIGURE 8(H,I,J):

```
fx = k0*(1-2*X) + k1.*(1-X) - k2*X.*hf(Z,K2) + 5*k7*(1-X).*hf(Y,K5).*Lc1 - 0.5*X +
Dx*nfxlap(X,delx);
fy = k0*(1-2*Y) + k3*(1-Y).*hf(X,K3) - k4*Y  + Dy*nfxlap(Y,delx);
fz = k0*(1-2*Z) + k5*(1-Z).*hf(Y,K5) - k6*Z + Dz*nfxlap(Z,delx);
fw = k0*(1-2*W) + k5*(1-W).*hf(X,K5) - k6*W + Dw*nfxlap(W,delx);
```

PARAMETER VALUES:

```
k0 = 1e-3;
k1 = S;%(0.1 for bistability), (0.4 to 0.8 for oscillations when +ve feedback is OFF)
k2 = 6;%6
K2 = 0.01;
k3 = 1;
K3 = 0.02;
k4 = 1;%1
k5 = 2;
K5 = 0.01;
k6 = 1;
k7 = 2;%(0 or 2)

hf = @(x,K) (x.^4)./(K+x.^4);

Dx = 0.001;%All 0.001 to get both bistability  and oscillations
Dy = 0.001;
Dz = 0.001;
Dw = 1;
Lc1 = [zeros(120,1);ones(120,1);zeros(121,1);]; %Localisation Size=1/3
```

Fig 9(D):

For localised conversion to slow diffusing form, the model equations for the single two-diffusivity node are:

```
fx = k0*y+k1*y.*L2 - k2*x.*1 - k01*x + Dx*nfxlap(x,delx);
fy = -k0*y-k1*y.*L2 + k2*x.*1 + k01*x + Dy*nfxlap(y,delx);

Parameters: (k1 is varied)
L1 = [ones(240,1);zeros(121,1)];
L2 = ~L1;
k2 = 0.1;
k0 = 0.05;
k01 = 0.01;
Dx = 0.01;
Dy = 1;
```

For localised conversion to fast diffusing form, the model equations for the single two-diffusivity node are:

```
fx = k0*y+k1*y.*1 - k2*x.*L1 - k01*x + Dx*nfxlap(x,delx);
fy = -k0*y-k1*y.*1 + k2*x.*L1 + k01*x + Dy*nfxlap(y,delx);

Parameters: (k2 is varied)
L1 = [ones(240,1);zeros(121,1)];
L2 = ~L1;
k1 = 0.1;
k0 = 0.05;
k01 = 0.01;
Dx = 0.01;
Dy = 1;
```

Fig. 9(E,F):

```
Parameters for observed realisation:
     k01 = 0.1;
     k02 = 0.02;
     kpar1 = 1;
     k03 = 0.02;
     k04 = 0.1;
     k3 = 1;
     k05 = 8;
     k06 = 0.02;
```

k6 = 1;

Diffusivities:

Dms = 1e-2; %Mex5 slow form
Dmf = 1; %Mex5 fast form
Dpks = 1e-2; %Plk1 slow form
Dpkf = 1; %Plk1 fast form
Dpss = 1e-2; %Pos1 slow form
Dpsf = 1; %Pos1 fast form

Parameters for alternative realisation (without threshold behaviour):

k01 = 0.1;
k02 = 0.02;
kpar1 = 1;
k03 = 0.1;
k04 = 0.02;
k3 = 1;
k05 = 0.02;
k06 = 0.1;
k6 = 1;

Diffusivities:

Dms = 1e-3; %Mex5 slow form
Dmf = 1; %Mex5 fast form
Dpks = 1e-2; %Plk1 slow form
Dpkf = 1; %Plk1 fast form
Dpss = 1e-2; %Pos1 slow form
Dpsf = 1; %Pos1 fast form

Parameters for alternative realisation (with threshold behaviour):

k01 = 1;
k02 = 0.02;
kpar1 = 0.01 produces Pos1 gradient; 1 negates the gradient
k03 = 0.15;
k04 = 0.01;
k3 = 0.1;
k05 = 0.02;
k06 = 0.1;
k6 = 1;

```
Diffusivities:

Dms = 1e-3; %Mex5 slow form
Dmf = 1; %Mex5 fast form
Dpks = 1e-2; %Plk1 slow form
Dpkf = 1; %Plk1 fast form
Dpss = 1e-2; %Pos1 slow form
Dpsf = 1; %Pos1 fast form
```

Fig. 9(H,J) and Fig. S9:

For Polo kinase, the parameters values used are

```
kb = 0.4; %binding of inactive form to Map205 in cytoplasm
ku = 0.1; %unbinding from Map205 in cytoplasm

Mt = 1; %Total (uniform) Map205 concentration in cytoplasm
kmp = 0.5; %Polo inactivation in cytoplasm
k02 = 0.1; %basal Polo activation in cytoplasm
ka = 0;%0 or 2, representing Aurora B activation of Polo in cytoplasm
k03=4;
k04=0.5;
k3=5;%%%%% Polo input rate constant to Cdc25
k05=0.01;
k06=0.1;
k5=0.2;%%%%%% Cdc25 input rate constant to cdk1-cycb
k07=0.5;
kcyc = 5;
kn = 0.05;

Dpin = 1e-2; %Inactive Polo
Dpact = 1;   %Active Polo
Dcdcs = 1e-2;%Inactive Cdc25
Dcdcf = 1;   %Active Cdc25
Dcyc0 = 1;   %Inactive CycB complex
Dcyc1 = 1;   %Active fast diffusing CycB complex
Dcyc2 = 1e-2;%Active slow diffusing CycB complex
```

Fig. 9(K): Same equations as 9(D). Both forms active, fast diffusing form more active, activity ratios examined: 0.6 and 0.1

```
k1 = 0.05;
k2 = 10;
k0 = 0.1;
k01 = 0.01;
```

```
Dx = 0.01;
Dy = 1;
```

FIG.9 (L): See supplementary material section 7.2.2 for the detailed analysis. The parameter values and Hill functions used are:

```
xt=1;
yt=1;
x = 0:1e-3:xt;
y = 0:1e-3:yt;

k01 = 0.1;
k02 = 0.2;
k2 = 1;
ky = 0.5;
kdy = 0.2;


L=1;
w=0.5;

g = @(x) 1*(x.^8)./(0.01+x.^8);
h = @(y) 0.6*(y.^8)./(0.05+y.^8);
```
